# Synergistic chemo‑sonic activation for enhanced magnesium extraction from serpentine^☆^

**DOI:** 10.1016/j.ultsonch.2026.108025

**Published:** 2026-08-27

**Authors:** Milad Norouzpour, Rafael M. Santos, Yi Wai Chiang

**Affiliations:** Department of Civil, Environmental, and Water Resources Engineering, University of Guelph, 50 Stone Road East, Guelph, Ontario N1G 2W1, Canada

**Keywords:** Ultramafic tailings, Critical metals, Ultrasound cavitation, Leaching additive, Hydrometallurgy, Waste valorization

## Abstract

Ultrasonic cavitation offers a powerful route to intensify leaching of refractory minerals, yet its effectiveness is often limited by persistent silica-rich transport restriction in silicate systems. Magnesium (Mg) extraction from serpentine is fundamentally constrained by incongruent dissolution and rapid formation of silica-rich alteration layers that suppress mass transport. Here, a coupled chemo‑sonic activation strategy is developed to elucidate how fluoride-mediated silicate destabilization and ultrasound‑driven transport intensification jointly redirect residue evolution toward transport-accessible pore architecture. Comparative leaching experiments were conducted under ambient, thermal, fluoride‑assisted, ultrasonic, and combined operating modes. Conventional acid leaching reached only ∼20% Mg^2+^ extraction, while thermal leaching accelerated early kinetics but plateaued near ∼50% due to silica‑controlled diffusion resistance. Fluoride activation destabilized silica-rich alteration domains, increasing extraction to >80%, whereas ultrasound-assisted leaching alone achieved near‑thermal performance at <40 °C, consistent with enhanced interfacial renewal. Synergistically, their coupled operation induced a non‑linear enhancement, reaching ∼86% extraction within 60 min at low temperature using reduced fluoride dosage. Space–time yield (STY) analysis confirmed genuine process intensification (20.23 g_(__Mg__)_.L^−1^.h^−1^) rather than time‑shifted conversion. Integrated structural and textural characterization demonstrates that enhanced leachability arises from suppression of silica densification and the formation of a transport‑accessible pore architecture rather than surface‑area maximization. These findings demonstrate that chemo‑sonic activation suppresses silica-rich transport restriction and redirects residue evolution toward a transport-accessible, wide-mesopore-dominated architecture, enabling high Mg^2+^ extraction under low-temperature conditions.

## Graphical abstract

**Figure:**
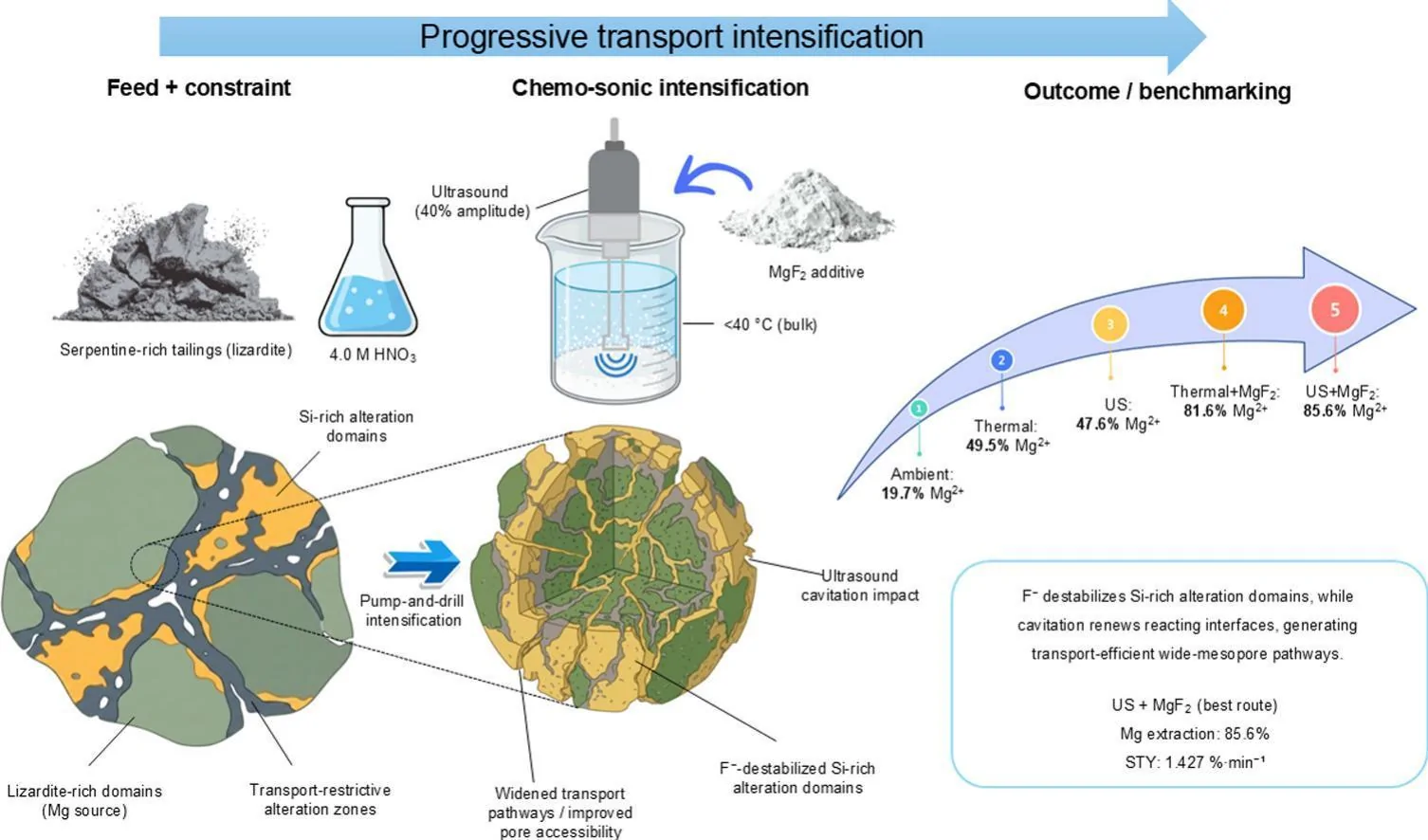

## 1 Introduction

Serpentine-rich ultramafic mine tailings are generated during the extraction and processing of ultramafic ores and are widely stockpiled worldwide, occupying extensive land areas and posing long-term environmental management challenges [1], [2], [3], [4]. The global mining industry generates vast quantities of tailings annually, with reported estimates ranging from approximately 2 × 10^12^ to 6.5 × 10^12^ kg of mining waste annually, while ultramafic/mafic-associated streams, including nickel, asbestos, olivine, and talc tailings contribute substantial annual volumes [2], [5]. From a mineral processing perspective, these materials represent an extreme case of chemically magnesium-rich but kinetically unreactive solids, in which extractable Mg is effectively locked within a structurally resistant, transport-limited silicate matrix. Despite their high Mg content, typically ∼32–38 wt% MgO, these tailings remain largely underutilized, representing both an environmental liability and a significant secondary resource for Mg^2+^ recovery [2], [6], [7]. This apparent contradiction highlights a fundamental challenge in serpentine processing: Mg availability is governed not by bulk composition, but by mineral structure, surface chemistry, and reaction kinetics.

The recovery of Mg from serpentine-rich tailings is relevant not only for waste valorization but also for broader resource-security and decarbonization objectives. Mg and Mg-bearing compounds are important for lightweight alloys, energy-related technologies, and downstream mineralization/carbonation pathways, where dissolved Mg can serve as a precursor for stable carbonate formation [2], [8], [9], [10], [11]. In parallel, increasing demand for critical and strategic minerals has intensified interest in reprocessing mine tailings as secondary resources, particularly as high-grade primary deposits become more constrained and waste-management pressures increase [8], [9], [10], [12]. However, advancing such strategies requires a mechanistic understanding of how serpentine minerals respond to external activation, rather than assuming direct translational feasibility from resource abundance alone. In this context, serpentine minerals (Mg_3_Si_2_O_5_(OH)_4_), particularly lizardite, have attracted attention as promising feedstocks due to their widespread availability and high intrinsic Mg content [7]. At the same time, the layered crystal structure and strong Mg–silicate bonding characteristic of lizardite impose severe limitations on conventional chemical leaching, making serpentine an archetypal refractory Mg-bearing mineral. Accordingly, improving Mg^2+^ extraction from serpentine-rich materials is fundamentally a problem of mitigating silica-rich transport restriction and engineering pore architectures that maintain access to Mg-bearing domains, motivating investigation of process-intensification approaches and their underlying chemo‑physical mechanisms.

The efficient extraction of Mg^2+^ from serpentine is fundamentally constrained by its highly stable layered silicate structure and incongruent dissolution behavior [8]. Serpentine is a 1:1 phyllosilicate composed of alternating brucite-type octahedral sheets and polymerized silica tetrahedral layers, interconnected through strong Si–O–Mg linkages [9], [13], [14]. During acid leaching, Mg is preferentially released from the octahedral layers, while the residual silicate framework reorganizes into a dense, amorphous silica-rich alteration/product layer at the mineral surface [15], [16]. In the present hydrometallurgical context, passivation refers to the development of this heterogeneous silica-rich layer, which progressively restricts mass transport, rather than complete electrochemical inertness or formation of a uniform impermeable shell [15], [17], [18]. As dissolution proceeds, coupled Mg release, silica reorganization, and local silica re-precipitation can densify this alteration layer, limiting inward lixiviant transport and outward Mg diffusion from the unreacted particle interior. As a result, conventional acid leaching of serpentine becomes kinetically sluggish and inefficient, even under relatively high acid concentrations [8], [15]. Similar silica-rich passive/passivation-layer behavior and product-layer diffusion limitations have been reported in fluorite-assisted and conventional serpentine leaching systems, supporting the interpretation that Mg^2+^ recovery is constrained by transport through an evolving silica-rich residue rather than by acid availability alone [15], [19], [20], [21], [22], [23], [24].

To mitigate silica-rich transport restriction, conventional hydrometallurgical strategies have relied on aggressive operating conditions, including elevated temperatures (typically ≥ 80–90 °C) and energy-intensive pretreatments and activation strategies such as calcination [14], [25], [26], microwave heating [27], [28], [29], or intensive grinding [30], [31], [32]. Although these approaches can disrupt the serpentine lattice and enhance initial Mg^2+^ release, they are energy-intensive, operationally complex, and often shift rather than eliminate the kinetic bottleneck [2], [33]. Elevated temperatures and aggressive activation may promote silica condensation and polymerization, generating high-surface-area but poorly connected micro–mesoporous structures that remain diffusion-limited. Thus, systems exhibiting large surface areas may still display poor leachability due to limited pore connectivity and rapid self-passivation [15], [19], [34].

Accordingly, no clearly demonstrated low-severity strategy has yet been shown to simultaneously destabilize the silicate lattice, suppress silica re-densification, and preserve internal mass-transfer accessibility without reliance on bulk heating or extreme pretreatment. Conventional thermal leaching demands high energy input and may promote silica condensation, while mechanical activation can induce particle agglomeration or structural collapse at extended treatment times. Moreover, purely chemical leaching under mild conditions remains fundamentally diffusion-limited due to rapid development of silica-rich transport-restrictive alteration layers [2]. Such limitations highlight the need for a strategy capable of actively destabilizing the silicate framework while dynamically maintaining pore accessibility under controlled, low-temperature conditions. Among alternative strategies, chemical silicate-destabilization approaches have attracted increasing attention to directly destabilize silica-rich alteration layers without relying solely on bulk thermal energy.

One practical route toward this goal is the use of leaching activators that chemically perturb the silicate network during dissolution, thereby limiting silica-rich alteration-layer densification at the reacting interface rather than compensating for it only through bulk heating [9], [15]. Fluoride-bearing additives are particularly effective because F^−^ ions can interact with robust Si–O and Al–O linkages, promote the formation of soluble fluorosilicate and fluoroaluminate species, and limit excessive siloxane polymerization and densification of silica-rich layers [9], [35], [36]. In layered silicate systems, additives such as CaF_2_ and NaF have been reported to widen diffusion pathways and enhance dissolution. Notably, prior fluorite-assisted serpentine leaching work identified fluorosilicate species, particularly ${SiF}_{6}^{2-}$, in solution and interpreted the enhanced Mg^2+^ recovery as arising from fluoride-assisted destabilization of a silica-rich passive/product layer [9], [15], [37], [38], [39]. However, most fluoride-assisted leaching systems reported for serpentine and related silicate minerals remain thermally driven, typically operating above 80 °C, and elevated fluoride-additive dosages can introduce secondary precipitation, interfacial supersaturation, and solution-management challenges when mass transfer is insufficient [9], [36], [40], [41], [42].

Fluoride-assisted acid leaching also has a broader application background beyond serpentine and Mg-silicate systems. In hydrometallurgical and purification processes, fluoride-bearing reagents such as HF, NaF, KHF_2_, NH_4_F, and CaF_2_ have been combined with mineral acids, including HCl and H_2_SO_4_, to enhance dissolution of refractory silicate, aluminosilicate, and oxide-associated phases [39], [43], [44], [45], [46], [47]. Across these systems, fluoride species are generally reported to weaken Si- and Al-bearing frameworks, mitigate silica-rich passivation or gel formation, and improve acid access to encapsulated or structurally bound target metals [35], [43], [44], [45], [46], [47]. Representative fluoride-assisted acid-leaching systems are summarized in Table 1. As shown, most reported systems employ soluble fluoride salts or CaF_2_ under strongly acidic, high-temperature, or pressure-assisted conditions and are primarily directed toward impurity removal or the recovery of metals other than Mg. In contrast, the present work extends fluoride–acid activation to low-temperature Mg^2+^ extraction from lizardite-rich serpentine tailings by coupling a sparingly soluble MgF_2_ activator with ultrasonic cavitation.

**Table 1:** Representative fluoride-assisted acid leaching systems used in critical-material recovery and purification.

Feedstock/target	Acid–fluoride system	Main reported role of fluoride species	Reference
Natural microcrystalline graphite/ash impurity removal	HCl–NaF	Removes silicate/oxide ash impurities and increases fixed carbon content	[43]
Aphanitic graphite/ ash impurity removal	Dual-frequency ultrasound + HCl–KHF_2_	Combines fluoride-assisted impurity dissolution with ultrasound-enhanced mass transfer and particle fragmentation, thereby removing silica-bearing impurities more efficiently	[44]
Manganotantalite/ Ta–Nb extraction	H_2_SO_4_–NH_4_F pressure leaching	NH_4_F assists decomposition of refractory Ta–Nb oxide structure and improves Ta/Nb leaching efficiency	[45]
Stone coal/ vanadium extraction	H_2_SO_4_–CaF_2_	Promotes decomposition of silicate/aluminosilicate host phases through fluorosilicate and fluoroaluminate complex formation, increasing vanadium extraction	[35]
Black shale/ vanadium extraction	H_2_SO_4_– CaF_2_	Enhances vanadium release from aluminosilicate-bearing phases by weakening Al/Si-containing mineral frameworks	[36]
Refractory oxidized copper ore/Cu extraction	H_2_SO_4_–CaF_2_	Improves copper leaching by disrupting Si–O and Al–O bonds in the crystal structure of biotite and phlogopite	[42]
Serpentine tailings/ Mg^2+^ extraction	H_2_SO_4_–CaF_2_	Enhances Mg^2+^ recovery by mitigating silica-rich passivation and promoting serpentine lattice destabilization	[9], [15]
Coal fly ash/Al extraction	H_2_SO_4_–NaF	Assists dissolution of the refractory mullite/aluminosilicate matrix and promotes alumina release	[46]
Phosphorus–potassium associated ore/K–Al extraction	HCl–CaF_2_	Promotes K-feldspar decomposition and formation of soluble metal chlorides and silicon-fluoride species	[47]
Lizardite-rich serpentine tailings/ Mg^2+^ extraction	HNO_3_–MgF_2_ + ultrasound	Couples sparingly soluble MgF_2_-derived fluoride release with ultrasound-associated interfacial renewal and particle/pore modification under low-temperature conditions.	This work

Complementary to chemical silicate-destabilization approaches, ultrasonic cavitation offers a primarily physical route to process intensification by delivering energy selectively at the solid–liquid interface [48]. Because acoustic energy is concentrated locally through cavitation events rather than applied as bulk thermal input, ultrasound-assisted systems can reduce overall thermal energy demand and potentially lower the associated process carbon footprint when appropriately optimized [48], [49], [50]. The collapse of cavitation bubbles generates localized micro-jets and shockwaves that erode surface films, renew boundary layers, and enhance intra-particle transport by forcing lixiviant into micro-cracks and pores [48], [51], [52]. Although ultrasound has been successfully applied to accelerate leaching in several refractory metallurgical systems [48], its application to Mg silicates remains limited. More importantly, cavitation alone does not fundamentally alter the chemical pathway of silica re-polymerization and therefore remains constrained by silica-rich transport restriction when dissolution proceeds in silica-saturated matrices, indicating that physical intensification without concurrent chemical control is insufficient [37].

In this study, the intrinsic limitations of independent chemical and physical treatment strategies are addressed through their deliberate integration within an engineered activation framework. Here, engineered activation refers to a coordinated chemical and external-field-assisted approach in which fluoride-mediated silicate destabilization is coupled with ultrasonication to actively sustain transport-accessible pathways during leaching. Within the proposed “*pump-and-drill*” concept, fluoride acts as a chemical drill that destabilizes the silicate scaffold and suppresses silica densification, while ultrasonic cavitation functions as an acoustic pump that continuously renews the solid–liquid interface and enhances reagent penetration into near-surface and internal pore domains. Distinct from prior studies that relied primarily on CaF_2_ or soluble fluoride salts under thermally driven conditions, this work evaluates MgF_2_ as a sparingly soluble fluoride source under low-temperature chemo-sonic conditions, enabling acid-promoted in situ fluoride release coupled with cavitation-driven interfacial renewal. To the best of our knowledge, MgF_2_ has not been systematically examined as a controlled fluoride activator within a transport-engineered Mg^2+^ recovery framework for serpentine-rich ultramafic tailings.

More importantly, this study evaluates whether that coupling fluoride-mediated silicate destabilization with cavitation-assisted interfacial renewal can redirect leaching from silica-controlled, transport-limited dissolution toward a transport-enabled extraction pathway under mild operating conditions. Using lizardite-rich serpentine tailings as a representative ultramafic feedstock, the coupled effects of fluoride chemistry and acoustic cavitation on Mg^2+^ extraction behaviour, apparent leaching kinetics, pore-architecture evolution, particle/surface morphology, and fluoride distribution are systematically evaluated. Unlike prior investigations that evaluate chemical or physical strategies independently, this work benchmarks their interaction using STY analysis and screening-level electricity/cost indicators to distinguish genuine process intensification from apparent conversion gains. By integrating kinetic evaluation with advanced structural characterization, including Brunauer–Emmett–Teller (BET) surface analysis, non-local density functional theory (NL-DFT) pore modeling, X-ray diffraction (XRD), particle size distribution (PSD), Fourier transform infrared spectroscopy (FT-IR), scanning electron microscopy (SEM), and Frenkel–Halsey–Hill (FHH) fractal analysis, the mechanistic origins of enhanced leachability are elucidated are interpreted through complementary evidence across molecular, pore, particle, and residue-surface scales.

This mechanistic framework is supported by convergent evidence from complementary characterization methods rather than by any single direct measurement. Direct nanoscale characterization, including atomic force microscopy (AFM) surface-roughness mapping and focused ion beam–scanning electron microscopy (FIB-SEM) cross-sectioning coupled with energy-dispersive X-ray spectroscopy (EDS) compositional line scanning or mapping of Si and Mg, was not performed in the present study; consequently, alteration-layer thickness, local compositional gradients, and nanoscale interfacial damage were not directly resolved and are identified as key targets for future verification. The combined results indicate that Mg^2+^ extraction is more closely associated with transport-accessible pore architecture, wide-mesopore development, surface regularization, and lamellar-edge exposure rather than cumulative surface area alone, thereby resolving the high-surface-area paradox observed in thermally intensified systems. Collectively, these findings establish a transport-centered engineered chemo-sonic activation strategy for Mg^2+^ recovery from serpentine-rich ultramafic tailings.

## 2 Experimental methodology

### 2.1 Materials and reagents

**Fig. 1:**
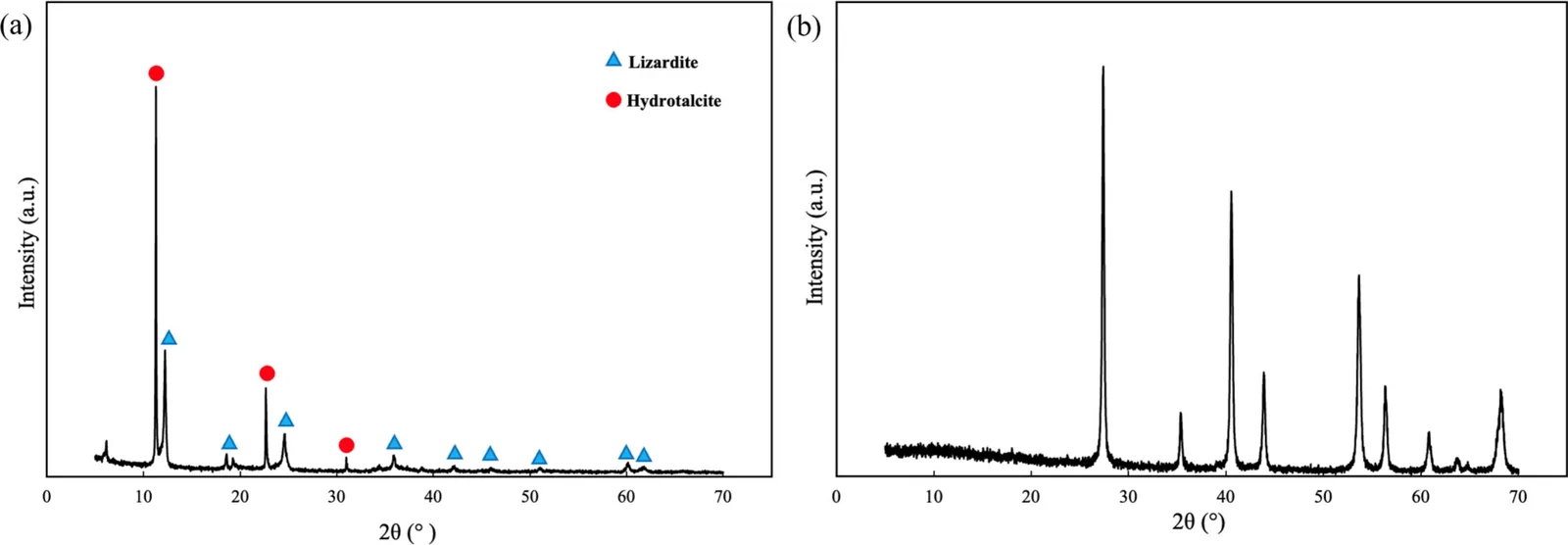
XRD patterns of (a) serpentine-rich ultramafic mine tailings, showing lizardite as the dominant phase with minor hydrotalcite-type layered double hydroxides, and (b) as-received MgF_2_ additive confirming phase purity.

Serpentine-rich ultramafic mine tailings were obtained from a Canadian mining operation and used as the Mg-bearing feedstock in this study. XRD analysis confirmed that the material is predominantly composed of lizardite (Mg_3_Si_2_O_5_(OH)_4_), with minor amounts of hydrotalcite-type Mg–Al–Fe layered hydroxides, as shown in Fig. 1a.

The as-received samples were initially dried at 105 °C overnight to remove residual moisture and then lightly ground to disaggregate weakly bound particles. Size reduction was carried out using a microfine impact mill (MF 10 basic, IKA-Werke GmbH, Germany) equipped with a stainless-steel impact grinding head. The milled material was passed through an internal sieve to obtain a uniform particle size fraction, which was used for all leaching and characterization experiments. This particle size fraction was selected to balance sufficient external surface exposure with industrially realistic comminution energy input.

Bulk chemical composition was determined by wavelength-dispersive X-ray fluorescence (WDXRF), revealing that the material contains approximately 39.17 wt% MgO and 30.02 wt% SiO_2_, with the full oxide composition summarized in Table 2. The total Mg content corresponds to ∼23.62 wt% on a mass basis, confirming the suitability of the sample for Mg^2+^ extraction and downstream utilization. Nitrogen (N_2_) physisorption and laser diffraction analyses indicated a relatively low initial BET specific surface area of 15.70 ± 0.01 m^2^ g^−1^ and a broad volume-based PSD, as summarized in Table 3. These measurements were used to establish the baseline textural and particle-size characteristics of the feedstock prior to leaching.

**Table 2:** The elemental composition (reported as oxide equivalents determined by WDXRF) of the sample used in this study.

Element	SiO_2_	MgO	Al_2_O_3_	Fe_2_O_3_	CaO	TiO_2_	P_2_O_5_	Na_2_O	MnO	LOI
Mass fraction (%)	30.02	39.17	6.83	4.94	0.80	0.14	0.06	0.09	0.01	17.75

**Table 3:** PSD metrics and volume-based size fractions of raw lizardite tailings.

Particle size metrics	Diameter (μm)	Particle size range (μm)	Volume (%)
D10	4.74	<63	59.55
D50	41.8	63–125	13.95
D90	227	125–250	20.92
D [2,3]	11.2	250–500	5.58
D [3,4]	83.8	Total	100

Magnesium fluoride (MgF_2_, ≥99% purity) was employed as the fluoride source and obtained from MSE Supplies LLC (Tucson, USA). XRD analysis of the as-received additive confirmed MgF_2_ as the dominant crystalline phase, with diffraction peaks consistent with reported MgF_2_ reference patterns and no detectable secondary impurities (Fig. 1b) [53], [54], [55]. The high phase purity ensured controlled fluoride release during leaching without interference from secondary reactive impurities.

Reagent-grade HNO_3_ (68–70 wt%) was purchased from Fisher Scientific (Canada) and diluted with ultrapure Milli-Q water (18.2 MΩ cm resistivity) to prepare leaching solutions with concentrations of 0.5, 1.0, 2.0, and 4.0 M, as required. HNO_3_ was selected as the lixiviant to provide a controlled nitrate-based leaching medium for evaluating the coupled effects of MgF_2_ addition and ultrasonic cavitation on Mg^2+^ extraction. The objective was not to screen all possible industrial acid systems, but to establish the mechanistic and engineering response of lizardite-rich serpentine under a defined HNO_3_–MgF_2_ thermal and ultrasound-assisted leaching framework. The applicability and limitations of transferring this activation strategy to H_2_SO_4_- or HCl-based systems are discussed in Section 3.9. Ultrapure water was used throughout all experiments to minimize background ionic interference and ensure reproducible leaching measurements.

### 2.2 Leaching experiments

Batch leaching experiments were conducted to evaluate the effects of acid concentration, temperature, ultrasonic cavitation, and fluoride addition on Mg^2+^ extraction from lizardite-rich tailings. In each experiment, 10.0 g of dried mineral sample was mixed with 100 mL of HNO_3_ solution, corresponding to a solid–liquid ratio of 1:10 (w/v). Under the highest acid concentration (4.0 M HNO_3_), the total proton availability substantially exceeded the stoichiometric requirement for complete Mg dissolution, ensuring that leaching behavior was not acid limited. All tests were performed in a 250 mL three-neck glass flask equipped with mechanical agitation and operated at a constant stirring speed of 200 rpm to ensure uniform suspension of solids.

Conventional leaching experiments were carried out at controlled temperatures of 25 °C (ambient), 45 °C, 65 °C, and 85 °C using HNO_3_ concentrations of 0.5, 1.0, 2.0, and 4.0 M to establish baseline dissolution behavior and identify optimal leaching conditions. To minimize solvent loss at elevated temperatures, experiments were performed under a reflux condenser to prevent acid evaporation and maintain constant solution composition. Ambient-temperature leaching tests were conducted for up to 360 min to determine the characteristic extraction plateau, after which a maximum leaching time of 180 min was adopted for subsequent thermally assisted experiments. Liquid aliquots were withdrawn at selected time intervals for Mg^2+^ analysis. At the end of each experiment, solid residues were recovered by vacuum filtration, washed repeatedly with Milli-Q water until neutral pH was achieved, and dried at 105 °C for subsequent characterization.

Ultrasound-assisted leaching experiments were performed using a probe-type ultrasonic processor (SONICS Vibra-Cell, model VCX750) operating at a fixed frequency of 20 kHz with a maximum nominal power rating of 750 W. The ultrasonic horn, fabricated from a corrosion-resistant titanium alloy (model CV334), was immersed directly into the slurry and operated at an amplitude of 40%. Because ultrasonic operating parameters can influence leaching performance in ultrasound-assisted leaching systems [56], the amplitude used in this study was selected based on a preliminary amplitude-screening experiment. This screening was conducted under MgF_2_-free leaching conditions at 4.0 M HNO_3_, a fixed sonication time of 60 min, pulse mode of 5 s on / 1s off, and bulk temperature maintained below 40 °C; the corresponding screening results are provided in Fig. S1. Mechanical agitation at 200 rpm was applied simultaneously using a magnetic stirrer to ensure homogeneous exposure of the slurry to cavitation. To limit bulk-temperature rise during sonication, the system was externally cooled, and slurry temperature was continuously monitored and maintained below 40 °C to minimize conventional bulk-temperature contributions. Ultrasound-assisted leaching experiments were conducted using HNO_3_ concentrations ranging from 0.5 to 4.0 M with leaching times of up to 60 min. A maximum sonication time of 60 min was selected to evaluate ultrasound-assisted extraction enhancement within a standardized reaction-time window, enabling direct time-matched comparison with thermally activated systems. The objective of these experiments was to assess process intensification under controlled operating conditions rather than to pursue equilibrium conversion.

MgF_2_-assisted leaching was investigated by adding MgF_2_ as a fine powder at dosages of 0–15 wt% relative to the dry mineral mass. In fluoride-containing experiments, MgF_2_ was introduced after an initial 3 min pre-mixing period to establish a stable baseline dissolution environment prior to fluoride activation. Fluoride-enhanced leaching was evaluated under thermal conditions at 45, 65, and 85 °C and ultrasound-assisted conditions at controlled bulk temperature below 40 °C, while all other relevant operating parameters were held constant.

All leaching experiments were performed in triplicate and reported Mg^2+^ extraction values represent mean values with corresponding standard deviations. Error bars in figures represent one standard deviation of triplicate measurements. Dissolved Mg^2+^ concentrations in the leachates were quantified by inductively coupled plasma mass spectrometry (ICP-MS), and extraction efficiencies were calculated based on the initial Mg content of the feedstock determined by WDXRF analysis. Solid residues were retained for complementary structural, textural, and morphological characterization, as detailed in the following section.

The overall experimental workflow is summarized in Fig. S2. Briefly, raw lizardite tailings were prepared by drying, grinding, and particle-size control, followed by leaching in HNO_3_ systems with optional MgF_2_ addition under thermal or ultrasound-assisted conditions. After leaching, solid residues and liquid phases were separated and subjected to complementary solid-state characterization and liquid-phase analysis, as detailed in Section 2.3.

### 2.3 Characterization techniques

The characterization strategy was organized to relate feedstock composition and route-dependent structural evolution to leaching performance through a progressive analytical sequence. Bulk composition was first determined by WDXRF to establish the initial Mg inventory and oxide composition. N_2_ physisorption combined with BET/NL-DFT and FHH analysis was then used to characterize accessible surface area, pore-size and pore-volume evolution, and nanoscale pore-wall irregularity. FT-IR and XRD were employed to evaluate silicate-network restructuring and crystallographic evolution, respectively. PSD analysis, SEM imaging, and semi-quantitative SEM/ImageJ analysis provided complementary particle-size and surface-morphology descriptors of leaching-induced modification. Dissolved Mg and selected Al concentrations were quantified by ICP-MS, while selected leachates and residue extracts were analyzed by ion chromatography to evaluate operationally measured fluoride. Kinetic and engineering calculations are presented separately in 2.5, 2.6, respectively.

#### 2.3.1 WDXRF analysis

The bulk chemical composition of the raw sample was determined by WDXRF using a Rigaku Supermini200 benchtop sequential spectrometer equipped with a 200 W Pd-target X-ray tube operated at 50 kV and 4 mA. Prior to analysis, dried samples were finely ground to ensure homogeneity and prepared as pressed pellets by mixing the powder with a small amount of binder and compacting under hydraulic pressure to form mechanically stable briquettes. The pellets were analyzed under vacuum conditions to enhance the detection of light elements. Quantitative analysis was performed using the SQX (Scan Quant X) fundamental parameters (FP) software with appropriate matrix corrections. Elemental concentrations are reported as oxide equivalents (wt%), and loss on ignition (LOI) was determined separately and included to ensure full mass closure. The measured bulk Mg content was used as the stoichiometric reference for calculation of extraction efficiencies in subsequent leaching experiments.

#### 2.3.2 N_2_ Physisorption, BET/NL-DFT and FHH analysis

N_2_ adsorption–desorption isotherms were measured at 77 K using a Quantachrome Autosorb iQ gas sorption analyzer to determine the specific surface area, total pore volume, and the pore size distributions of raw and leached solids. Prior to analysis, approximately 100–250 mg of each sample was degassed under vacuum using the instrument’s integrated outgassing stations to remove physically adsorbed moisture and volatile species. Degassing was performed at 250 °C for 6 h until a pressure-rise limit ≤50 µmHg min^−1^ was reached, as monitored by the ASiQwin software.

Following pretreatment, samples were transferred to the analysis station and immersed in a liquid N_2_ bath. The free space, or void volume, of each sample cell was determined using helium. The specific surface area was calculated using the multipoint BET method. Total pore volume was determined from the amount of N_2_ adsorbed at P/P_0_ ≈ 0.99, assuming complete filling of accessible pores [57].

Pore size distributions were obtained using NL-DFT modelling, which provides a molecular-level description of adsorption in micro- and mesoporous materials and is more appropriate than classical macroscopic thermodynamic models (e.g., BJH) for heterogeneous silicate surfaces [58], [59]. NL-DFT pore size analysis was performed on the N_2_ adsorption–desorption isotherms using an equilibrium model with a silica cylindrical-pore kernel implemented in ASiQwin. This approach enabled evaluation of N_2_-accessible pore-size distributions and pore-structure evolution induced by thermal leaching, ultrasonic irradiation, and MgF_2_-assisted leaching. All data acquisition and reduction were conducted using the ASiQwin software package.

To provide a complementary descriptor of nanoscale pore-wall irregularity, the FHH surface fractal model was applied to the N_2_ adsorption branch [60], [61]. The resulting surface-fractal dimension (D_s_) provides a dimensionless heuristic descriptor of nanoscale pore-wall roughness/geometric irregularity within the selected fitting regime.

For each sample (RS, A-opt, T-opt, TF-opt, U-opt, UF-opt), the adsorption branch was isolated, and the FHH relationship was fitted within the low-pressure window 0.05 ≤ P/P_0_ ≤ 0.30. This range falls within the low-relative-pressure regime commonly associated with adsorption dominated by solid–fluid van der Waals interactions and surface roughness, where contributions from capillary condensation and pore-filling effects are minimized, consistent with established interpretations of the FHH surface-fractal regime [61], [62]. Within this window, the linearized FHH expression is given by [62], [63]:

(1) $$\ln\left(V\right)=C+\left(D_{s}-3\right)\ln\left[ln(\frac{P_{0}}{P})\right]$$

where V is the adsorbed volume at STP (cm^3^ g^−1^), P/P_0_ is the relative pressure, and C is a constant. The fitted slope corresponds to (D_s_ − 3), and because the slope is negative in the linearized FHH representation, D_s_ is obtained as D_s_ = 3 + slope [61], [62], [64]. Fractal dimension values in porous media typically range between 2 and 3, where values closer to 3 indicate greater nanoscale pore-wall irregularity, whereas values approaching 2 correspond to comparatively smoother pore walls [65]. D_s_ is used here as a heuristic descriptor of pore-wall roughness/geometric irregularity within the selected fitting regime rather than as a direct measurement of tortuosity or three-dimensional pore connectivity.

#### 2.3.3 FT-IR analysis

FT-IR spectroscopy was employed to probe silicate-network evolution and hydroxyl-group modifications associated with fluoride-mediated silicate destabilization and structural reorganization during leaching. FT-IR spectra of the raw and leached solid samples were collected using a Thermo Scientific Nicolet 6700 spectrometer equipped with a Smart iTR attenuated total reflectance (ATR) accessory with a single-bounce diamond crystal and a KBr beamsplitter. The instrument was continuously purged with dry N_2_ to minimize interference from atmospheric H_2_O and CO_2_. Finely powdered samples were placed directly onto the ATR crystal and pressed using the built-in pressure tower to ensure consistent contact. A background spectrum was recorded on the clean crystal prior to each measurement. Spectra were acquired over the wavenumber range of 4000–600 cm^−1^ at a resolution of 4 cm^−1^, with 128 co-added scans per sample to improve the signal-to-noise ratio. All spectra were collected in absorbance mode and processed using the OMNIC software package.

#### 2.3.4 XRD analysis

XRD was used to identify crystalline phases present in the lizardite sample and leached solid residues. Measurements were carried out using a Malvern Panalytical Empyrean multipurpose diffractometer operating with Cu Kα radiation (λ = 1.5406 Å). Prior to analysis, solid samples were finely ground using an agate mortar and pestle and loaded into a sample holder. The specimens were analyzed using a spinner stage to minimize preferred orientation effects and improve particle statistics. Diffraction patterns were collected over a 2θ range of 5–70° under continuous scan mode with a total acquisition time of approximately 20 min per sample. Phase identification and data processing were performed using HighScore software by matching diffraction peaks with reference patterns from the ICDD database. Variations in peak intensity, peak broadening, and baseline elevation were qualitatively assessed to evaluate lattice destabilization and progressive loss of crystallinity following various treatments. Additional cross-verification with established mineralogical databases was conducted where necessary [66].

#### 2.3.5 PSD analysis

The PSD of the raw lizardite tailings was determined by laser diffraction using a Malvern Mastersizer 3000 equipped with an Aero S dry dispersion unit. Dried samples were dispersed under controlled airflow through a venturi-based system to promote de-agglomeration while minimizing particle breakage. Measurements were conducted under stabilized laser obscuration conditions to ensure reproducibility. Scattering data were analyzed using the Mie model implemented in the Mastersizer software with optical parameters assigned to lizardite. Particle size distributions are reported on a volume basis. The raw tailings exhibited a broad size distribution, with characteristic diameters (D10, D50, D90) summarized in Table 3. Notably, 59.55 vol% of particles were below 63 µm, indicating a predominantly fine-grained feedstock with relatively high geometric surface exposure prior to activation.

To compare early-stage particle-size modification under ultrasound-assisted and non-ultrasound leaching routes, an additional set of 30 min validation residues (T30, U30, TF30, UF30) and a remeasured raw aliquot (RS, internal baseline) were analyzed by laser diffraction using the same dry-dispersion method. Details of the validation sample set and preparation conditions are provided in Text S1.

#### 2.3.6 SEM and semi-quantitative SEM/ImageJ analysis

The surface morphology and microstructural features of the raw and leached solid samples were examined using a field-emission scanning electron microscope (FEI Quanta FEG 250). Prior to imaging, dried powder samples were mounted on aluminum stubs using conductive carbon tape. To minimize surface charging under high-vacuum conditions, the samples were sputter-coated with an approximately 3–5 nm conductive Pt/Pd layer. This coating thickness was selected to provide sufficient conductivity while minimizing masking of fine surface features. SEM imaging was performed primarily in secondary electron (SE) mode using an Everhart–Thornley detector (ETD) to resolve surface topography and near-surface morphological features. Images were acquired at accelerating voltages in the range of 10–15 kV and at magnifications between 10,000× and 100,000× to capture both particle-scale morphology and fine surface features. Instrument control and image acquisition were conducted using the xT microscope control software.

To provide semi-quantitative support for the SEM-based morphology interpretation, selected high-contrast 10,000× SEM fields were analyzed using Fiji/ImageJ [67]. The analysis was applied to the early-stage 30 min validation residues (T30, U30, TF30, and UF30) and to the near-equivalent-extraction MgF_2_-free residues (T-opt and U-opt), with five fields analyzed per condition. Only fields without severe charging artifacts, poor focus, excessive shadowing, scale-bar/footer interference, or obvious threshold over-detection were included in the analysis [68]. Images were scale-calibrated using the embedded scale bars and cropped only to remove scale bars, labels, footers, and non-image borders.

A common preprocessing and segmentation workflow was applied to all analyzed images. The workflow comprised conversion to 8-bit format, contrast normalization, noise filtering, edge detection, thresholding using the Triangle auto-threshold algorithm, binarization, skeletonization, and subsequent Analyze Skeleton measurements. Representative processing examples are provided in Fig. S3.

Two semi-quantitative descriptors were calculated from the skeletonized fields. The apparent SEM edge-density index, $D_{\text{edge}}$(µm^−1^), was calculated by normalizing the total skeletonized branch length to the analyzed image area:

(2) $$D_{\text{edge}}=\frac{{\Sigma L}_{\text{branch}}}{A_{\text{image}}}$$

where ${\Sigma L}_{\text{branch}}$ is the total skeletonized branch length and $A_{\text{image}}$ is the calibrated analyzed SEM image area. The branch-point density, *D*_branch_ (junctions µm^−2^), was calculated by normalizing the total number of skeleton junctions by the analyzed image area:

(3) $$D_{\text{branch}}=\frac{{\Sigma N}_{\text{junctions}}}{A_{\text{image}}}$$

where ${\Sigma N}_{\text{junctions}}$ is the total number of skeleton junctions obtained from the skeleton analysis.

These descriptors were used as supportive semi-quantitative indicators of visible surface-edge development, branch-point/corner complexity, lamellar exposure, and fragmentation-related surface modification. They were not interpreted as absolute crack-density, inert-layer-thickness, or true three-dimensional pore-connectivity measurements because SEM contrast and skeletonization response can be influenced by particle orientation, surface topography, thresholding behavior, and shadowing [68].

#### 2.3.7 ICP-MS analysis

Dissolved Mg concentrations in leachate samples were quantified using an ICP-MS (NexION 2000, PerkinElmer, USA) equipped with Universal Cell Technology (UCT). The plasma was operated at an RF power of 1600 W. Leachates were filtered through 0.45 µm membranes, appropriately diluted, and acidified to 2 vol% HNO_3_ prior to analysis. Measurements were performed in Collision mode (Kinetic Energy Discrimination, KED) using helium gas to suppress polyatomic interferences associated with nitrate- and silicate-rich matrices. Mg was quantified using the ^24^Mg isotope, with scandium (Sc) employed as an internal standard to correct for nonspectral interferences, instrumental drift, and matrix effects. Calibration was carried out using multi-point external standards prepared from certified single- or multi-element standard solutions. For selected leachates from MgF_2_-assisted thermal and ultrasound-assisted experiments, dissolved Al was also quantified using the ^27^Al isotope under the same ICP-MS analytical workflow to evaluate non-Mg co-leaching behavior and its possible influence on effective fluoride availability. The method detection limit for Mg was at least one order of magnitude lower than the minimum concentration measured in baseline leaching experiments. Mg and Al extraction yields were calculated based on the initial Mg and Al contents of the feedstock determined by WDXRF analysis. For experiments involving MgF_2_ addition, blank leaching tests were conducted under identical acid concentration and temperature conditions in the absence of lizardite sample, and the Mg contribution from MgF_2_ dissolution was subtracted to ensure that reported extraction values exclusively reflect Mg released from the lizardite matrix.

#### 2.3.8 Ion chromatography analysis of fluoride

Fluoride concentrations in selected leachates and solid extracts were quantified using a Metrohm Eco IC ion chromatograph at the Morwick G360 Institute for Groundwater Research. Selected final leachates were separated from the solid phase, filtered, diluted as required, and analyzed for F^−^. The reported fluoride concentrations are dilution-corrected. To provide a screening-level assessment of solid-associated fluoride, selected washed and dried solids, including the raw feedstock and MgF_2_-assisted residues, were subjected to an operational alkaline extraction adapted from published fluoride-contaminated soil washing/extractability approaches, in which NaOH has been evaluated as an alkaline washing/extraction reagent for fluoride-bearing solids [69], [70]. Briefly, 0.5000 g of dried solid was contacted with 50.00 mL of 0.5 M NaOH, heated at 50–60 °C, cooled, and clarified by filtration prior to IC analysis. A Milli-Q water quality-control sample and a NaOH extraction blank were also analyzed. Because this procedure was used as an operational extractability test rather than complete solid digestion, fusion, or combustion IC, the solid-side results are reported as NaOH-extractable F^-^ rather than total fluorine. Complete determination of total fluorine in resistant solid matrices generally requires stronger digestion or fusion-based preparation, such as alkaline fusion, and therefore was outside the scope of this screening-level assessment [70], [71].

### 2.4 Data analysis and reproducibility

All experiments were performed in triplicate, and results are presented as mean ± standard deviation (n = 3). Statistical analyses were performed using Python (statsmodels library). One-way analysis of variance (ANOVA) followed by Tukey’s honestly significant difference (HSD) post-hoc test was used to evaluate statistically significant differences among experimental conditions (*p* < 0.05). For clarity, statistical groupings are shown only for Fig. 6.

### 2.5 Kinetic analysis and apparent activation energy calculation

The leaching kinetics of Mg^2+^ extraction were evaluated using standard solid–liquid reaction models derived from the shrinking-core framework, including product-layer diffusion control, surface chemical reaction control, liquid-film diffusion control, combined product-layer diffusion and chemical reaction control, and first-order pseudo-homogeneous kinetics [72], [73]. The conversion variable, *x*, was defined as the fraction of Mg^2+^ extracted at time (*t*) relative to the total Mg content of the feedstock determined by WDXRF. The experimental extraction data were fitted to the following kinetic expressions [15], [74], [75]:

Product-layer diffusion control:

(4) $${\mathrm{kt}=1-3\;(1-x)}^{2/3}+2\;\left(1-x\right)$$

Surface chemical reaction control:

(5) $${\mathrm{kt}=1-\;(1-x)}^{1/3}$$

Liquid-film diffusion control:

(6) kt=x

Combined product-layer diffusion and chemical reaction control:

(7) $${\mathrm{kt}=(1-3\;(1-x)}^{2/3}+2\;\left(1-x\right))+{\left(1-(1-x\right)}^{1/3}\;)$$

First-order pseudo-homogeneous kinetics:

(8) kt=-ln(1-x)

For each model, the experimental data were transformed according to the corresponding kinetic expression and plotted against reaction time; the apparent rate constant, *k*, was obtained from the slope of the fitted line, and the coefficient of determination, $R^{2}$, was used to evaluate goodness of fit. Apparent activation energies were determined using the Arrhenius relationship:

(9) $$k=A\;exp(-\frac{E_{a}}{\mathrm{RT}}\;)\;\;$$

where *A* is the pre-exponential factor, *E*_a_ is the apparent activation energy, *R* is the universal gas constant, and *T* is absolute temperature in Kelvin. The apparent activation energy, *E_a_*, was obtained from the Arrhenius equation by plotting ln *k* against *1/T*, where T is temperature in Kelvin. Kinetic analysis was performed for the conventional HNO_3_ and MgF_2_-assisted thermal systems at 45, 65, and 85 °C using temperature-series data at 4.0 M HNO_3_. Because the ultrasound-assisted experiments were conducted within a controlled bulk-temperature window below 40 °C to minimize conventional bulk-temperature contributions rather than as a temperature series, an ultrasound-specific Arrhenius analysis and apparent activation energy were not determined. The complete model-fitting results, including the fitted rate constants and coefficients of determination for all evaluated models, are provided in the corresponding Supplementary Information Tables S3 and S4.

### 2.6 Engineering performance and screening-level cost calculation

Screening-level electricity-consumption, productivity, and leaching-stage input-cost indicators were calculated for the main activation routes: A-opt, T-opt, TF-opt, U-opt, and UF-opt. Electricity consumption was measured as bench-scale wall-plug/circuit-level input using an Emporia Vue Gen 3 energy monitor (Emporia Corp., model EMV3A-2P-8) equipped with clamp-on current-transformer sensors and therefore does not represent delivered acoustic energy, delivered thermal energy, or calorimetrically measured power input. Thermal-route values included heating and stirring, including heat-up energy, whereas ultrasound-assisted values included sonication and stirring during reaction. Active cooling electricity was not included because cooling was applied passively to maintain the slurry below 40 °C.

The mass of Mg recovered in solution was calculated as:

(10) $$m_{Mg,\;recovered}=m_{feed}\;w_{Mg\;}\left(\frac{X_{Mg}}{100}\right)$$

where $m_{feed}$ is the initial mineral mass, $w_{Mg}$ is the Mg mass fraction of the feedstock derived from WDXRF, and $X_{Mg}$ is the measured Mg^2+^ extraction in percent. For the standard 10 g mineral charge used in this study, the feed Mg content of 23.62 wt% corresponds to 2.362 g of initial Mg.

Specific electricity consumption (SEC) was calculated by normalizing the measured electricity input to the mass of Mg recovered:

(11) $$\mathrm{SEC}=\frac{E_{elec}}{m_{Mg,\;recovered}}$$

where $E_{elec}$ is the measured wall-plug electricity consumption (kWh) and $m_{Mg,\;recovered}$ is the mass of Mg recovered (kg), yielding SEC in kWh kg^−1^ Mg recovered.

A normalized productivity index was defined as the cumulative Mg^2+^ extraction divided by the corresponding residence time:

(12) $${\mathrm{STY}}_{\mathrm{norm}\;}=\;\frac{X_{\mathrm{Mg}}}{t_{\min}}$$

where $X_{\mathrm{Mg}}$ is Mg^2+^ extraction in percent and $t_{\min}$ is leaching time in minutes, yielding ${\mathrm{STY}}_{\mathrm{norm}}$ in % min^−1^. This metric represents average extraction productivity over the selected residence time and does not imply constant reaction-rate behavior.

To provide a scale-relevant volumetric throughput metric, an absolute STY was calculated as [76]:

(13) $${\mathrm{STY}}_{\mathrm{abs}\;}=\;\frac{m_{Mg,\;recovered}}{V_{liq}\;\;t_{h}}$$

where $m_{Mg,\;recovered}$ is the mass of recovered Mg (g), $V_{liq}$ is the liquid volume (L), and $t_{h}$ is the leaching time in hours, yielding ${\mathrm{STY}}_{\mathrm{abs}}$ in g_(__Mg__)_ L^−1^h^−1^.

Screening-level leaching-stage input cost was estimated from measured electricity and reagent inputs, including HNO_3_ and MgF_2_, using bulk/commercial price assumptions for relative comparison among the tested routes. These values exclude capital cost, labor, reagent recycling, downstream Mg recovery/purification, wastewater treatment, residue handling, and conversion to Mg metal; therefore, they are interpreted only as relative leaching-stage indicators, not full techno-economic or commercial Mg production-cost estimates. Supporting measured electricity data and screening-level cost-calculation outputs are provided in Tables S9 and S10.

## 3 Results and discussion

This section systematically evaluates Mg^2+^ extraction from lizardite-rich serpentine tailings across progressively intensified activation regimes. Conventional acid leaching is first established as the baseline transport-limited pathway, followed by assessment of thermal leaching, fluoride-mediated silicate destabilization via MgF_2_ addition, and physical intensification through ultrasonic cavitation. The individual and combined effects of these strategies are analyzed to elucidate the evolution from conventional transport-limited leaching toward a more transport-enabled extraction pathway. Results are interpreted in the context of apparent leaching kinetics, interfacial mass-transfer constraints, and structural evolution of the solid residue.

### 3.1 Conventional baseline leaching performance

**Fig. 2:**
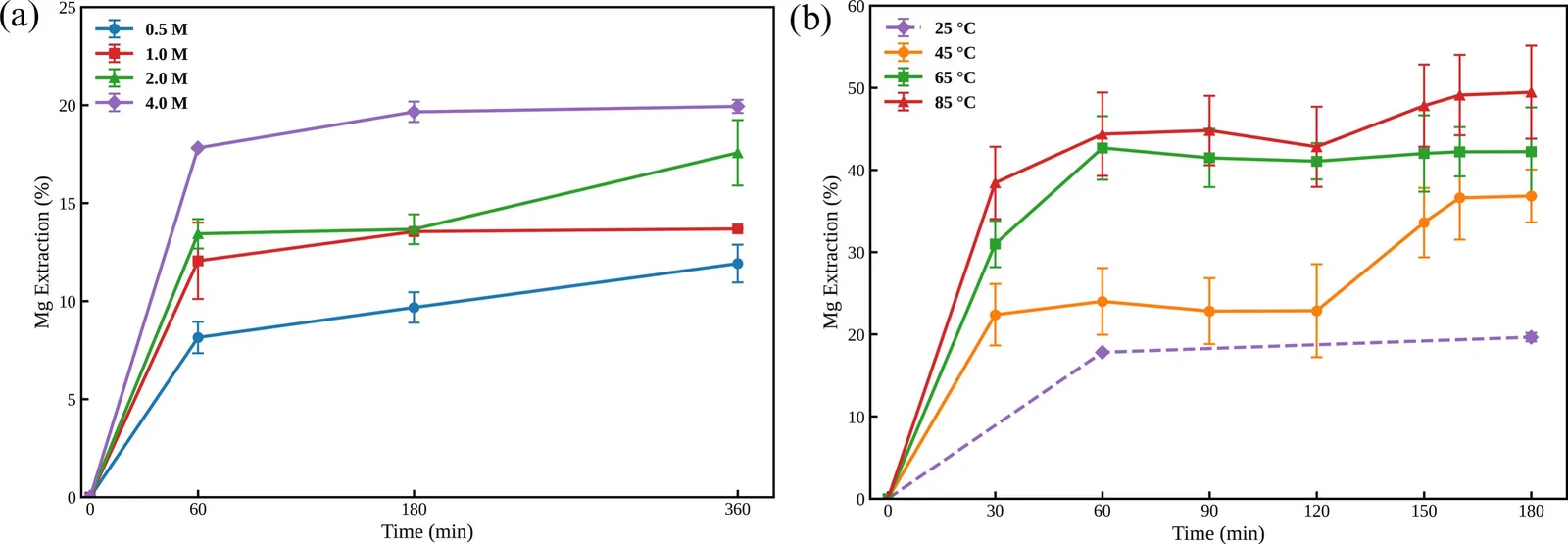
Baseline Mg^2+^ leaching performance under conventional conditions. (a) Mg^2+^ extraction as a function of leaching time during ambient-temperature (25 ± 2 °C) HNO_3_ leaching of raw lizardite at acid concentrations of 0.5, 1.0, 2.0, and 4.0 M. (b) Effect of temperature on Mg^2+^ extraction during conventional leaching at a fixed acid concentration of 4.0 M HNO_3_, showing profiles at 25, 45, 65, and 85 °C. The ambient-temperature baseline (25 °C) is shown as a dashed line for reference. Data are reported as mean ± SD (n = 3).

Mg^2+^ dissolution from the raw lizardite sample was first evaluated under conventional HNO_3_ leaching at ambient temperature (25 ± 2 °C) to establish a baseline leaching response. Fig. 2a presents Mg^2+^ extraction as a function of time (0–360 min) for acid concentrations between 0.5 and 4.0 M HNO_3_. All profiles exhibit biphasic behavior characterized by a rapid initial increase within the first 60 min followed by progressive deceleration toward a quasi-steady plateau. Increasing acid concentration systematically enhances both the initial dissolution rate and the final extraction level. After 360 min, Mg^2+^ extraction reaches 19.93% at 4.0 M compared with 11.91% at 0.5 M. At 4.0 M HNO_3_, the extraction increases sharply to 17.81% within 60 min but approaches a terminal plateau near 19.65% by 180 min, with negligible additional recovery upon extending leaching to 360 min. This stabilization near ∼20% represents the practical performance ceiling of ambient conventional leaching within the investigated time and operating window.

The observed plateau behavior is consistent with incongruent lizardite dissolution, in which proton attack preferentially removes Mg^2+^ from the brucite-like octahedral sheets while the SiO_4_ tetrahedral framework reorganizes at the reaction interface [77]. Progressive accumulation of a silica-rich alteration layer reduces effective pore accessibility and increases internal diffusion resistance [77], [78]. As the layer densifies, the system becomes increasingly influenced by product-layer transport resistance, where inward transport of H^+^ and outward diffusion of Mg^2+^ are progressively restricted. The stabilization at ∼20% extraction therefore reflects intrinsic transport limitation rather than reagent depletion [2], [79].

As shown in Fig. 2b, increasing temperature substantially accelerates the early-stage dissolution rate. At 60 min, Mg^2+^ extraction increases progressively from 22.31% at 45 °C to 44.35% at 85 °C. Final extraction at 180 min rises from 36.83% at 45 °C to 49.5% at 85 °C. However, despite faster extraction behaviour, all elevated-temperature profiles approach quasi-steady plateaus within 120–180 min. The progressive flattening of these curves indicates that elevated temperature primarily accelerates the approach to the transport-limited regime rather than fully overcoming silica-induced transport resistance [15]. Thus, although increasing the temperature enhances interfacial kinetics, it does not suppress formation of the silica-rich transport-restrictive alteration layer, leaving a substantial fraction of Mg^2+^ inaccessible within the remaining particle interior [19], [34], [80]. This highlights the necessity for intensification strategies, such as fluoride-mediated silicate destabilization or external physical intensification, capable of modifying this transport-restrictive alteration layer.

### 3.2 Chemical activation: fluoride-mediated silicate destabilization under thermal leaching conditions

**Fig. 3:**
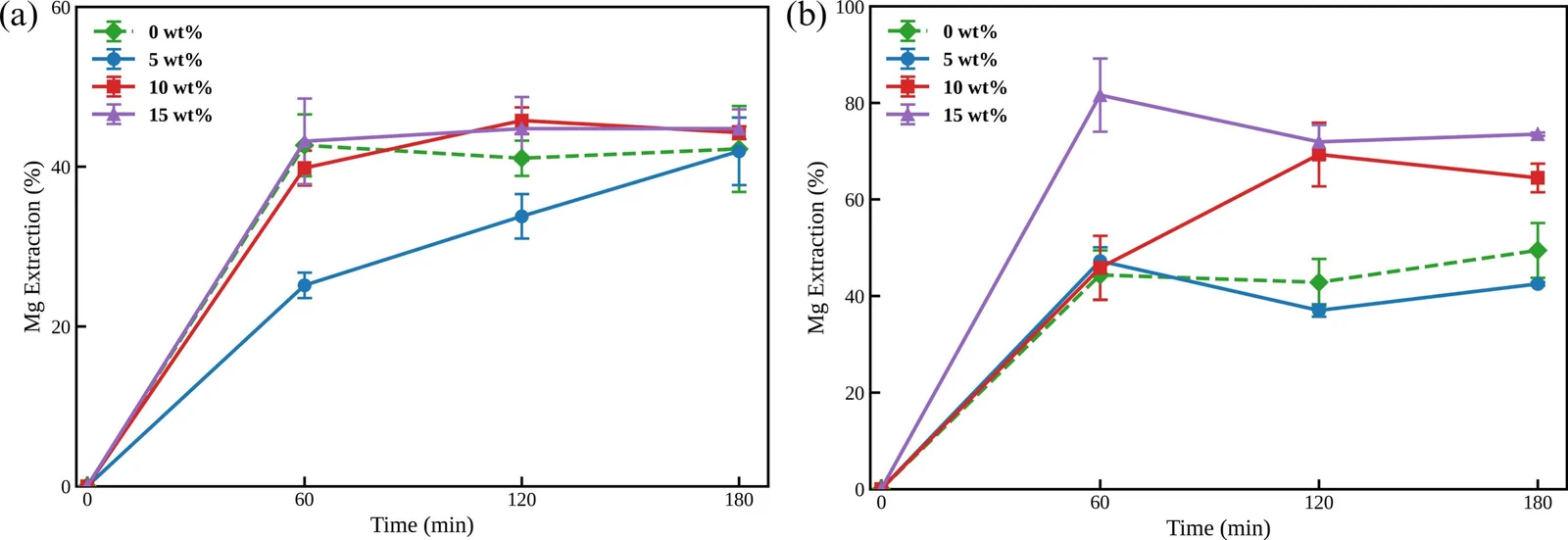
Effect of MgF_2_ addition on Mg^2+^ extraction during thermal leaching of lizardite (4.0 M HNO_3_). (a) Mg^2+^ extraction at 65 °C as a function of leaching time for MgF_2_ dosages of 0, 5, 10, and 15 wt%. Panel (a) is rescaled to 0–60% Mg^2+^ extraction to improve visualization of the modest and non-monotonic differences among MgF_2_ dosages at 65 °C. (b) Corresponding extraction behavior at 85 °C under identical chemical conditions. Data are reported as mean ± SD (n = 3). The fluoride-free system (0 wt% MgF_2_) is shown as a dashed line for reference.

The influence of MgF_2_ addition on Mg^2+^ extraction during thermal leaching (4.0 M HNO_3_) is presented in Fig. 3 for temperatures of 65 and 85 °C. Additional MgF_2_-assisted screening experiments were also conducted at 45 °C; these lower-temperature results are in the Fig. S4 and are used to support the temperature-series kinetic analysis discussed in Section 3.3.

At 65 °C (Fig. 3a), Mg^2+^ extraction increases progressively with time for all MgF_2_ dosages; however, the fluoride effect is modest and non-monotonic rather than uniformly beneficial. Relative to the MgF_2_-free baseline (0 wt%), the 5 wt% MgF_2_ condition gives lower extraction during the early and intermediate stages, while 10–15 wt% MgF_2_ produces only limited improvement, with terminal extraction values converging near ∼42–46% after 180 min. These results indicate that under moderate thermal conditions fluoride provides only partial activation.

A markedly different behavior is observed at 85 °C (Fig. 3b). Under these conditions, Mg^2+^ extraction becomes strongly dosage-dependent, although the enhancement remains threshold-like rather than linear. At 60 min, the 5 wt% MgF_2_ condition remains close to the MgF_2_-free baseline and shows no clear sustained enhancement. At longer residence times, it falls below or remains lower than the MgF_2_-free baseline. In contrast, higher MgF_2_ loading produces a much stronger response, particularly 15 wt% at short residence time and 10 wt% after longer residence, with 15 wt% MgF_2_ reaching ∼81.6% extraction at 60 min. Thus, at elevated temperature, MgF_2_ addition produces a strongly non-linear enhancement, indicating that fluoride-mediated silicate destabilization becomes substantially more effective under thermally intensified conditions.

The additional 45 °C screening results further support the temperature-dependent nature of MgF_2_ activation (Fig. S4). At this lower temperature, MgF_2_ addition produced only limited terminal improvement relative to the MgF_2_-free baseline, with 15 wt% MgF_2_ increasing Mg^2+^ extraction from 36.83% to 39.60% after 180 min. Although higher MgF_2_ loading produced some enhancement at intermediate leaching times, the final extraction values remained relatively close, indicating that fluoride-mediated silicate destabilization remains kinetically constrained under mild thermal conditions.

Under acidic conditions, MgF_2_ acts as a sparingly soluble source of aqueous fluoride-bearing species. Its dissolution can be represented as:

(14) $${\mathrm{MgF}}_{2}\left(s\right)⇌{\mathrm{Mg}}^{2+}\left(aq\right)+2F^{-}\left(aq\right)$$

Following dissolution, the aqueous fluoride distribution among F^−^, HF, and Si- or Al-associated fluoride species depends on solution acidity, temperature, ionic strength, and complexation reactions. The individual aqueous fluoride species were not separately quantified in the present study. The MgF_2_-derived fluoride-bearing species may subsequently interact with silica-rich interfacial domains formed during incongruent lizardite dissolution and promote fluoride–silicate complexation, including possible formation of soluble fluorosilicate species such as ${SiF}_{6}^{2-}$, as reported in previous fluoride-assisted silicate-leaching studies. Unlike CaF_2_ or NaF-based systems [39], MgF_2_ introduces Mg^2+^ as the counter-ion, thereby avoiding the addition of extraneous cations that could form inert secondary salts and complicate downstream liquor chemistry. Fluoride species can destabilize Si–O–Si linkages and may promote the formation of soluble fluorosilicate complexes, thereby suppressing densification and re-deposition of the silica-rich alteration layer [9], [42]. However, the effectiveness of this fluoride-silicate destabilization depends strongly on both temperature and effective fluoride availability at the reacting interface. At 65 °C, the system appears to remain in a sub-threshold activation regime, in which the effective dissolved fluoride available from low MgF_2_ dosages is insufficient to sustain destabilization of the silica-rich alteration layer.

Given the appreciable Al_2_O_3_ content (∼6.8 wt%) in the feed, limited fluoride species generated at 5 wt% MgF_2_ may be partially consumed by competitive Al–F complexation and localized Si–F interfacial reactions before sufficient silicate depolymerization can occur [15], [37], [42]. Competitive formation of stable fluoroaluminate complexes under acidic conditions is well established and represents a significant fluoride sink in Al-bearing leaching systems [37], [42], potentially limiting the effective fluoride activity available for silicate depolymerization at low MgF_2_ loadings [15]. This interpretation is further supported by the apparent Al co-leaching data presented in Table S1, which show substantial Al release under MgF_2_-containing conditions. Although ICP-MS does not directly resolve Al speciation, the elevated dissolved Al pool supports the likelihood that Al–F complexation can impose a meaningful competing demand on the available fluoride supply at low MgF_2_ loading, as discussed further in Section 3.7. Consequently, weak fluoride-induced perturbation of the reacting interface may introduce localized interfacial resistance that offsets any minor fluoride-induced benefit, leaving the system governed by silica-rich transport restriction.

At 85 °C, the combined effects of enhanced thermal activation and intensified fluoride–silicate interactions are consistent with delayed or reduced development of a rigid polymerized silica barrier. Elevated temperature increases both interfacial reaction rates and effective fluoride activity, which may improve access to Mg-bearing domains by delaying densification of the silica-rich alteration layer before diffusion resistance dominates [9], [15]. This interpretation is further supported by the increased pore accessibility, crystallinity loss, silicate-network modification, and disrupted surface morphology observed in the post-leaching residues, as discussed in Section 3.7. However, the 5 wt% MgF_2_ condition indicates that low fluoride availability still does not sustain effective alteration-layer destabilization at this temperature. The same sub-threshold mechanism appears to persist at 85 °C, but higher temperature likely accelerates both fluoride-silicate destabilization and competing interfacial reactions [15], [37]. When fluoride availability remains insufficient, these competing reactions may temporarily increase local mass-transfer resistance, explaining why the 5 wt% condition remains close to the baseline initially and then falls below or remains lower than it at longer residence times [15], [37], [42]. In contrast, the pronounced improvement observed at higher MgF_2_ loading, particularly 15 wt% at short residence time and 10 wt% after longer residence, reflects a transition from partial fluoride perturbation to extensive silicate-framework destabilization. This behavior is consistent with previously reported fluoride-assisted leaching systems, where temperature amplifies fluoride-induced lattice destabilization and interfacial reaction kinetics [9], [15], [39].

At 85 °C, Mg^2+^ extraction in MgF_2_-containing systems exhibits stabilization or partial decline at extended residence times, indicating that MgF_2_-assisted activation does not eliminate secondary interfacial constraints. This behavior is more plausibly associated with progressive interfacial constraints rather than acid depletion [15], [38]. One possible contributor is solution-phase saturation and subsequent re-deposition of byproducts. Rapid accumulation of Mg^2+^, dissolved silica species, and fluoride-bearing complexes may promote local supersaturation, potentially leading to precipitation of poorly crystalline secondary phases (e.g., fluoroaluminate or fluorosilicate species) [40], [42]. In parallel, silicon–fluoride complexes, such as ${SiF}_{6}^{2-}$, generated during dissolution may hydrolyze or reorganize under evolving solution conditions, forming amorphous Si–O–rich surface layers [42], [47]. Such deposits, if formed, could partially occlude reactive pathways, increase interfacial resistance, and contribute to transport limitations analogous to the silica-induced passivation observed under fluoride-free conditions [15].

Overall, the results demonstrate that MgF_2_ functions as a temperature-dependent chemical activator. Under moderate thermal conditions, fluoride only partially mitigates silica-rich transport restriction but does not eliminate internal diffusion constraints. At elevated temperature (85 °C), sufficiently high MgF_2_ loading provides effective fluoride activity to promote substantial silicate-framework destabilization and markedly higher Mg^2+^ extraction. The observed non-linear dosage response indicates that optimal performance is governed by a balance between fluoride-mediated silicate destabilization and secondary interfacial constraints, defining an operational window for chemical activation prior to the introduction of mechanical intensification strategies.

To confirm that the observed kinetic stabilization and quasi-plateau behavior are not attributable to acid depletion, a reagent-capacity analysis was performed. Under the baseline condition, 100 mL of 4.0 M HNO_3_ provides 0.40 mol of H^+^, whereas the Mg^2+^ inventory in the feed (10 g solid, 23.62 wt% Mg^2+^) corresponds to 0.097 mol Mg^2+^. Assuming approximately 2 mol H^+^ per mol Mg^2+^ released, consistent with proton-promoted dissolution of serpentine (e.g., Mg_3_Si_2_O_5_(OH)_4_ + 6H^+^ → 3 Mg^2+^ + 2SiO_2_ + 5H_2_O), approximately 0.19 mol H^+^ would be required for complete Mg^2+^ extraction. The system therefore operates with a ∼2.1-fold stoichiometric excess of acid relative to the theoretical requirement for complete Mg^2+^ dissolution, indicating that acid depletion is unlikely to be the primary cause of the observed kinetic stabilization. The decelerating behavior is therefore more plausibly attributed to interfacial transport resistance within the evolving alteration layer. Because MgF_2_ itself contains Mg^2+^, blank leaching tests (acid + MgF_2_ without mineral) were conducted under identical conditions, and the dissolved Mg^2+^ contribution from the additive was subtracted from all mixed-system results. Accordingly, all reported Mg^2+^ extraction values correspond exclusively to Mg^2+^ released from the lizardite matrix.

To quantitatively evaluate fluoride-induced enhancement beyond qualitative curve shifts, baseline-corrected extraction increments were examined. At 85 °C and 60 min, fluoride-free leaching yields 44.35% Mg^2+^ extraction, whereas 10 wt% and 15 wt% MgF_2_ yield 45.82% and 81.59%, respectively. Thus, the net fluoride-induced enhancement is minor at 10 wt% (ΔMg ≈ +1.47 percentage points) but substantial at 15 wt% (ΔMg ≈ +37.24 percentage points), confirming a strongly non-linear, threshold-type response characteristic of fluoride-mediated silicate-framework destabilization. In contrast, at 65 °C, fluoride-induced changes remain comparatively modest and non-monotonic, with terminal extraction values converging toward similar plateaus, indicating that fluoride activation alone only partially mitigates silica-rich transport restriction under moderate thermal conditions.

Collectively, these findings indicate that MgF_2_-mediated activation reflects a kinetic competition between silica polymerization (framework densification) and fluoride-driven depolymerization at the reacting interface. Optimal performance is achieved when lattice destabilization dominates over secondary phase formation and reprecipitation phenomena. This temperature-dependent balance defines the operational window for chemical activation prior to the introduction of mechanical intensification strategies.

### 3.3 Leaching kinetics and apparent activation energy

**Fig. 4:**
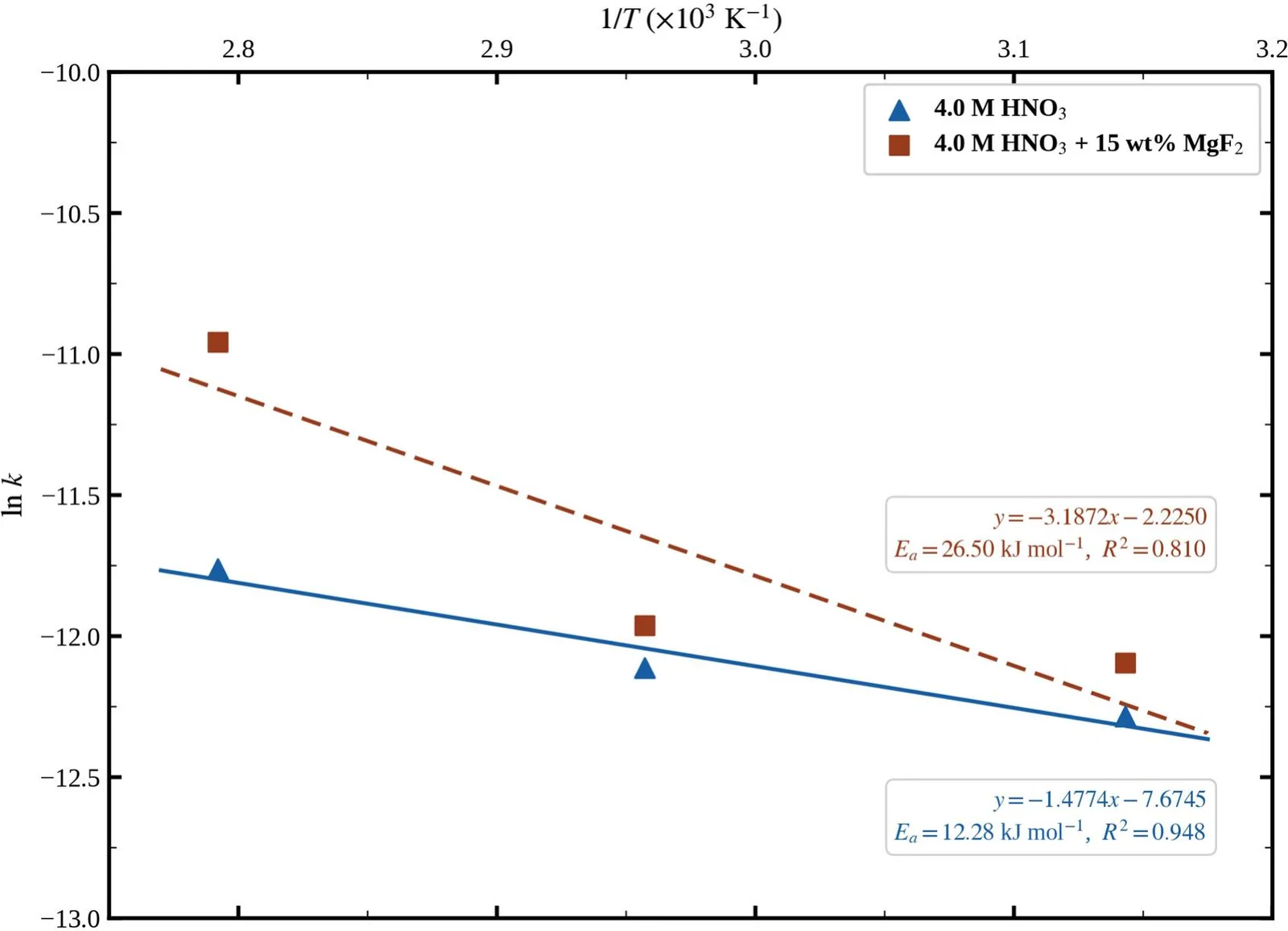
Arrhenius plots for Mg^2+^ extraction from lizardite-rich serpentine under conventional HNO_3_ leaching and MgF_2_-assisted leaching based on the product-layer diffusion model. Data points represent ln *k* values calculated from apparent rate constants obtained at 45, 65, and 85 °C; lines represent linear Arrhenius fits.

To provide quantitative support for the extraction trends in 3.1, 3.2, standard solid–liquid kinetic models were fitted to temperature-series Mg^2+^ extraction data for conventional thermal leaching (4.0 M HNO_3_, no MgF_2_) and MgF_2_-assisted thermal leaching (4.0 M HNO_3_ + 15 wt% MgF_2_) at 45, 65, and 85 °C. The 15 wt% MgF_2_ condition was selected because it provided the strongest short-residence activation response in dosage screening and corresponds to the TF-opt condition used for mechanistic and engineering comparison. Additional intermediate sampling points were included to improve time resolution; the expanded dataset is provided in Table S2, and the transformed product-layer diffusion fits are shown in Fig. S5. Five kinetic expressions were evaluated: product-layer diffusion control, surface chemical reaction control, liquid-film diffusion control, combined product-layer diffusion and chemical reaction control, and first-order pseudo-homogeneous kinetics. The product-layer diffusion model is discussed in detail because it gave the highest mean *R*^2^ across the temperature series for both systems and is mechanistically consistent with transport through an evolving silica-rich alteration/product layer during incongruent lizardite dissolution. The complete multi-model comparison, including apparent rate constants and *R*^2^ for all models and temperatures, is provided in Table S3 and Table S4; selected kinetic and Arrhenius parameters are summarized in Table 4, with the corresponding Arrhenius fits shown in Fig. 4.

**Table 4:** Selected kinetic and Arrhenius parameters based on the product-layer diffusion model.

System	*k*_45_ (s^−1^)	*k*_65_ (s^−1^)	*k*_85_ (s^−1^)	*R*^2^ @ 45 ℃	*R*^2^ @ 65 ℃	*R*^2^ @ 85 ℃	*E*_a_ (kJ mol^−1^)	ln *A*	*R*^2^_Arr_
Conventional thermal leaching	4.618 × 10^−6^	5.485 × 10^−6^	7.790 × 10^−6^	0.951	0.892	0.925	12.28	−7.67	0.948
MgF_2_-assisted thermal leaching	5.584 × 10^−6^	6.375 × 10^−6^	1.741 × 10^−5^	0.952	0.904	0.814	26.50	−2.22	0.810

Note: *R*^2^_Arr_ represents the coefficient of determination for the Arrhenius fit. At 85 °C, $k_{MgF_{2}}/k_{HNO_{3}}=2.24$.

For the conventional HNO_3_ system, the product-layer diffusion model gave the most consistent fit (*R*^2^ = 0.951, 0.892, 0.925 at 45, 65, 85 °C; respectively; highest mean *R*^2^ = 0.923 among the five models, Table S3). The apparent rate constant increased from 4.618 × 10^−6^ s^−1^ at 45 °C to 5.485 × 10^−6^ s^−1^ at 65 °C, and 7.790 × 10^−6^ s^−1^ at 85 °C, supporting thermal acceleration of Mg^2+^ release. However, the relatively modest increase across a 40 °C temperature interval indicates that increasing temperature alone does not overcome the transport resistance imposed by the evolving silica-rich alteration layer.

This behavior is mechanistically consistent with previous studies of serpentine dissolution [80], [81], [82]. During acid attack, Mg^2+^ is preferentially released from the brucite-like octahedral sheets, while the residual silicate framework progressively reorganizes at the reacting interface into a silica-rich product layer. This layer is neither static nor completely inert; it evolves through Mg^2+^ depletion, silica condensation, pore restructuring, and partial densification, progressively restricting inward H^+^ transport and outward Mg^2+^ diffusion. The quasi-plateau in extraction near ∼49–50% during conventional thermal leaching is therefore more consistent with increasing product-layer transport resistance than with acid depletion alone.

In the presence of 15 wt% MgF_2_, the product-layer diffusion model again provided the strongest overall fit (*R*^2^ = 0.952, 0.904, 0.814 at 45, 65, 85 °C, respectively; highest mean *R*^2^ = 0.890, Table S4). The apparent rate constant increased monotonically from 5.584 × 10^−6^ s^−1^ at 45 °C to 6.375 × 10^−6^ s^−1^ at 65 °C, and 1.741 × 10^−5^ s^−1^ at 85 °C, with the strongest increase occurring between 65 and 85 °C. MgF_2_ therefore increased the apparent rate constant at all three temperatures, with the largest enhancement observed at 85 °C, where *k* was 2.24-fold higher than in the MgF_2_-free system. The reduced product-layer fit at 85 °C${(R}^{2}$ = 0.814) indicates increasing deviation from ideal product-layer-diffusion behavior at the highest temperature and fluoride loading. This is consistent with the introduction of coupled temperature-dependent processes, including proton-promoted MgF_2_ dissolution, fluoride release under strongly acidic conditions, competitive Al–F complexation, fluoride-mediated modification of silicate linkages, and possible formation of fluorosilicate species [15], [37]. MgF_2_ should therefore not be interpreted as eliminating product-layer diffusion control; rather, it modifies the chemistry and permeability of the evolving solid–liquid interface, reducing effective transport restriction and improving accessibility of Mg-bearing domains.

Arrhenius analysis further clarifies the apparently counterintuitive combination of a higher *E*_a_ and larger rate constants in the MgF_2_-assisted system. The apparent activation energy increased from 12.28 kJ mol^−1^ for conventional thermal leaching to 26.50 kJ mol^−1^ for MgF_2_-assisted leaching, while ln *A* increased from −7.67 to −2.22. Because the Arrhenius relationship, $k=A\exp(-E_{a}/RT),$ depends jointly on *E*_a_ and *A*, a higher apparent activation energy does not necessarily imply a smaller rate constant. Over the investigated temperature range, the substantially larger fitted pre-exponential factor of the MgF_2_-assisted pathway compensates for the larger apparent activation-energy term, resulting in larger apparent *k* values at all three temperatures. These parameters characterize the overall heterogeneous dissolution–transport pathway and should therefore be interpreted as apparent pathway-level parameters rather than intrinsic elementary-reaction quantities or direct measures of specific microscopic processes. Moreover, the lower Arrhenius-fit coefficient for the MgF_2_-assisted system (*R*^2^_Arr_ = 0.810, compared with 0.948 for conventional leaching) indicates greater deviation from ideal Arrhenius behavior and warrants cautious interpretation.

Importantly, literature comparisons also demonstrate that the magnitude of apparent *E*_a_ alone should not be used as a unique diagnostic of the rate-controlling mechanism in serpentine dissolution. Teir et al. [81], for example, reported product-layer-diffusion-limited serpentinite dissolution despite apparent activation energies of 68, 70, and 74 kJ mol^−1^ in H_2_SO_4_, HCl, and HNO_3_, respectively. Wang and Maroto-Valer [82] reported a coupled chemical-reaction/product-layer-diffusion pathway with an apparent *E*_a_ of 40.9 kJ mol^−1^, while Peng et al. [15] reported an apparent *E*_a_ of 38.70 kJ mol^−1^ for fluorite-assisted serpentine leaching. Accordingly, the present values of 12.28 and 26.50 kJ mol^−1^ are interpreted together with the kinetic-model fits, rate-constant trends, and complementary residue characterization rather than through fixed activation-energy thresholds. Thus, the higher apparent *E*_a_ of the MgF_2_-assisted pathway should not, by itself, be interpreted as evidence of a transition to surface-reaction control.

To place these route-specific results within a common comparative framework, Table 5 summarizes the experimental basis, key comparative evidence, dominant mechanistic interpretation, and experimental boundary of the three principal activation routes.

**Table 5:** Unified comparison of the experimental basis, key comparative evidence, mechanistic interpretation, and experimental boundaries of the principal leaching pathways.

Pathway	Comparative basis	Key comparative evidence	Dominant mechanistic interpretation	Experimental boundary
Conventional thermal leaching, 4.0 M HNO_3_	45, 65, and 85 °C temperature series; product-layer diffusion best overall fit	*E*_a_ = 12.28 kJ mol^−1^; ln *A* = − 7.67; *k* increases with temperature	Thermal acceleration occurs, but transport through the evolving silica-rich alteration/product layer remains influential	Arrhenius-comparable
MgF_2_-assisted thermal leaching, 4.0 M HNO_3_ + 15 wt% MgF_2_	45, 65, and 85 °C temperature series; product-layer diffusion best overall fit	*E*_a_ = 26.50 kJ mol^−1^; ln *A* = − 2.22; higher *k* than the MgF_2_-free system at all temperatures (2.24-fold at 85 °C)	Fluoride modifies the evolving solid–liquid interface and reduces effective transport restriction; transport influence persists	Arrhenius-comparable
Ultrasound-assisted leaching, 4.0 M HNO_3_	Time-resolved leaching at controlled bulk T < 40 °C	47.62% Mg^2+^ extraction at 60 min versus 22.31% at 45 °C thermal leaching and 17.81% under the silent baseline; complementary PSD/SEM evidence	Cavitation-associated interfacial renewal, particle/surface modification, and transport intensification	No multi-temperature ultrasound series; *E*_a_ and *A* not determined

The two thermally evaluated pathways can be compared quantitatively using the common product-layer-diffusion/Arrhenius framework; the ultrasound-assisted pathway requires a different basis of comparison because no multi-temperature ultrasound dataset was generated.

The ultrasound-assisted pathway cannot be compared on the same Arrhenius basis because a multi-temperature ultrasound series was not performed. The ultrasound experiments were intentionally conducted within a controlled bulk-temperature window below 40 °C to minimize conventional bulk-temperature contributions rather than to generate temperature-dependent kinetic parameters. Consequently, ultrasound-specific Arrhenius parameters (*E*_a_ and *A*) are neither calculated nor inferred. Previous ultrasound-assisted leaching work has shown that enhancement under sonication does not necessarily require a decrease in apparent activation energy and, in at least one leaching system, the increase in the apparent rate constant was attributed mainly to an increase in the pre-exponential factor [83]. This literature precedent is used only as contextual support and is not used to assign unmeasured Arrhenius parameters to the present ultrasound-assisted system.

In place of an Arrhenius comparison, the ultrasound-assisted pathway is evaluated using matched-time extraction behavior together with self-generated physical evidence. At 60 min, ultrasound-assisted leaching achieved 47.62% Mg^2+^ extraction at <40 °C, compared with 22.31% during conventional thermal leaching at 45 °C and 17.81% under the corresponding silent baseline. Thus, under the tested conditions, the magnitude of the observed ultrasound-assisted enhancement cannot be explained by conventional bulk-temperature elevation alone. This interpretation is further supported by the paired residue comparisons presented in Section 3.7.6. At near-equivalent Mg^2+^ extraction, U-opt exhibited approximately 35% higher *D*_edge_ and 34% higher *D*_branch_ than T-opt, together with greater lamellar-edge exposure and surface opening. Because the two residues reached similar Mg^2+^ extraction extents, these morphological differences support a distinct surface-modification response under ultrasound rather than simply reflecting a greater extent of dissolution.

The early-stage 30 min validation series provides complementary evidence. U30 exhibited lower D50 and D90 values than T30 (32.9 versus 37.1 µm and 154 versus 165 µm, respectively), together with higher semi-quantitative SEM/ImageJ edge-density and branch-point-density indices. This comparison is matched in residence time rather than temperature because T30 was generated at 85 °C whereas U30 was maintained below 40 °C; it is therefore interpreted as a comparison between the two process routes rather than as a strictly temperature-matched kinetic experiment. Nevertheless, the combined PSD and SEM/ImageJ trends are consistent with stronger particle/surface modification under ultrasound. These measurements do not constitute direct determination of cavitation intensity, alteration-layer thickness, or complete separation of acoustic and thermal contributions; rather, they provide self-generated experimental evidence consistent with a cavitation-associated contribution involving interfacial renewal, particle/surface modification, and improved transport accessibility.

The coupled ultrasound–MgF_2_ pathway is not treated as a fourth independent kinetic regime because it combines the fluoride-mediated interface modification and ultrasound-assisted physical intensification defined above and was not evaluated through an independent multi-temperature series. Its synergistic process-level response is therefore examined separately in Section 3.5.

Taken together, the three pathways should not be interpreted as three mutually exclusive elementary rate-controlling mechanisms. Instead, they represent distinct modes of modifying a common transport-influenced dissolution system. Conventional thermal activation accelerates dissolution while silica-rich product-layer transport resistance remains influential; MgF_2_-assisted thermal activation chemically modifies the evolving solid–liquid interface and reduces its effective transport restriction; and ultrasound-assisted leaching provides cavitation-associated physical interfacial renewal and transport intensification within a controlled low-bulk-temperature window. This unified framework therefore establishes the mechanistic boundaries of the three activation routes without implying an experimentally demonstrated transition among three discrete elementary rate-controlling mechanisms.

### 3.4 Physical intensification: effect of ultrasonic irradiation on Mg^2+^ leaching

The influence of ultrasonic irradiation on Mg^2+^ extraction was evaluated at acid concentrations between 0.5 and 4.0 M under controlled acoustic irradiation (20 kHz, 40% amplitude), with mechanical stirring maintained at 200 rpm. During all experiments, bulk slurry temperature was kept below 40 °C to minimize conventional bulk-temperature contributions and evaluate ultrasound-assisted intensification within a controlled low-temperature window.

**Fig. 5:**
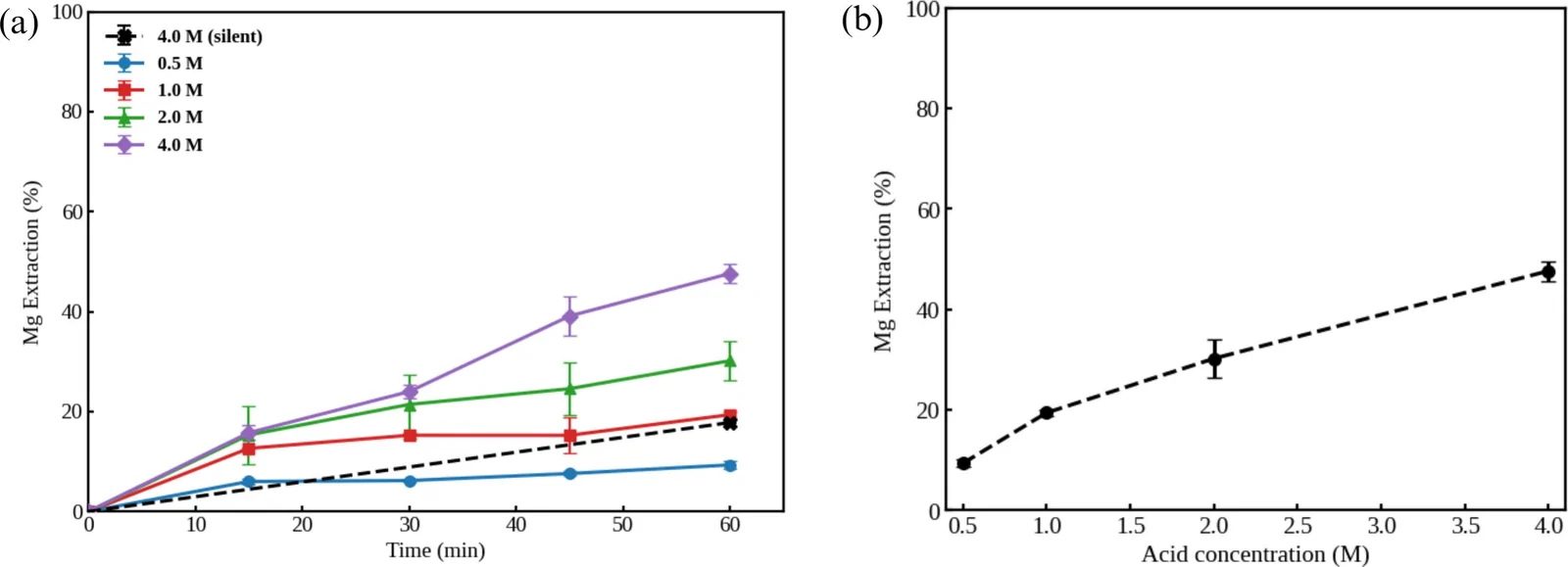
Ultrasound-assisted Mg^2+^ extraction from raw lizardite under HNO_3_ leaching conditions. (a) Mg^2+^ extraction as a function of leaching time during ultrasonic irradiation (20 kHz, 40% amplitude) at different concentrations of 0.5, 1.0, 2.0, and 4.0 M. The corresponding 4.0 M HNO_3_ silent-leaching baseline is included as a dashed black reference line based on the experimentally measured 0 and 60 min values. (b) Dependence of Mg^2+^ extraction on acid concentration at a fixed leaching time of 60 min under ultrasound. In all experiments, bulk slurry temperature was maintained below 40 °C to minimize conventional bulk-temperature contributions. Data are reported as mean ± SD (n = 3).

Fig. 5a shows Mg^2+^ extraction as a function of time (0–60 min) under different acid concentrations. For all concentrations, extraction increases monotonically with time, with the highest values observed at 60 min. Increasing acid concentration enhances extraction at each time point. After 60 min, Mg^2+^ extraction reaches 9.33%, 19.38%, 30.17%, and 47.62% for 0.5, 1.0, 2.0, and 4.0 M HNO_3_, respectively. Fig. 5b presents Mg^2+^ extraction at 60 min as a function of acid concentration, illustrating a non-linear increase in recovery with increasing acidity. The strong dependence on acid concentration confirms that while ultrasound accelerates extraction behavior, proton availability remains a primary chemical driving factor [37]. This trend is consistent with ultrasound-assisted enhancement of interfacial transport and surface renewal without removing the underlying acid dependence of lizardite dissolution [84].

A key comparison emerges when these results are evaluated against both silent ambient leaching and conventional thermal leaching. Under ultrasound at <40 °C, Mg^2+^ extraction reaches 47.62% within 60 min, compared with 17.81% for the corresponding silent leaching baseline at 4.0 M HNO_3_ after 60 min without ultrasonic irradiation. This corresponds to an approximately 2.7-fold enhancement relative to silent leaching. Moreover, conventional leaching at 85 °C yields 49.45% after 180 min. Thus, ultrasonic irradiation achieves approximately 96% of the high-temperature extraction extent while reducing bulk temperature by ∼45 °C and shortening residence time by a factor of three. This indicates that ultrasound-assisted interfacial intensification can partially compensate for conventional bulk thermal input under the investigated conditions [84].

The observed ultrasound-assisted enhancement is consistent with the established effects of acoustic cavitation in heterogeneous solid–liquid systems. The propagation of high-frequency pressure waves generates cavitation bubbles that undergo rapid growth and collapse [85], [86]. Near solid surfaces, asymmetric collapse produces localized microjets and shockwaves that perturb silica-rich surface films and alteration layers, disrupt boundary layers, and continuously renew the solid–liquid interface [87], [88]. These effects increase the effective external mass-transfer coefficient and facilitate penetration of acid into microcracks and shallow pores [84], [87], [89]. However, cavitation primarily addresses external transport resistance and silica-rich surface-film/alteration-layer resistance rather than eliminating intrinsic lattice-controlled diffusion constraints within the particle interior [90].

Despite the substantial acceleration in early-stage kinetics, extraction under ultrasound approaches ∼47.62% at 60 min using 4.0 M HNO_3_, with diminishing incremental gains at longer residence times. The reduced slope between 45 and 60 min compared to 30 and 45 min suggests the onset of transport limitation associated with the evolving alteration layer. Therefore, although ultrasound-assisted behavior is consistent with delayed development of silica-rich transport resistance and enhanced interfacial renewal under low-severity conditions, it does not fully overcome internal diffusion resistance. Overall, ultrasound represents an effective physical intensification strategy capable of achieving near-thermal performance under reduced temperature and controlled acoustic conditions. However, the persistence of a sub-50% extraction ceiling indicates that physical activation alone is insufficient to fully access the Mg^2+^ inventory. This limitation motivates coupling ultrasound-assisted physical intensification with fluoride-mediated silicate destabilization, as examined in the subsequent section.

Although the ultrasound-assisted experiments in this study were conducted within a controlled bulk-temperature window (<40 °C) to minimize conventional bulk-temperature contributions, acoustic energy dissipation inevitably generates heat during sonication. From an industrial scale-up and process-intensification perspective, allowing a controlled increase in bulk temperature could reduce active cooling requirements and may shorten the residence time required to reach a target Mg^2+^ extraction. Cavitation behavior is itself temperature-dependent [91], [92]; however, because cavitation activity was not directly measured as a function of temperature in the present study, the influence of increasing bulk temperature on cavitation intensity cannot be established here. Evaporation losses and ultrasonic-probe operating constraints also require consideration. Therefore, the temperature-controlled ultrasound experiments should be interpreted as a controlled low-temperature baseline for evaluating ultrasound-assisted enhancement rather than as complete separation of acoustic and thermal contributions. Future process optimization should therefore evaluate controlled self-heating together with cavitation activity, allowable bulk-temperature rise, residence time, and energy input.

### 3.5 Synergistic intensification: coupling ultrasound and fluoride activation

**Fig. 6:**
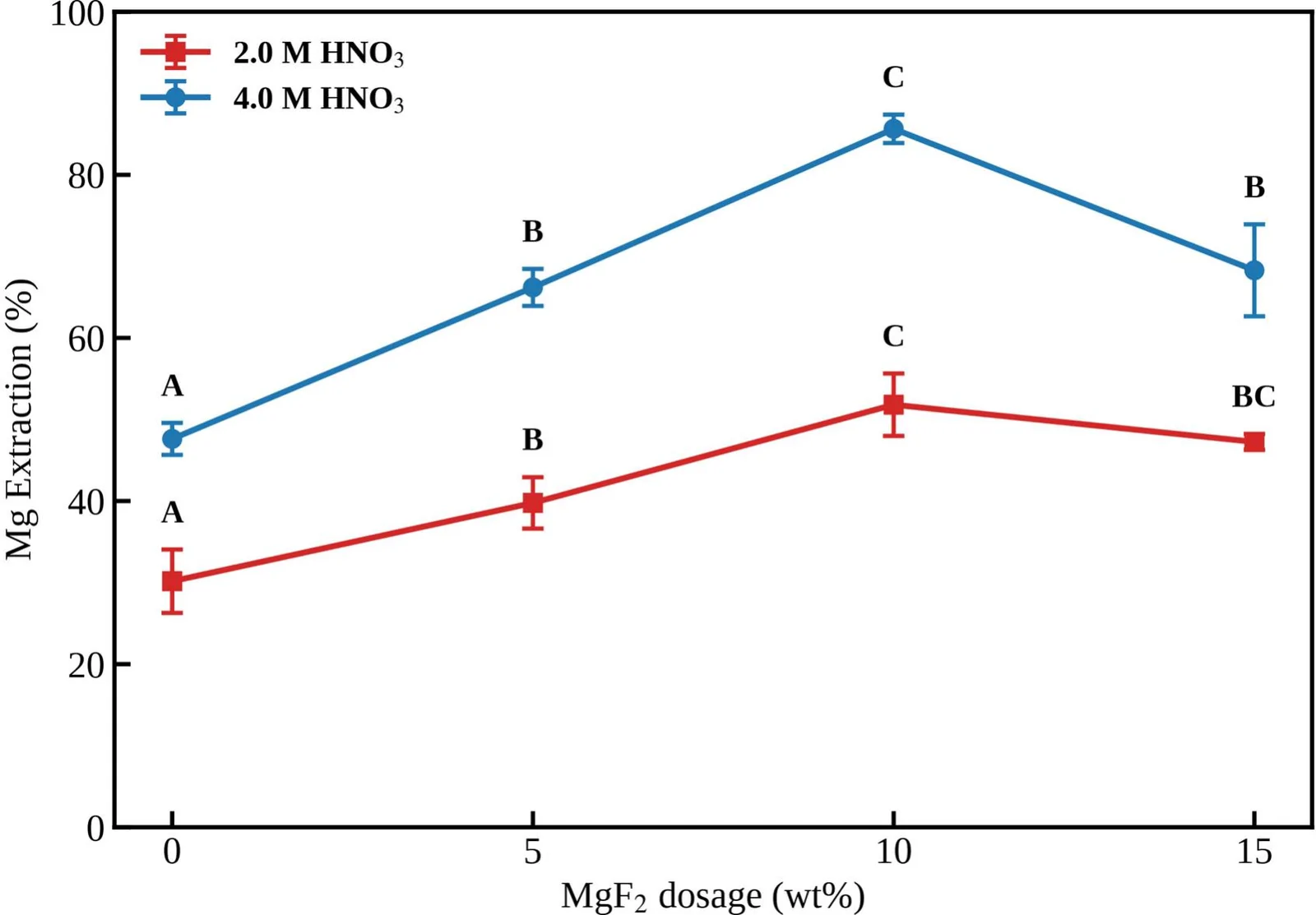
Effect of MgF_2_ dosage on Mg^2+^ extraction during ultrasound-assisted leaching at a fixed leaching time of 60 min. Mg^2+^extraction profiles are shown for 2.0 M and 4.0 M HNO_3_ under ultrasonic irradiation (20 kHz, 40% amplitude), with bulk slurry temperature maintained below 40 °C. Data are presented as mean ± SD (n = 3). Different letters indicate statistically significant differences according to Tukey’s HSD test (*p* < 0.05).

To directly compare activation effects under identical hydrodynamic conditions, a fixed leaching time of 60 min was selected for all ultrasound–fluoride experiments. Fig. 6 presents Mg^2+^ extraction as a function of MgF_2_ dosage under ultrasonic irradiation (20 kHz, 40% amplitude) at acid concentrations of 2.0 and 4.0 M.

At 2.0 M HNO_3_, ultrasound alone yields 30.17% extraction after 60 min. The addition of MgF_2_ increases extraction to 39.8% at 5 wt% and to 51.81% at 10 wt%, followed by a decline to 47.25% at 15 wt%. Thus, under reduced acidity, fluoride addition enhances recovery but exhibits a clear optimum near 10 wt%, beyond which performance deteriorates. A markedly stronger response is observed at 4.0 M HNO_3_. Under ultrasound without fluoride, Mg^2+^ extraction reaches 47.62% at 60 min. With 5 wt% MgF_2_, extraction increases to 66.19% and further rises to 85.64% at 10 wt%. Increasing dosage to 15 wt% reduces extraction to 68.29%. The enhancement at 10 wt% represents a 38.02-percentage-point increase relative to ultrasound alone.

Importantly, the coupled system at 4.0 M achieves 85.64% extraction within 60 min at < 40 °C, exceeding the maximum value obtained under thermal–fluoride leaching at 85 °C (81.6% at 60 min) while using a lower MgF_2_ dosage (10 wt% vs. 15 wt%). This indicates that the combined activation pathway yields performance gains beyond those attainable by either chemical or physical intensification independently. The observed non-monotonic behavior at higher MgF_2_ loading suggests that system performance is governed by a balance between fluoride-mediated silicate destabilization, cavitation-enhanced mass transfer, and secondary interfacial constraints [15], [38]. At moderate dosages, fluoride promotes destabilization of Si–O–Si linkages and suppresses densification of the silica-rich alteration layer [15], [42]. Simultaneously, ultrasonic cavitation enhances interfacial renewal, disrupts boundary layers, and improves convective transport of H^+^ and fluoride species into near-surface and internal pore domains. The combination may improve access to otherwise less accessible Mg-bearing domains before diffusion resistance becomes dominant [88], [93].

The decline in extraction observed at 15 wt% MgF_2_ for both acid concentrations may be attributed to rapid localized supersaturation under high fluoride flux. Under intense cavitation and elevated fluoride loading, the rate of Mg^2+^, dissolved silicate species, and fluoride release can exceed local solubility limits, promoting transient precipitation of fluoride-bearing or fluorosilicate phases or formation of amorphous surface films that partially obstruct reactive pathways [38], [40]. The presence of an optimum dosage therefore defines an operational window in which chemical depolymerization and cavitation-enhanced transport are effectively balanced.

Overall, the coupled ultrasound–fluoride system shifts leaching behavior from a transport-limited regime toward a transport-enabled extraction regime under reduced thermal severity. The substantial increase in attainable extraction, achieved at lower temperature and moderate additive loading, demonstrates that the synergy arises from simultaneous mitigation of silica-rich transport restriction and mass-transfer resistance rather than from increased bulk heating alone. To quantitatively benchmark the engineering significance of this intensification beyond extraction percentage, the different activation pathways are evaluated in terms of STY in the following section.

### 3.6 Process intensification benchmarking

Following the comparative evaluation of different leaching pathways, a quantitative performance assessment was conducted to translate extraction improvements into engineering productivity metrics. The investigated leaching routes were benchmarked using STY as a unified productivity descriptor [76], [94]. Unlike extraction percentage alone, STY normalizes conversion with respect to processing time and working volume, enabling direct comparison between leaching strategies operating under different temperatures, additive loadings, acoustic conditions, and residence times. This approach allows discrimination between apparent conversion enhancement and genuine process intensification. In the present study, STY is defined as a batch volumetric productivity metric (product generated per unit liquid volume per unit time) and does not imply continuous reactor operation.

The five representative operating conditions are designated as A-opt (ambient conventional baseline), T-opt (thermal leaching), U-opt (ultrasound-assisted leaching), TF-opt (thermal + MgF_2_ additive), and UF-opt (ultrasound + MgF_2_ additive), with the corresponding parameters summarized in Table 6. For each leaching class, the selected “opt” condition corresponds to the experimentally identified performance plateau or practical operational window for that pathway, and the associated residence time reflects the characteristic extraction timescale of that route.

**Table 6:** Summary of textural properties and pore-architecture evolution of raw and leached samples under different leaching pathways.

Sample	Temperature (°C)	Time (min)	Mg^2+^ extraction (%)	BET surface area (m^2^ g^−1^)	Total pore volume (PV) (cm^3^ g^−1^)	*PV < 2 nm (%)	PV 2–10 nm (%)	PV > 10 nm (%)	Dominant pore regime	Transport implication
RS	–	–	–	15.7	0.035	3.6	26.3	70.1	Wide mesopores (>∼10 nm) fraction at low total PV	Low accessible internal pore capacity
A-opt	∼25	180	19.7	34.9	0.064	9.6	44.2	46.2	Small mesopores	Limited pore development; persistent transport constraints
T-opt	85	180	49.5	301.9	0.279	13.7	52.4	33.9	Micro–mesopore dominant (<10 nm)	Transport-restricted micro–mesoporous architecture; silica-rich transport restriction
TF-opt	85	60	81.6	106.3	0.287	0.6	32.1	67.3	Wide mesopores (>10 nm)	Transport-accessible wide-mesoporous architecture
U-opt	<40	60	47.6	164.0	0.193	9.1	41.9	49.0	Mixed mesopores	Ultrasound-associated pore/surface opening and enhanced accessibility
UF-opt	<40	60	85.6	116.3	0.318	0.8	28.3	70.9	Hierarchical wide mesopores (>∼10 nm; ∼71%)	Lowest inferred internal transport restriction
*Note. Pore volume fractions are expressed as percentages of the total pore volume and were determined from NL-DFT analysis of the N_2_ adsorption branch using the equilibrium NL-DFT model implemented in ASiQwin.										

**Fig. 7:**
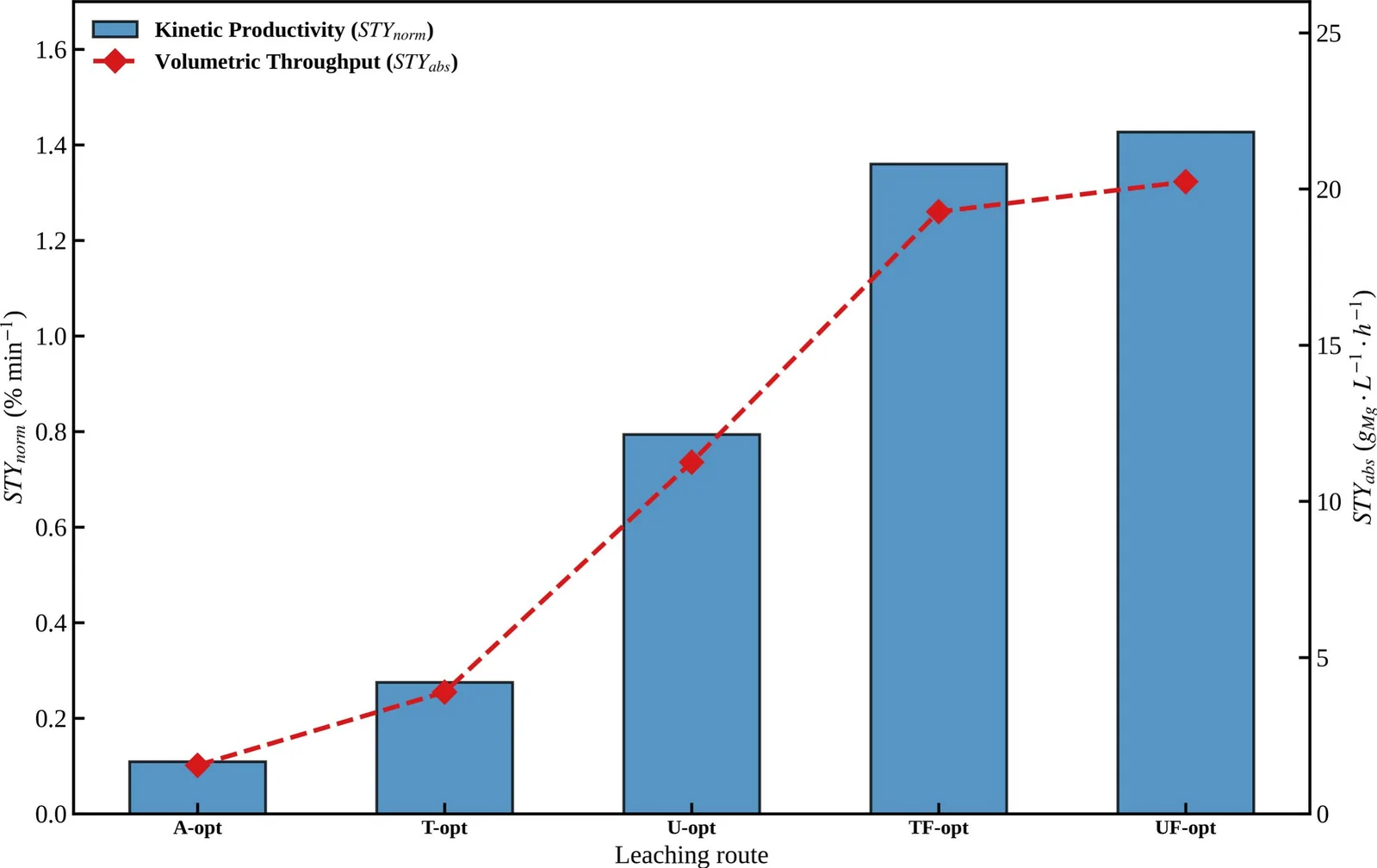
Process intensification benchmarking of leaching routes using normalized and absolute STY. Bars represent normalized kinetic productivity (${\mathrm{STY}}_{\mathrm{norm}}$, % min^−1^), while diamonds denote absolute volumetric throughput (${\mathrm{STY}}_{\mathrm{abs}}$, g_(__Mg__)_ L^−1^h^−1^). The systematic increase across the leaching hierarchy (A-opt → T-opt → U-opt → TF-opt → UF-opt) highlights the progressive decoupling of productivity from bulk process severity, with the UF-opt system achieving the highest throughput under low-temperature operating conditions.

Using the normalized productivity definition provided in Section 2.6, the five representative activation pathways were compared in terms of average extraction productivity over their selected residence times. This metric does not imply constant reaction-rate behavior. As shown in Fig. 7, A-opt represents the diffusion-limited baseline (${\mathrm{STY}}_{\mathrm{norm}}$ = 0.109% min^−1^), consistent with rapid silica-rich transport restriction and severe internal transport constraints [95], [96]. T-opt increases productivity to 0.275% min^−1^ but remains transport-limited at extended residence times due to the formation of a tortuous, silica-rich micro–mesoporous residue [17], [97]. In contrast, U-opt achieves a comparable extraction extent within 60 min, increasing productivity to 0.794% min^−1^. This demonstrates that cavitation-assisted interfacial intensification can partially substitute for conventional bulk thermal input under the investigated conditions [16], [48]. MgF_2_-mediated silicate destabilization further amplifies productivity. TF-opt reaches${\mathrm{STY}}_{\mathrm{norm}}$ = 1.360% min^−1^, while UF-opt yields the highest normalized productivity observed, 1.427% min^−1^, at <40 °C and with a reduced additive loading, 10 wt% MgF_2_ compared with 15 wt% for TF-opt.

The absolute STY defined in Section 2.6 was additionally used to compare volumetric throughput across the activation pathways. As illustrated in Fig. 7, ${\mathrm{STY}}_{\mathrm{abs}}$ increases systematically across the leaching hierarchy from A-opt to UF-opt. Under UF-opt conditions, an absolute productivity of 20.23 g_(__Mg__)_ L^−1^h^−1^ was achieved, representing the highest volumetric throughput observed while maintaining the mildest bulk operating conditions.

Taken together, the benchmarking results demonstrate that the observed performance gains arise primarily from engineered transport efficiency rather than from bulk thermal severity alone. The systematic increase in STY across the leaching hierarchy supports the interpretation that process intensification is governed by progressive mitigation of silica-induced transport resistance. The structural and textural origins of these productivity gains are examined in detail in the subsequent pore-architecture and surface-fractal characterization section.

### 3.7 Analytical characterization and mechanistic interpretation

To examine the mechanistic basis of the enhanced Mg^2+^ extraction under thermal, ultrasound-assisted, and MgF_2_-assisted leaching, solid residues were systematically characterized using N_2_ physisorption, BET/NL-DFT pore analysis, FHH fractal analysis, FT-IR spectroscopy, XRD, SEM imaging, PSD analysis, and SEM/ImageJ quantification. Rather than treating these techniques independently, the results are interpreted within a unified, transport-centered framework that links pore-architecture evolution, pore-size redistribution, accessible pore volume, pore-surface roughness, particle-size modification, and surface-morphology evolution to the observed leaching performance. Together, these datasets provide convergent support for the proposed mechanistic interpretation by linking residue evolution to transport-relevant pore accessibility, particle/surface modification, and alteration-layer evolution associated with Mg^2+^ release.

#### 3.7.1 Textural evolution and pore architecture (BET and NL-DFT)

**Fig. 8:**
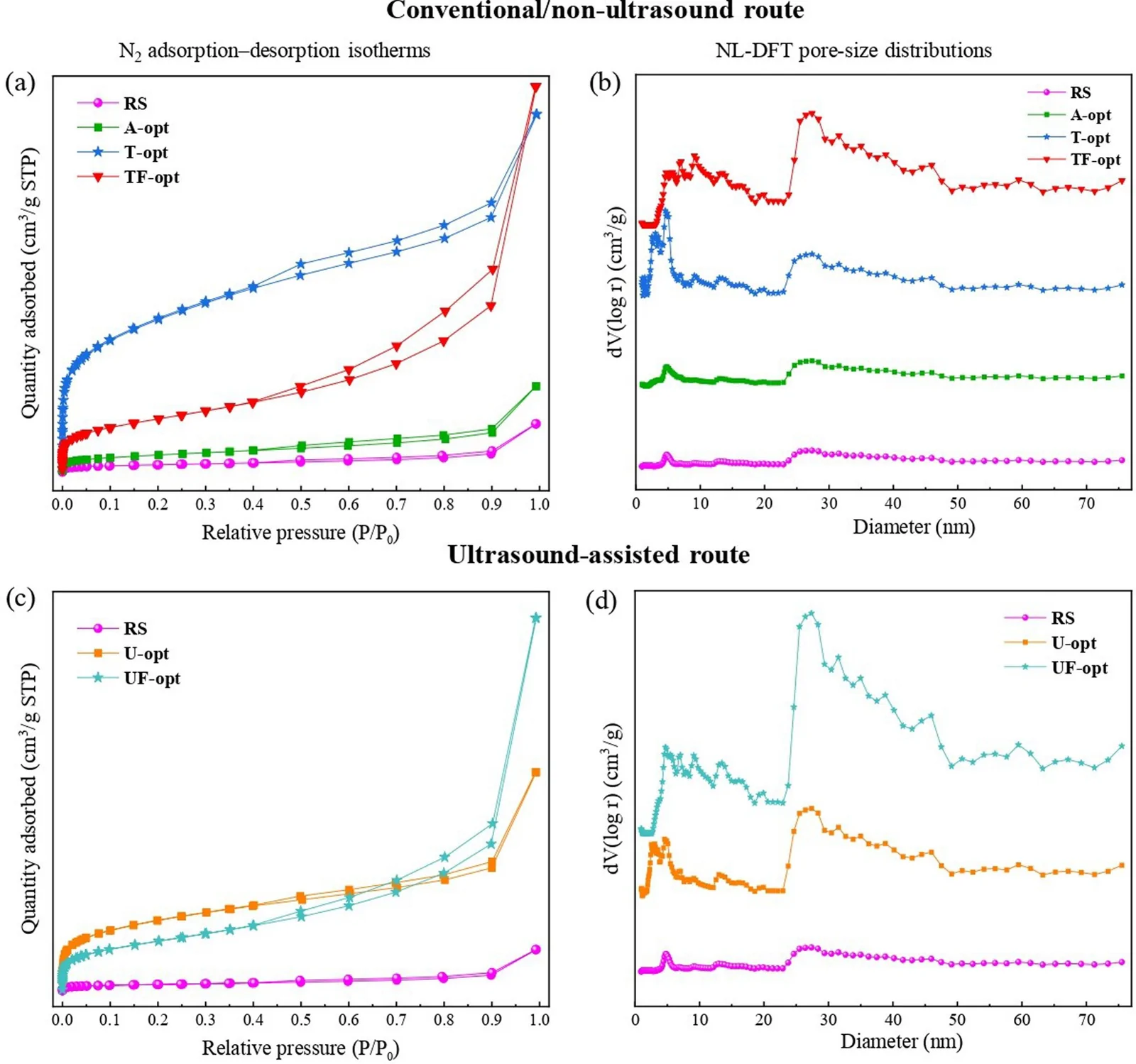
N_2_ physisorption and pore architecture evolution of lizardite residues under different leaching pathways. (a) N_2_ adsorption–desorption isotherms and (b) corresponding pore-size distributions for the conventional (non-ultrasound) leaching route (RS, A-opt, T-opt, TF-opt). (c) N_2_ adsorption–desorption isotherms and (d) corresponding pore-size distributions for the ultrasound-assisted leaching route (RS, U-opt, UF-opt). The residues generally exhibit Type IV(a)-like isotherms with H_3_-type hysteresis, consistent with slit-shaped porosity in layered phyllosilicate-derived structures.

The textural evolution of lizardite residues under different leaching pathways was quantified using N_2_ physisorption, combining BET surface area with NL-DFT-derived pore-size distributions and total pore volume (Table 6). While BET provides a measure of accessible surface area, NL-DFT analysis resolves the redistribution of pore volume across micro- and mesopore regimes and the emergence of large mesopore/macropore-like voids within the measurable range, thereby enabling evaluation of transport-relevant pore accessibility rather than surface area alone. The corresponding N_2_ adsorption–desorption isotherms, as shown in Fig. 8, provide qualitative support for the quantitative BET and pore-size distributions trends, allowing direct comparison of how thermal, MgF_2_-assisted, and ultrasound-assisted treatments modify pore regime dominance and internal mass-transfer accessibility.

According to Table 6, raw sample (RS) exhibits a low BET surface area (15.7 m^2^ g^−1^) and total pore volume (0.035 cm^3^ g^−1^), reflecting its dense phyllosilicate structure with limited internal accessibility. Although the fractional pore-volume distribution is weighted toward larger pores (PV > 10 nm), the absolute pore volume remains low, so the internal network contributes little transport-effective capacity and may contribute to diffusion-restrictive behavior during leaching [98]. Correspondingly, RS exhibits low N_2_ uptake across the measured P/P_0_ range with a modest H_3_-type hysteresis, consistent with slit-shaped voids associated with layered phyllosilicates and limited accessible internal porosity (Fig. 8a and b) [99]. After ambient leaching (A-opt), the BET surface area increases modestly to 34.9 m^2^ g^−1^ and the pore volume to 0.064 cm^3^ g^−1^, accompanied by the development of small mesopores. The corresponding increase in N_2_ uptake at intermediate relative pressures is consistent with surface-limited dissolution and limited development of accessible mesoporosity. However, the accessible mesopore network remains underdeveloped, so diffusion constraints persist, and Mg^2+^ extraction is capped at 19.7%, supporting the interpretation that ambient leaching remains transport-limited [95], [96].

T-opt induces a dramatic textural transformation, with the BET surface area increasing sharply to ∼302 m^2^ g^−1^. This increase is accompanied by extensive development of micropores and small mesopores (13.7% <2 nm and 52.4% between 2–10 nm), as evidenced by pronounced N_2_ uptake at low relative pressures and a substantially broadened H_3_ hysteresis loop (Fig. 8a and b) [99]. Such isotherm features are consistent with extensive micropore filling and the development of a highly polymerized silica-rich alteration phase with abundant micro–small-mesoporous domains.

Although thermal leaching generates a very large internal surface area, this surface is predominantly confined within narrow, transport-restrictive pores, which impose severe steric hindrance and elevate mass-transfer resistance [17], [97]. Under these conditions, solute transport is increasingly dominated by wall interactions rather than bulk diffusion, leading to reduced effective diffusivity of hydrated H^+^ and Mg^2+^ species [15]. Consequently, despite the nearly order-of-magnitude increase in surface area, Mg^2+^ extraction reaches only ∼49.5%, revealing a clear high-surface-area paradox, i.e., a decoupling between apparent surface area and transport-accessible pore architecture [81], [100].

In contrast, TF-opt follows a fundamentally different pore-evolution pathway. Although the BET surface area decreases to ∼106 m^2^ g^−1^, reflecting the loss of micropore-derived surface area through pore widening, the total pore volume remains high, ∼0.287 cm^3^ g^−1^, and the pore-size distributions shift decisively toward wider mesopores (and macropore-like voids within the measurable range). This behavior is consistent with the geometric volume-to-surface relationship governing porous solids, where the characteristic pore size (hydraulic radius) scales with the ratio of pore volume to specific surface area. Consequently, pore widening reduces BET surface area even when total pore volume remains constant or increases, consistent with the surface–volume scaling of porous solids [60]. Both the pore-size distributions and the adsorption–desorption isotherm (Fig. 8a and b) exhibit a pronounced uptake at high relative pressures (P/P_0_ ≈ 0.8–1.0), reflecting the dominance of enlarged, more open mesoporous domains rather than densified microporosity.

The fluoride-bearing species released from MgF_2_ under acidic conditions facilitate modification of the silica-rich framework by promoting fluoride–silicate interactions, widening narrow silica-rich bottlenecks, and limiting formation of a dense, transport-restrictive silica-rich alteration layer [9], [15], [40]. As a result, pore accessibility increases noticeably, consistent with lower internal mass-transfer restriction, and Mg^2+^ extraction rises to ∼81.6% despite the lower BET surface area. This inversion of the surface-area–extraction relationship strongly suggests that transport-efficient pore architecture, rather than absolute surface area, governs leaching efficiency.

A similar but more pronounced trend is observed under ultrasound-assisted conditions. U-opt increases the BET surface area to ∼164 m^2^ g^−1^ and pore volume to ∼0.193 cm^3^ g^−1^, consistent with cavitation-associated particle/surface modification, pore opening, and lamellar edge exfoliation, raising Mg^2+^ extraction to ∼47.6% [16], [48]. When MgF_2_ is introduced (UF-opt; 10 wt% MgF_2_), the BET surface area decreases (∼116 m^2^ g^−1^), while the total pore volume increases to the highest observed value (∼0.318 cm^3^ g^−1^), dominated by wide mesopores (and macropore-like voids within the measurable range), as shown in Table 6. The corresponding isotherm exhibits the broadest hysteresis loop and the steepest adsorption tail at high relative pressure, reflecting the development of a more open, hierarchical mesoporous architecture (Fig. 8c and d). The similarity between TF-opt and UF-opt suggests that once silica bottlenecks are sufficiently disrupted, residues evolve toward a more transport-efficient wide-mesoporous architecture.

Collectively, these results support the interpretation that Mg^2+^ extraction efficiency is more closely associated with transport-accessible pore architecture than with absolute surface area alone. UF-opt achieves the most favorable textural configuration by combining fluoride-mediated silica destabilization with cavitation-associated surface renewal and pore/surface opening, thereby limiting pore blockage and sustaining higher transport accessibility. While BET surface area and pore-size distributions quantify pore size and volume evolution, they do not capture nanoscale surface roughness within these pores [101]; this limitation motivates the fractal analysis presented in the following section.

#### 3.7.2 Fractal analysis of pore-surface complexity (FHH model)

FHH analysis complements the BET/NL-DFT results by providing a heuristic descriptor of nanoscale pore-wall irregularity, using the procedure described in Section 2.3.2. The extracted surface fractal dimensions span a narrow but systematic range (D_s_ = 2.503 − 2.610), with excellent linearity within the selected fitting window (R^2^ ≥ 0.996), supporting the applicability of the FHH representation over this relative-pressure range, as shown in Table 7.

**Table 7:** Surface fractal parameters derived from FHH analysis of N_2_ adsorption isotherms.

Sample	Slope (D_s_ − 3)	D_s_	Structural character
RS	−0.3905	2.610	High surface heterogeneity
A-opt	−0.4561	2.544	Intermediate roughness
T-opt	−0.4074	2.593	Irregular micro–mesoporous texture
TF-opt	−0.4899	2.510	Regularized mesopores
U-opt	−0.4261	2.574	Ultrasound-modified irregular surface texture
UF-opt	−0.4973	2.503	Lowest D_s_; wider-pore-dominated texture

Notably, the fluoride-activated residues (TF-opt and UF-opt) display the lowest D_s_ values (2.510 and 2.503, respectively), consistent with lower nanoscale pore-wall irregularity and the NL-DFT-observed redistribution toward wider pore domains (Table 6). In contrast, T-opt exhibits a relatively high D_s_ of 2.593, indicating greater nanoscale pore-wall irregularity and a more irregular micro-/mesoporous texture despite its exceptionally high BET surface area [61]. U-opt retains an intermediate-high D_s_ of 2.574, whereas A-opt exhibits an intermediate value of 2.544.

From a transport perspective, the lower D_s_ values observed for the MgF_2_-assisted residues, particularly TF-opt and UF-opt, are consistent with less irregular nanoscale pore-wall geometries relative to T-opt and U-opt. When considered together with the NL-DFT pore-size distributions, this trend supports a shift toward less transport-restrictive residue architectures rather than a simple dependence on total BET surface area.

Although BET/NL-DFT, N_2_ physisorption, and FHH fractal analyses do not directly resolve three-dimensional pore connectivity or tortuosity, their combined trends provide a consistent transport-accessibility interpretation across the investigated leaching pathways. First, the NL-DFT pore-size distributions show that T-opt retains approximately 66% of its measured total pore volume in pores below 10 nm, whereas TF-opt and UF-opt shift toward wider-pore-dominated architectures, with PV > 10 nm reaching 67.3% and 70.9%, respectively (Table 6). Because narrow pores are more susceptible to wall interactions, steric restriction, and reduced effective diffusivity of hydrated ionic species, this redistribution is consistent with lower internal transport restriction under MgF_2_-assisted conditions [17], [60], [97]. Second, the lower D_s_ values of TF-opt and UF-opt indicate less irregular nanoscale pore-wall geometries, complementing the pore-size redistribution observed by NL-DFT [61], [64]. The FHH surface-fractal dimension D_s_ is used here as a heuristic descriptor of pore-wall roughness/geometric irregularity rather than as a direct tortuosity measurement [61], [64], [102]. By contrast, T-opt retains a relatively elevated D_s_ of 2.593 despite its high BET surface area, supporting the interpretation that much of the thermally generated surface area is associated with narrow and irregular pore domains rather than with an open transport network. Third, the stronger high-relative-pressure uptake and broader hysteresis observed for TF-opt and UF-opt provide qualitative support for the development of more open mesoporous domains, while recognizing that H_3_-type hysteresis in phyllosilicate-derived residues also reflects slit-shaped pore geometry rather than pore-network connectivity alone [99]. Taken together, these descriptors support the interpretation that Mg^2+^ extraction follows transport-accessible pore architecture more closely than BET surface area alone.

Together with the BET and NL-DFT results in Section 3.7.1, the FHH analysis therefore indicates that UF-opt combines a redistribution toward wider pore domains with the lowest measured nanoscale pore-wall irregularity among the tested residues. These complementary textural changes are consistent with reduced geometric transport restriction and support the broader mechanistic relationship between residue architecture and mass-transfer accessibility under intensified conditions.

#### 3.7.3 FT-IR evidence of lattice destabilization and silica framework reorganization

**Fig. 9:**
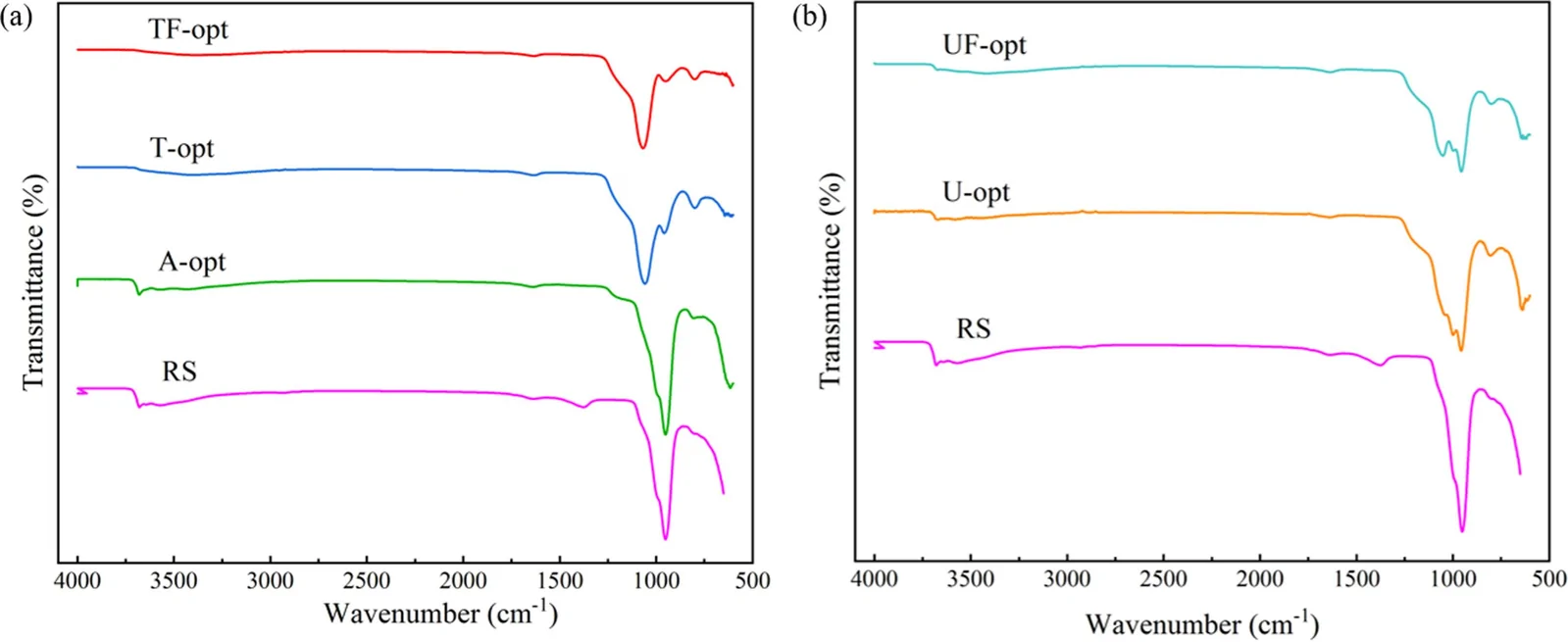
FT-IR spectra of lizardite residues under different activation pathways. (a) Conventional leaching route (RS, A-opt, T-opt, TF-opt). (b) Ultrasound-assisted leaching route (RS, U-opt, UF-opt).

FT-IR spectroscopy, as shown in Fig. 9, provides molecular-level evidence for progressive lattice destabilization and silica network reorganization under the investigated leaching pathways. The spectral evolution clarifies the distinct roles of thermal leaching, fluoride-mediated chemistry, and ultrasonic cavitation in governing silicate polymerization, depolymerization, and framework permeability.

The RS exhibits the characteristic O–H stretching doublet at ∼3683 and ∼3576 cm^−1^, corresponding to inner-surface Mg–OH stretching and structural hydroxyls of the 1:1 lizardite framework. The sharpness and intensity of this doublet are consistent with an intact octahedral Mg–OH sheet and well-preserved layered crystallinity [103], [104], [105], [106]. The dominant Si–O asymmetric stretching band at ∼951 cm^−1^ represents the tetrahedral-sheet vibration characteristic of crystalline lizardite, typical of layered phyllosilicates (often associated with predominantly Q^3^-type environments in serpentines) [80], [107]. Following ambient leaching (A-opt), the O–H stretching doublet remains largely preserved, indicating that the lizardite lattice retains structural hydroxyls under mild conditions. Minor broadening of the Si–O band and the appearance of a weak feature near ∼807 cm^−1^ suggest incipient silanol formation and limited surface reorganization. These changes are consistent with surface-dominated/near-surface dissolution with limited lattice disruption and restricted internal transport [80].

The T-opt induces pronounced spectral changes. The structural O–H bands are strongly attenuated, consistent with dissolution-driven lattice breakdown and de-magnesiation. Simultaneously, the dominant Si–O band shifts toward higher wavenumbers (∼956–1054 cm^−1^), accompanied by increased intensity of symmetric Si–O–Si vibrations near ∼790–800 cm^−1^ [80], [107], [108]. The band broadening and progressive blue-shift are consistent with silica restructuring toward higher network connectivity; however, the persistence of a relatively structured envelope differs from the broader ∼1080–1082 cm^−1^ signature observed under fluoride activation (see below), which indicates a more disordered amorphous framework. This shift toward higher wavenumbers is consistent with increased siloxane polymerization and a more cross-linked amorphous silica network [109], [110], [111]. Broad bands near ∼3400 and ∼1640 cm^−1^ become more pronounced, corresponding to adsorbed water and silanol-associated hydroxyl groups on newly formed amorphous silica surfaces [108]. The enhancement of these hygroscopic water bands is consistent with formation of a silica-rich, polymerized residue and correlates with the increased internal surface exposure and development of microporous silica domains observed in N_2_ physisorption analyses [9], [80].

Collectively, these features support the formation of a silica-rich, partially polymerized amorphous residue, consistent with framework densification and silica-rich transport restriction, providing a molecular explanation for the diffusion limitations observed under T-opt despite the large increase in BET surface area [80], [112], [113].

In contrast, U-opt does not introduce distinct new bands; changes are primarily intensity/broadening relative to the conventional systems. The spectral modifications are limited to modest attenuation and slight broadening of existing O–H and Si–O features, consistent with ultrasound-associated physical surface modification and enhanced exposure of reactive surfaces [48]. These observations align with literature describing ultrasonic leaching as predominantly a cavitation-driven physical intensification process, enhancing mass transfer and interfacial renewal, rather than inducing new solid-phase bonding environments [14], [114]. Accordingly, ultrasound primarily improves accessibility and transport behavior without producing distinct new FT-IR-resolved bonding environments.

MgF_2_-assisted systems (TF-opt and UF-opt) exhibit a distinctly different spectral evolution dominated by fluoride-mediated chemistry. In these samples, the structural O–H stretching bands are strongly attenuated (particularly in the TF-opt system), indicating extensive dissolution-driven loss of the hydroxyl-bearing lizardite framework (de-magnesiation and structural collapse) [113]. The dominant Si–O stretching band shifts toward higher wavenumbers (∼1080–1082 cm^−1^) and broadens markedly, consistent with the formation of a highly disordered amorphous silica network and loss of long-range order [20], [37], [115]. Concurrent enhancement of the ∼793 cm^−1^ symmetric Si–O–Si vibration further confirms rearrangement of the tetrahedral framework following structural breakdown [9], [80], [107]. This spectral evolution parallels that reported for fluoride-modified silicate systems, where F^−^ perturbs Si–O–Si bond angles and promotes the formation of non-bridging oxygen (NBO)-rich environments, thereby limiting complete siloxane network densification/cross-linking and maintaining a partially depolymerized, more permeable silicate structure [9], [116], [117].

Notably, additional features appear in the ∼936–945 cm^−1^ region for the MgF_2_-assisted residues. This region is mechanistically important because bands in this range have been reported for Si–F stretching and/or NBO-associated Si–O^−^ vibrations in fluoride-modified silicate networks [9], [118], [119], [120], [121]. In the present system, the emergence of these features, together with attenuation of the Mg–OH/O–H bands at ∼3683 and ∼3576 cm^−1^ and reorganization of the Si–O stretching envelope from the crystalline lizardite fingerprint near ∼951 cm^−1^ toward a broader amorphous silica-related band near ∼1080–1082 cm^−1^, supports fluoride-mediated modification of the silicate framework. These changes are consistent with Si–F/NBO-associated environments, partial disruption of long-range siloxane connectivity, and suppression of complete siloxane network densification [109], [110], [111].

Because the ∼936–945 cm^−1^ region is not uniquely diagnostic of Si–F versus NBO-associated Si–O^−^ environments, direct chemical-state confirmation remains outside the scope of the present study. Future work should apply high-resolution X-ray photoelectron spectroscopy (XPS) analysis of the Si 2p, O 1s, F 1s, and Mg 2p regions, using replicate residue sampling and, where feasible, depth-sensitive measurements, to evaluate the near-surface chemical environments associated with fluoride-mediated silicate modification.

Mechanistically, fluoride-bearing species generated from MgF_2_ under acidic conditions, including pH-dependent HF/F^−^ species, are expected to promote acid-assisted cleavage of Mg–O–Si and Si–O–Si linkages [9], [41], [118], accelerating lattice destabilization under comparatively mild conditions (∼80–85 °C) [9], well below temperatures required for thermal serpentine dehydroxylation (600–800 °C); here the OH-band loss reflects chemical dissolution/structural breakdown rather than thermally driven dehydroxylation [14], [122]. This bond cleavage facilitates Mg^2+^ release and is consistent with formation of fluoride-modified silicate environments, including Si–F and NBO associated species, yielding a porous amorphous silica residue [9], [123]. The close similarity between TF-opt and UF-opt spectra indicates that fluoride chemistry is the dominant driver of structural transformation, while ultrasound primarily enhances interfacial transport and may help limit localized gel densification.

Overall, the FT-IR results indicate that Mg^2+^ extraction is controlled by the competition between silica polymerization, which promotes diffusion-resistant, highly cross-linked siloxane networks, and fluoride-mediated depolymerization, which preserves more permeable fluoride-modified silicate environments. Thermal leaching alone favors framework densification and silica-rich transport restriction, whereas MgF_2_ redirects the reaction pathway toward a chemically destabilized amorphous silica matrix under milder conditions. Ultrasound acts as a complementary mechanical intensification mechanism that sustains accessibility within this fluoride-modified framework. This interpretation directly supports the reduced surface fractal dimension discussed in Section 3.7.2 and the enhanced volumetric productivity observed for the UF-opt system. Detailed vibrational assignments are provided in Table S5.

#### 3.7.4 Phase evolution and crystallinity loss (XRD)

**Fig. 10:**
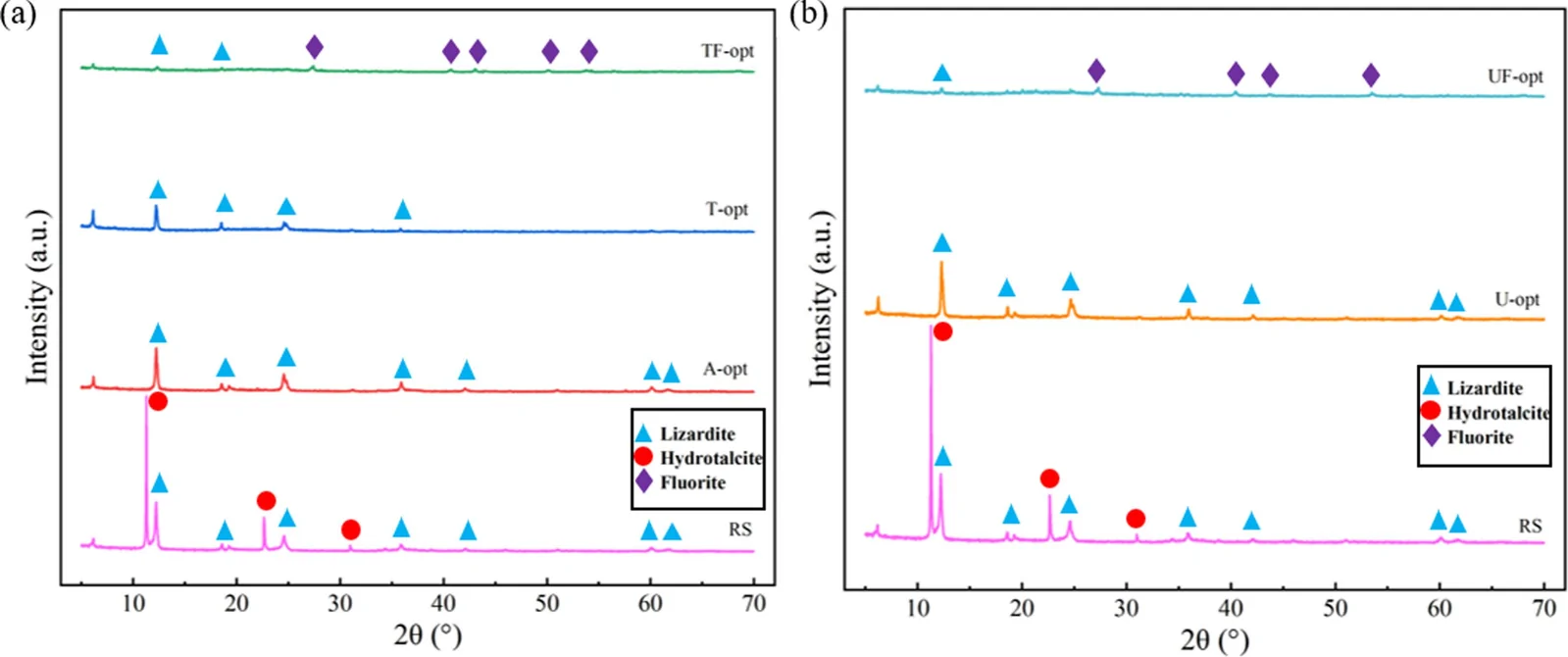
XRD patterns illustrating phase evolution and crystallinity loss of lizardite-rich serpentine tailings under different leaching pathways: (a) RS, A-opt, T-opt, and TF-opt; (b) RS, U-opt, and UF-opt. Progressive attenuation and broadening of lizardite reflections, together with development of a diffuse scattering background in MgF_2_-assisted systems, indicate increasing lattice disorder and loss of long-range phyllosilicate order. Patterns are vertically offset for clarity.

XRD patterns were employed to elucidate phase evolution and crystallinity loss under the different leaching pathways, as shown in Fig. 10. The RS is dominated by sharp and well-defined reflections characteristic of lizardite, including the basal (001) reflection at 2θ ≈ 12° (d ≈ 7.3 Å) and higher-order (002) and (00ℓ) reflections near 24° and 36°, consistent with a highly ordered layered phyllosilicate structure [113], [124]. The pronounced intensity and narrow peak widths indicate high crystallinity and long-range stacking order within the octahedral–tetrahedral sheets. In addition, weak reflections at approximately 11.3° and 22.5° 2θ are assigned to hydrotalcite-like layered double hydroxide (LDH) phases, commonly formed as secondary Mg–Al–Fe hydroxides in ultramafic tailings [125], [126], [127]. The coexistence of crystalline lizardite and minor LDH phases confirms a structurally heterogeneous feedstock, consistent with the bulk elemental composition and prior mineralogical characterization.

After ambient leaching (A-opt), the principal lizardite reflections remain clearly discernible, with only slight peak attenuation and limited broadening. This indicates that the layered phyllosilicate framework largely retains its long-range crystallinity under mild conditions. In contrast, the LDH-related reflections are no longer discernible, indicating that these secondary Mg–Al–Fe hydroxide phases are acid-labile and dissolve preferentially [126], [128], [129]. The selective removal of LDH phases without substantial collapse of the lizardite lattice explains the modest Mg^2+^ extraction observed at ambient temperature. Mg^2+^ release under A-opt appears to be influenced substantially by dissolution of structurally accessible secondary hydroxides, while the primary lizardite framework remains largely intact.

T-opt produces markedly stronger attenuation and broadening of lizardite reflections, particularly the basal (001) peak, indicating reduced coherent domain size, disruption of stacking order, and progressive lattice disorder consistent with acid-promoted Mg^2+^ extraction and partial amorphization [80]. While identifiable lizardite peaks remain detectable, indicating that the crystalline framework is not fully deconstructed, the structural evolution is substantial. This incomplete structural collapse aligns with the FT-IR evidence of enhanced siloxane polymerization and increased network connectivity, suggesting that selective de-magnesiation of the octahedral sheet promotes silica densification rather than full framework disintegration [78]. Consequently, although BET surface area increases substantially under T-opt, the formation of a partially polymerized silica-rich residue is consistent with significant internal diffusion resistance, in agreement with the transport constraints inferred from textural analysis [97].

On the other hand, U-opt produces intermediate structural modification. Lizardite reflections remain discernible but are uniformly weakened and broadened relative to RS, indicating defect generation, reduced stacking coherence, and increased lattice disorder consistent with cavitation-assisted physical disruption and enhanced surface exposure [48]. However, the absence of a dominant amorphous halo confirms that ultrasound alone does not induce complete structural deconstruction. These observations can indicate that acoustic cavitation enhances structural accessibility without fundamentally collapsing the crystalline framework.

In contrast, MgF_2_-assisted systems (TF-opt and UF-opt) exhibit strong attenuation of characteristic lizardite reflections, with only weak residual crystalline features remaining. In both cases, the diffractograms are dominated by a broad diffuse scattering feature centered at ∼22–28° 2θ, characteristic of short-range Si–O correlations in silica-rich amorphous residues. This diffuse halo indicates extensive loss of long-range phyllosilicate order and is consistent with advanced framework destabilization. Weak residual reflections labeled “MgF_2_” are attributed to trace crystalline unreacted MgF_2_ additive rather than newly formed crystalline fluoride phases, suggesting that fluoride predominantly acts as a chemical modifier promoting structural breakdown, with limited evidence for formation of secondary crystalline fluoride phases [53], [55]. The pronounced amorphization observed in TF-opt and UF-opt coincides with the highest Mg^2+^ extraction efficiencies (up to ∼85.6% for UF-opt), supporting a strong link between extensive lattice collapse/silicate depolymerization and improved leaching performance [9], [15], [37]. Notably, the nearly featureless UF-opt diffractogram, comparable to TF-opt, indicates that fluoride chemistry governs the dominant structural transformation, while ultrasound contributes by enhancing transport and interfacial renewal under mild bulk-temperature conditions.

Overall, the XRD results show a consistent progression from surface-limited alteration (A-opt) to partial disordering (T-opt and U-opt), and finally to extensive amorphization under MgF_2_-assisted activation (TF-opt and UF-opt) systems. The emergence of an amorphous halo provides direct structural evidence for formation of a silica-rich disordered framework, corroborating the fluoride-mediated silica reorganization inferred from FT-IR analysis and the pore-architecture evolution observed by N_2_ physisorption. Collectively, this convergence of crystallographic, spectroscopic, and textural evidence supports the conclusion that high Mg^2+^ extraction correlates with progressive loss of long-range lizardite order and development of a silica-rich amorphous framework.

#### 3.7.5 Surface morphology and microstructural evolution (SEM)

**Fig. 11:**
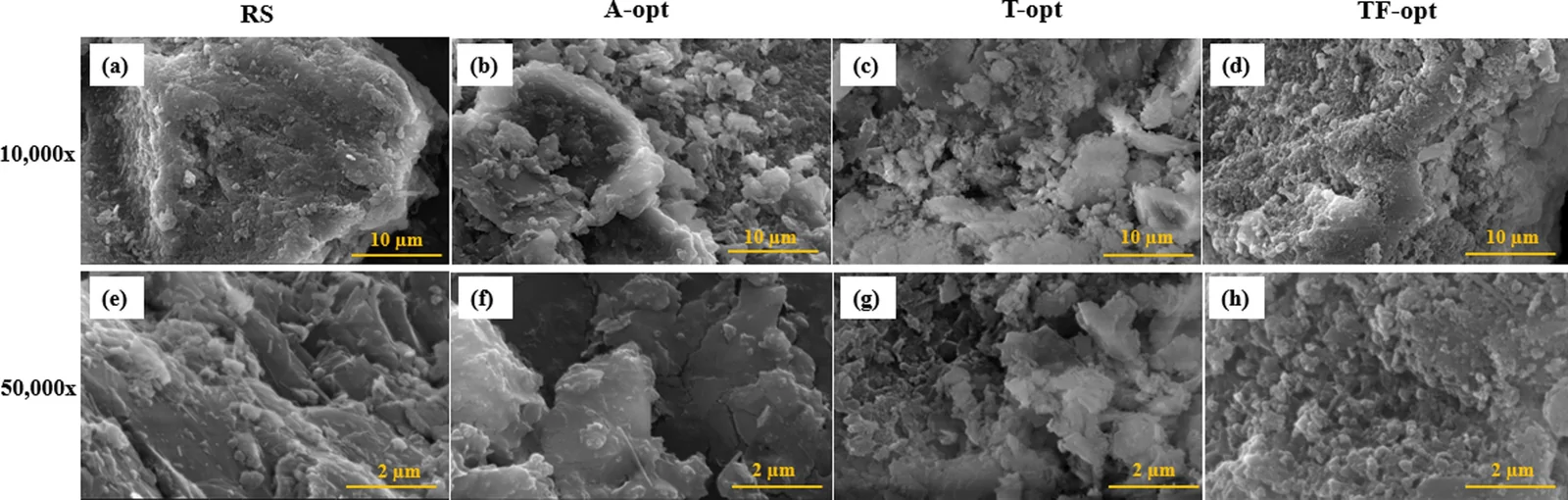
SEM micrographs illustrating the progressive microstructural evolution of lizardite under conventional leaching pathways at 10,000× (a–d) and 50,000× (e–h) magnifications, highlighting the transition from intact lamellar stacks (RS) to surface-etched sheets (A-opt), fragmented, compact transport-restrictive aggregates (T-opt), and extensive structural disruption under MgF_2_-assisted thermal leaching (TF-opt), yielding a destabilized silica-rich residue with wide pore development but locally compact/gel-like domains that may still impose partial transport constraints.

SEM micrographs obtained at 10,000× and 50,000× magnifications (Fig. 11) reveal a systematic evolution of surface morphology along the conventional leaching pathway, progressing from intact lamellar stacks to progressively disordered and chemically destabilized residues under different conditions. The RS displays the characteristic plate-like morphology of lizardite, consisting of compact aggregates with smooth basal surfaces and tightly stacked lamellae [130], [131]. No discernible macro-scale porosity or extensive interlayer separation is observed at the examined magnifications. This morphology is consistent with the sharp lizardite reflections in XRD and the low BET surface area, supporting a dense and structurally coherent framework. Such compact stacking limits acid penetration and explains the low Mg^2+^ extraction efficiency of the untreated material.

Following ambient leaching (A-opt), the lamellar architecture remains largely preserved; however, particle surfaces exhibit noticeable roughening, shallow etch pits, and localized cracking. At higher magnification (Fig. 11f), micro-fractures are observed preferentially along lamellar boundaries, indicating partial delamination and defect formation. These fractures remain spatially confined rather than penetrating across entire particles, consistent with restricted dissolution fronts and limited structural propagation. Overall, A-opt introduces surface disorder with incipient etching/defect features while preserving the bulk lamellar framework, in agreement with surface-controlled Mg^2+^ dissolution and modest extraction efficiency [77], [112].

T-opt induces substantially greater microstructural degradation. At 10,000× (Fig. 11c), the original lamellar plates are no longer preserved as discrete stacks and instead appear as fragmented, angular plate-like debris embedded within compact agglomerates. At 50,000×, as shown in Fig. 11g, the residue is dominated by roughened plate fragments and abundant fine particulate material, with locally compacted or coalesced regions that are consistent with reduced lamellar openness and a more transport-restrictive pore architecture [132]. This morphology can be consistent with progressive lattice disruption accompanied by formation of a dense secondary residue (inferred from the coupled XRD/FT-IR evidence) rather than development of well-connected open porosity [77]. Although BET surface area increases substantially under T-opt, this increase is primarily associated with the development of microporous and small mesoporous domains rather than formation of wide mesoporous pathways or transport-accessible pore architecture required for efficient mass transport.

As shown in Table 6, approximately two-thirds of the total pore volume is distributed within pores smaller than 10 nm. Such fine pores can significantly increase surface area but do not necessarily provide efficient diffusion pathways. The compacted aggregate texture therefore suggests a more restricted internal transport architecture despite the high BET value. This interpretation aligns with XRD peak broadening and FT-IR results, supporting partial amorphization coupled with silica densification rather than complete framework disintegration [133].

The most pronounced structural collapse among the conventional systems is observed for TF-opt. At 10,000× (Fig. 11d), the lamellar architecture is no longer discernible and is replaced by irregular, fine-textured agglomerates. At 50,000× (Fig. 11h), the surfaces are dominated by coalesced fine-scale domains and amorphous-like coatings that obscure residual crystalline features. This morphology reflects extensive framework breakdown accompanied by locally compact, coating-like fine-textured domains consistent with the silica-rich amorphous residue inferred from XRD/FT-IR, which may partially restrict pore connectivity. These coating-like domains may contribute to localized diffusion limitations, providing a plausible microstructural basis for the slightly lower extraction efficiency compared to UF-opt. The more extensive disruption observed in TF-opt correlates with the near-complete suppression of characteristic lizardite reflections in XRD and the disappearance of structural O–H bands in FT-IR, supporting that MgF_2_-assisted leaching promotes substantially greater lattice destabilization under thermal MgF_2_-assisted conditions [9], [37], [42].

**Fig. 12:**
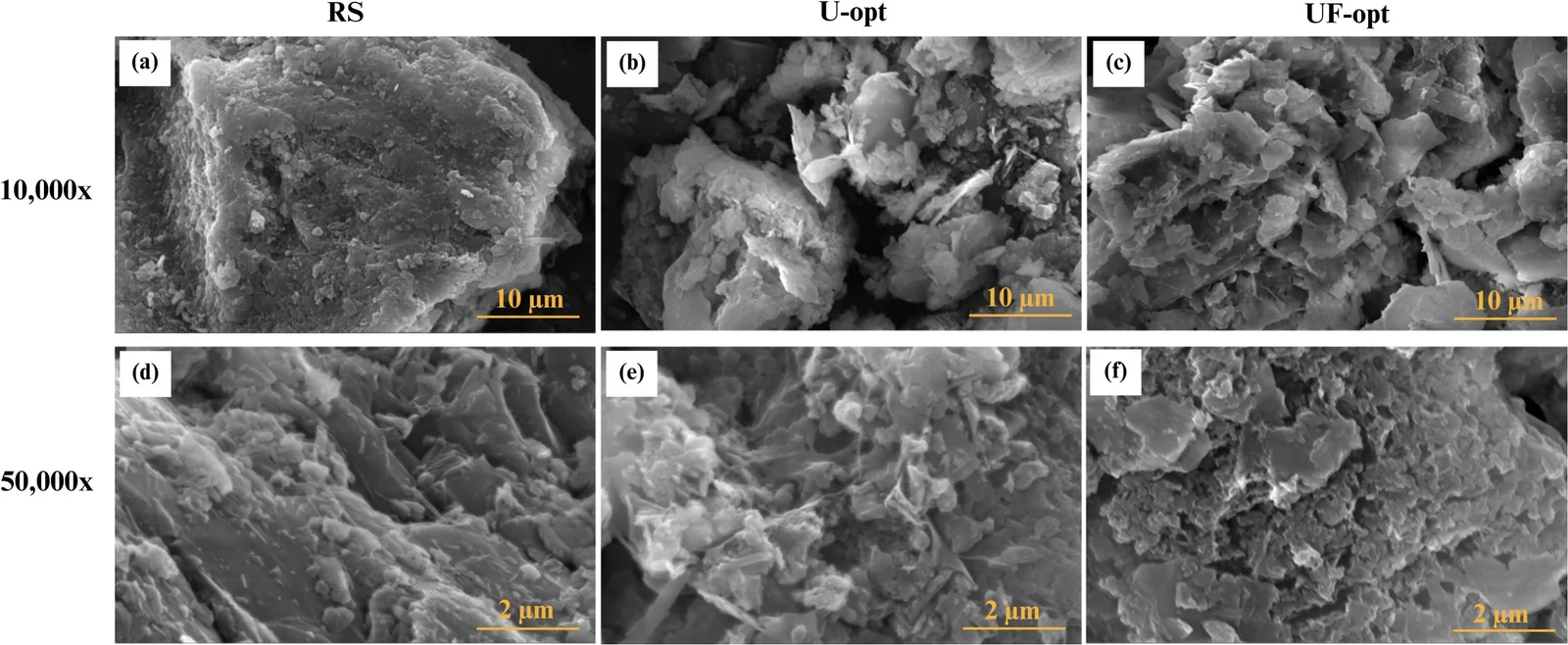
SEM micrographs illustrating microstructural evolution under ultrasound-assisted pathways at 10,000× (a–c) and 50,000× (d–f) magnifications. U-opt shows lamellar exfoliation and defect generation, whereas UF-opt exhibits extensive structural disruption and development of a highly porous, fine-textured residue consistent with the silica-rich amorphous phase inferred from XRD and FT-IR. The more homogeneous and perforated morphology of UF-opt is consistent with coupled mechanical and chemical effects during leaching.

As shown in Fig. 12, in contrast to thermally driven degradation, U-opt results in a distinct microstructural modification that appears largely mechanical in character. At 10,000× (Fig. 12b), particles are more fragmented and less agglomerated than RS, suggesting reduced effective particle size consistent with cavitation-related mechanical stress [48]. At 50,000× (Fig. 12e), lamellar peeling and partial exfoliation features are observed, with thinner platelets and micro-fractured edges indicative of interlayer delamination [48]. These features are more consistent with mechanical defect generation than with extensive chemical dissolution of the silicate framework. The persistence of recognizable layered fragments suggests that ultrasound enhances structural accessibility without inducing complete lattice collapse [50]. This interpretation can align with the attenuated yet detectable lizardite reflections in XRD and the moderate increase in BET surface area.

In contrast to U-opt, UF-opt exhibits far more extensive structural breakdown, arising from the combined action of cavitation and fluoride-mediated chemical destabilization. At 10,000× (Fig. 12c), the lamellar architecture is no longer clearly distinguishable and is replaced by highly fractured aggregates. At 50,000× (Fig. 12f), the residue appears as a disrupted matrix with abundant fine-scale voids, perforated domains, and exposed edge features [37]. Compared with TF-opt, which shows locally compact gel-like regions, the UF-opt residue appears more uniformly disrupted and less dominated by dense surface coatings. This observation is consistent with improved structural accessibility and potentially reduced accumulation of silica-rich alteration domains under combined cavitation and fluoride effects. The resulting hierarchically accessible residue is consistent with the near-featureless XRD patterns, broadened FT-IR bands, and the highest pore volume measured by BET.

Collectively, these SEM observations suggest that fluoride-mediated chemistry is the primary driver of lattice destabilization, while ultrasound enhances surface renewal, lamellar-edge exposure, and morphological opening. The combined effect produces the most extensively disrupted and texturally accessible residue among the tested conditions, consistent with the highest Mg^2+^ extraction efficiency observed for UF-opt, as supported by other studies [37], [48], [50], [88]. To quantitatively evaluate whether these visual differences correspond to measurable particle-size reduction and surface-complexity changes, early-stage PSD analysis and SEM/ImageJ quantification are presented in the following section.

#### 3.7.6 Comparative PSD and SEM/ImageJ quantification of ultrasound-associated particle and surface modification

To distinguish ultrasound-induced surface modification from morphology changes driven primarily by Mg^2+^ dissolution extent, T-opt and U-opt residues were compared directly because they reached near-equivalent Mg^2+^ extraction (49.45% and 47.62%) through different pathways: U-opt within 60 min at < 40 °C, versus T-opt requiring 180 min at 85 °C. Because both systems are MgF_2_-free, this comparison reduces the confounding influence of MgF_2_-assisted chemical effects, and comparison at similar extraction provides a basis for assessing whether ultrasound produces surface features beyond those expected from dissolution extent alone, despite the different temperature–time histories.

Matched-magnification SEM images of U-opt and T-opt residues at 10,000×, 30,000×, and 50,000× are provided in Fig. S6. Across this sequence, U-opt displays more clearly exposed lamellar faces, open interlamellar domains, sheet-edge development, and localized physical disruption, whereas T-opt exhibits a more compact, roughened, stacked aggregate morphology in which lamellar features remain visible but appear less open and are partly masked by fine roughening and coating-like domains. This visual comparison supports the interpretation that ultrasound promotes lamellar-edge exposure and surface opening, while thermal leaching produces a more compact roughened residue.

The textural results reinforce this finding. T-opt combined compact roughened aggregates with a high BET surface area (301.9 m^2^ g^−1^), total pore volume of 0.279 cm^3^ g^−1^, a low wide-mesopore fraction (33.9% PV > 10 nm), and high surface fractal dimension (Ds = 2.593), indicating extensive thermal roughening within a micro-/small-mesopore-dominated, transport-restrictive silica-rich residue. U-opt instead showed pronounced lamellar-face exposure and sheet-edge development with a lower BET surface area (164.0 m^2^ g^−1^) and pore volume (0.193 cm^3^ g^−1^) but a higher wide-mesopore fraction (49.0% PV > 10 nm) and slightly lower Ds (2.574). This divergence at comparable Mg^2+^ extraction indicates that ultrasound does not reproduce thermally driven silica-rich densification or maximize surface area, but modifies the residue evolution toward more open, transport-accessible mesoporous features.

Semi-quantitative Fiji/ImageJ analysis of representative high-contrast 10,000 × SEM fields supported this distinction [67]. Using the same skeletonization workflow, the apparent edge-density index *D*_edge_ rose from 3.087 ± 0.464 µm^−1^ (T-opt) to 4.178 ± 0.685 µm^−1^ (U-opt), and branch-point density *D*_branch_ from 10.289 ± 1.695 to 13.806 ± 2.449 junctions µm^−2^, corresponding to approximately 35% and 34% higher mean values, respectively, at near-equivalent extraction. Because SEM contrast depends on particle orientation, topography, thresholding, and shadowing, *D*_edge_ and *D*_branch_ are treated as semi-quantitative relative descriptors of edge/corner complexity rather than absolute crack-density or inert-layer measurements [68]. Considered with the BET/NL-DFT and FHH results, they support the interpretation that ultrasound-assisted processing modifies the residue surface beyond dissolution extent alone, with changes consistent with interfacial renewal, lamellar-edge exposure, and transport-accessible pore development. Representative processing examples (original, edge-detected, thresholded/binary, and skeletonized SEM images) are provided in Fig. S3.

To test whether ultrasound produces measurable particle-scale modification at an early stage, 30 min validation residues were prepared for paired PSD and SEM/ImageJ analysis: U30 versus T30 (MgF_2_-free) and UF30 versus TF30 (MgF_2_-assisted), with a remeasured raw aliquot (RS) as the internal PSD baseline. This provides a same-duration comparison of ultrasound-assisted and non-ultrasound routes; as the thermal residues were prepared at 85 °C and the ultrasound-assisted residues below 40 °C, the comparison is matched on reaction time rather than temperature.

PSD showed a systematic decrease in D50 and D90 in the order RS > T30 > U30 > TF30 > UF30 (Fig. S7 and Table S6). This overall sequence is descriptive only and is not an ultrasound-only trend, since the residues differ in thermal history, MgF_2_ dosage, and cavitation exposure; the informative comparisons are therefore the paired routes T30 vs U30 and TF30 vs UF30. In the MgF_2_-free pair, U30 reduced D50 from 37.1 to 32.9 µm and D90 from 165 to 154 µm (11.3% and 6.7%) relative to T30. In the MgF_2_-assisted pair, UF30 produced a stronger shift than TF30, reducing D50 from 29.0 to 23.3 µm and D90 from 142 to 124 µm (19.7% and 12.7%). These paired comparisons provide particle-scale evidence that ultrasound contributes to early-stage size reduction relative to the corresponding non-ultrasound residues, while recognizing that the MgF_2_-assisted pair also reflects the optimized additive dosages used for each route. PSD alone is not taken as direct proof of delamination; it is interpreted alongside the SEM/ImageJ surface-complexity metrics. Full PSD descriptors (D10, D50, D90, D [2, 3], D [3, 4]) are provided in Table S6.

The SEM/ImageJ results were interpreted within the same paired framework (U30 vs T30; UF30 vs TF30), with cross-condition comparisons excluded as primary evidence because they combine thermal, dosage, and cavitation differences; the metrics are summarized in Table S7. Relative to T30, U30 increased *D*_edge_ from 1.444 ± 0.480 to 3.285 ± 0.886 µm^−1^ and *D*_branch_ from 3.955 ± 1.787 to 11.113 ± 3.342 junctions µm^−2^, indicating higher mean visible edge/corner complexity at equal reaction time without MgF_2_. Relative to TF30, UF30 showed the highest values of the validation set, increasing *D*_edge_ from 3.840 ± 0.584 to 4.726 ± 1.183 µm^−1^ and *D*_branch_ from 12.830 ± 2.068 to 15.332 ± 3.907 junctions µm^−2^, suggesting additional edge development even where MgF_2_-assisted destabilization was already active. The higher values of TF30 relative to U30 are consistent with the added contribution of MgF_2_-assisted chemical etching under thermal leaching, but this cross-condition contrast is not used as primary evidence; the critical paired comparisons remain U30 > T30 and UF30 > TF30. Individual crack counts were not reported as an independent metric, because crack-like features could not be consistently distinguished from lamellar edges, particle boundaries, grooves, and shadowing; skeletonized edge- and branch-point densities were used instead as more reproducible descriptors of fragmentation-related surface modification. Overall, the 30 min PSD comparison supports early-stage particle-size reduction under ultrasound relative to the corresponding non-ultrasound routes, while the SEM/ImageJ statistics show concurrent increases in edge and branch-point complexity, together supporting the interpretation that ultrasound-assisted processing promotes particle/surface modification, lamellar-edge exposure, and transport-accessible surface renewal during acid leaching.

Future work should examine residues collected within the first 5–10 min using AFM for surface-roughness mapping and FIB-SEM cross-sectioning coupled with EDS compositional line scanning or mapping of Si and Mg to directly evaluate early surface-topography changes and the thickness, composition, and spatial heterogeneity of Mg-depleted, silica-rich alteration domains under silent and ultrasound-assisted conditions.

#### 3.7.7 Integrated mechanistic interpretation

**Fig. 13:**
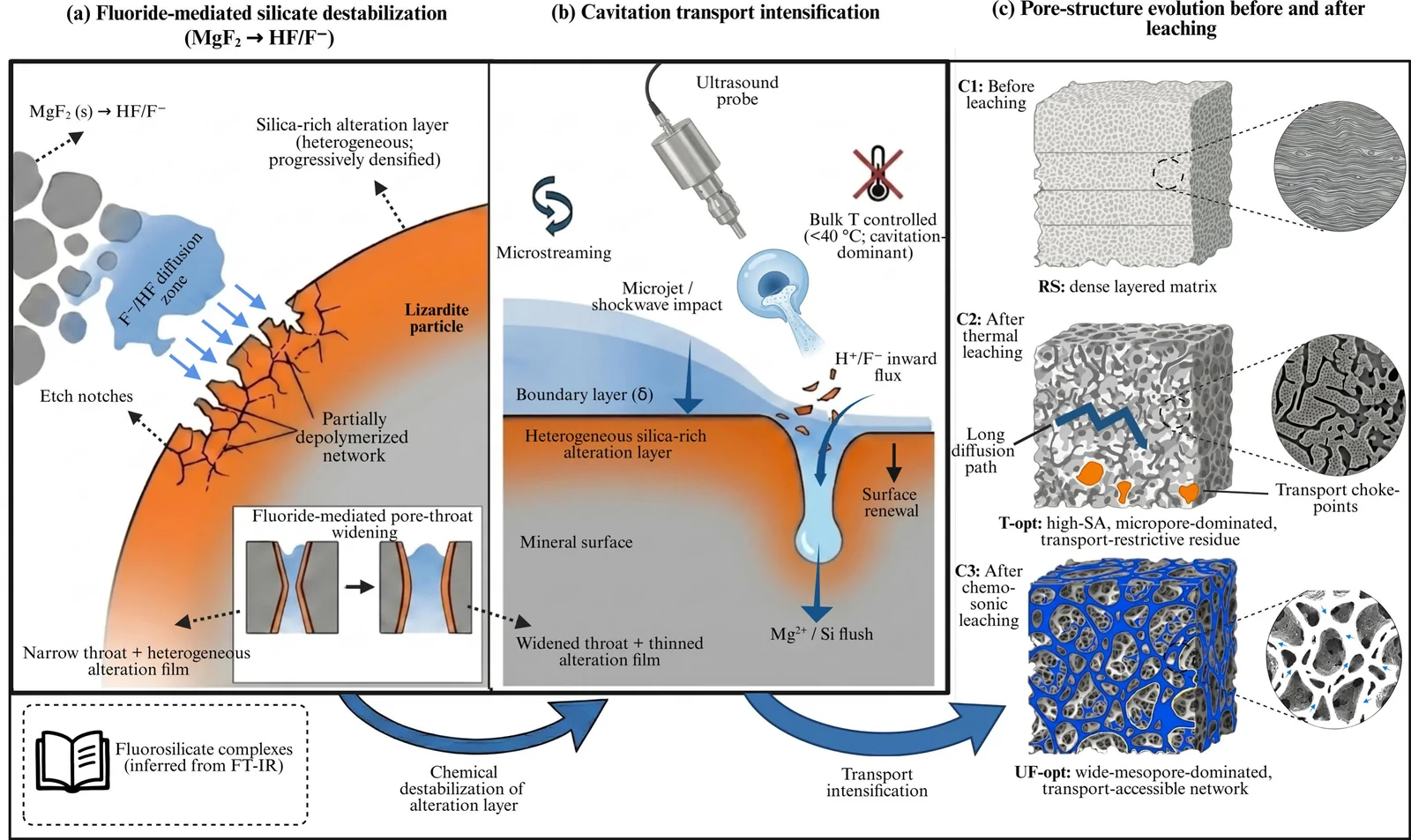
Proposed integrated mechanistic schematic linking fluoride-mediated silicate destabilization, ultrasonic cavitation, and residue-architecture transition during Mg^2+^ extraction from lizardite. (a) MgF_2_-derived fluoride species (HF/F^−^) interact with silica-rich alteration domains, promoting silicate destabilization, etch-notch development, and pore-throat widening. (b) Cavitation-generated microjets and microstreaming renew the reacting interface and enhance inward H^+^/F^−^ transport while facilitating outward transport of Mg^2+^ and dissolved Si species. (c) RS retains a dense layered structure; T-opt develops a high-surface-area but transport-restrictive micro-/small-mesoporous architecture; UF-opt evolves toward a transport-accessible, wide-mesopore-dominated architecture despite its lower BET surface area than T-opt. Circular insets show representative internal residue textures.

**Fig. 14:**
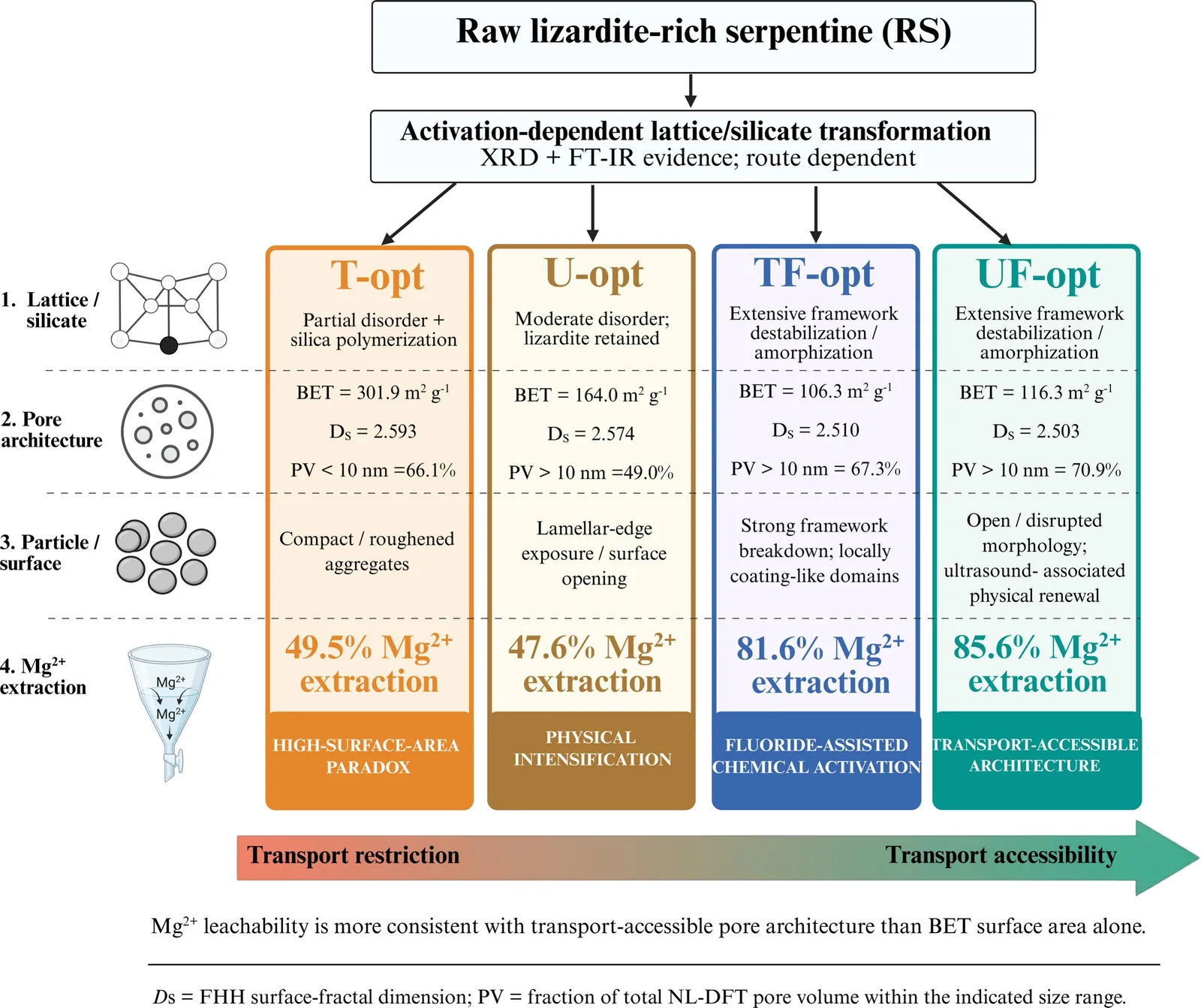
Cross-scale characterization framework linking lattice/silicate transformation, pore architecture, particle/surface modification, and Mg^2+^ leachability across T-opt, U-opt, TF-opt, and UF-opt. The comparison highlights the high-specific-surface-area paradox of T-opt and the development of wider, less irregular, and more transport-accessible pore architectures under MgF_2_-assisted activation. D_s_ denotes the FHH surface-fractal dimension, and PV denotes the fraction of total NL-DFT pore volume within the indicated size range. Mg^2+^ extraction values correspond to the route-specific conditions and are shown to relate residue architecture to process response rather than as a temperature- or residence-time-matched kinetic comparison.

Integration of the crystallographic, spectroscopic, textural, particle-size, and morphological datasets establishes a progressive cross-scale relationship linking (i) lizardite-framework and silicate-network transformation, (ii) evolution of the silica-rich residual/alteration framework, (iii) pore-size distribution and pore-wall roughness, (iv) particle- and surface-scale modification, and (v) macroscopic Mg^2+^ extraction. XRD and FT-IR define the extent and nature of lattice/silicate-network transformation; BET/NL-DFT and FHH characterize the resulting pore architecture and surface irregularity; PSD, SEM, and SEM/ImageJ describe particle- and surface-scale modification; and Mg^2+^ extraction provides the corresponding process response. Fig. 13 illustrates the proposed chemical and physical mechanisms governing fluoride-assisted and ultrasound-assisted intensification, whereas Fig. 14 integrates the multi-scale characterization evidence into a progressive structure–transport–leachability framework across the four principal intensified pathways.

Under A-opt, reaction remains largely confined to exposed surfaces and acid-labile domains. XRD confirms preservation of long-range lizardite order, FT-IR shows retention of structural O–H bands, and SEM reveals only surface roughening and localized cracking. These observations indicate surface-controlled Mg^2+^ dissolution with limited lattice disruption and restricted internal transport.

T-opt increases structural disorder and apparent surface area through partial de-magnesiation and lattice destabilization. However, FT-IR evidence of enhanced siloxane polymerization, XRD peak broadening with residual crystallinity, and SEM observation of compacted aggregates collectively indicate formation of a partially polymerized silica-rich residue. Although BET surface area increases to 301.9 m^2^ g^−1^, the pore-volume distribution remains dominated by pores < 10 nm (≈66%). Such micro- and small mesopores contribute substantially to measured surface area but do not necessarily provide efficient transport pathways for hydrated ionic species. The internal architecture therefore becomes increasingly transport-restricted, consistent with silica-rich, transport-limiting alteration framework associated with advanced siloxane polymerization. This explains why the highest BET surface area does not correspond to the highest Mg^2+^ extraction efficiency.

Fractal analysis further supports this interpretation. The surface fractal dimension (D_s_) for T-opt remains relatively high (2.593), indicating irregular and rough pore walls consistent with diffusion resistance within a disordered gel-like micro–mesoporous network. In contrast, MgF_2_-assisted systems exhibit lower D_s_ values (2.510 for TF-opt and 2.503 for UF-opt), reflecting surface regularization and reduced nanoscale roughness. Thus, structural accessibility improves not only through pore enlargement but also through reduction of interfacial irregularity that contributes to transport resistance.

Taken together, these data resolve the high-specific-surface-area paradox observed for T-opt. Although T-opt exhibits the highest BET surface area of all residues (301.9 m^2^ g^−1^), approximately 66.1% of its measured pore volume is confined to pores below 10 nm and its relatively high D_s_ value (2.593) indicates greater nanoscale pore-wall irregularity than the fluoride-assisted residues. Combined with the compact/roughened morphology observed by SEM, these features indicate that much of the thermally generated surface area is associated with narrow and irregular transport-restrictive domains rather than with an open transport network. Thus, the magnitude of N_2_-accessible BET surface area and the accessibility of pathways governing inward H^+^ transport and outward Mg^2+^ transport are not equivalent descriptors in this system.

In MgF_2_-assisted systems, TF-opt and UF-opt, structural destabilization is markedly intensified. FT-IR indicates extensive loss of structural hydroxyls and modification of the silicate framework; XRD shows strong attenuation of crystalline reflections with only weak residual features remaining; and SEM reveals substantial collapse of the lamellar architecture into fine-textured residues, with TF-opt showing more locally coating-like domains and UF-opt showing a more open, disrupted morphology. These combined observations are consistent with fluoride-mediated disruption of Mg–O–Si environments and silicate-network depolymerization, promoting more extensive lattice breakdown and reducing the development of a dense, transport-limiting silica-rich barrier.

Ultrasound contributes primarily through interfacial and transport intensification. In U-opt, cavitation enhances fragmentation and features consistent with lamellar peeling/delamination without dominant amorphization, increasing structural accessibility while retaining recognizable layered fragments. When combined with fluoride (UF-opt), cavitation enhances reactant penetration and disrupts incipient silica-rich alteration domains, resulting in a more uniformly open and hierarchically accessible pore architecture. Consistent with Table 6, UF-opt exhibits the highest fraction of pores > 10 nm (≈71%) and the largest total pore volume (0.318 cm^3^ g^−1^), despite a BET surface area (116.3 m^2^ g^−1^) substantially lower than that of T-opt.

This structural evolution is reflected in engineering performance. Although T-opt maximizes surface area, UF-opt achieves the highest normalized and absolute STY, indicating that productivity is more closely associated with transport-accessible pore architecture than with surface-area maximization alone. Collectively, the characterization and extraction results show that Mg^2+^ leachability is more closely associated with the accessibility, size distribution, and geometric irregularity of the evolving pore network rather than by BET surface area alone. The resulting cross-scale sequence can therefore be summarized as lattice/silicate transformation → silica-rich residue reorganization → pore-size and pore-wall-roughness evolution → particle/interface accessibility → Mg^2+^ extraction. Thermal activation strongly increases internal surface generation but channels much of this area into narrow and irregular transport-restrictive porosity. MgF_2_-mediated modification redirects residue evolution toward wider and less irregular pore domains, whereas ultrasound contributes primarily through particle/surface modification and interfacial renewal. Their combination in UF-opt produces the most transport-accessible residue architecture and the highest Mg^2+^ extraction among the tested conditions. This interpretation is based on convergence of complementary characterization methods and does not imply direct measurement of alteration-layer thickness, three-dimensional pore connectivity, or cavitation intensity. The chemical origin of this structural transformation, particularly the dynamic role of fluoride in the liquor phase, is further examined in Section 3.8.

### 3.8 Mechanistic role of fluoride and liquor-phase implications

The role and fate of fluoride during MgF_2_-assisted leaching are interpreted by integrating kinetic trends with the solid-state transformations discussed in Section 3.7. Under acidic conditions, MgF_2_ undergoes proton-promoted dissolution, such that fluoride availability is dynamically governed by acid concentration, temperature, interfacial consumption, and acoustic/transport intensification, including cavitation-enhanced mixing and boundary-layer renewal, and is therefore not solely fixed by the nominal additive loading. MgF_2_ is a commercially available inorganic fluoride used in ceramic and optical applications; in the present work, it is evaluated as a sparingly soluble fluoride source for controlled in situ fluoride release during acid leaching.

In the presence of lizardite, fluoride species generated in situ are expected to participate in localized interfacial reactions that promote disruption of Mg–O–Si environments and siloxane linkages. This bond-selective destabilization promotes defect formation and accelerates lattice breakdown without necessarily inducing uniform bulk dissolution of the silica matrix [9]. In strongly acidic fluoride–silicate systems, dissolved fluoride is widely reported to complex with dissolved silica as fluorosilicate species (notably ${SiF}_{6}^{2-}$); accordingly, analogous transient fluorosilicate speciation is expected here, although it was not directly quantified in the present work [9], [15], [42]. Given the appreciable Al_2_O_3_ content of the feed (∼6.8 wt%), competitive Al–F complexation may also act as a fluoride sink and modulate the effective fluoride activity at reactive interfaces [37], [42], [117].

To evaluate how non-Mg cations modulate effective fluoride availability, apparent Al release (total filterable dissolved Al) was quantified in selected MgF_2_-assisted thermal and ultrasound-assisted leachates (Table S1), calculated relative to total feed Al by WDXRF and therefore representing combined acid-labile Mg–Al–Fe hydroxide and resistant silicate-associated reservoirs rather than a single phase. Fluoride-free baselines of ∼26.5–30.4% (65 °C) and ∼36.5–41.5% (85 °C) indicate that Al-bearing phases leach even without fluoride, while in MgF_2_-containing systems Al release rises with dosage and temperature, consistent with fluoride-promoted release of resistant silicate-associated Al and additional Al–F demand. A simplified budget illustrates the competition: at 5 wt% MgF_2_ the total fluoride inventory is ∼0.016 mol F per 10 g solid, whereas the dissolved Al pool is ∼0.0043–0.0056 mol Al (65 °C) and ∼0.0052–0.0061 mol Al (85 °C). Because Al–F complexation can bind more than one fluoride per Al center, this pool could sequester a substantial fraction of the inventory, reducing the fluoride activity available for silicate destabilization; at higher loadings the larger inventory accommodates this demand while sustaining destabilization, consistent with the stronger Mg^2+^ response at the optimized dosages (15 wt% TF-opt, 10 wt% UF-opt). Fe and Ca may contribute secondarily, but Al is emphasized as the primary quantifiable fluoride-competing pathway given its feed abundance and direct relevance to the dosage-dependent Mg^2+^ behavior.

The solid-state evidence presented in Section 3.7 converges with the proposed mechanism in which fluoride-bearing species disrupt Mg–O–Si environments and siloxane linkages at reactive interfaces. In particular, the extensive loss of long-range lizardite order under MgF_2_-assisted conditions, together with the development of wide-mesopore dominated, transport-accessible architectures, supports the interpretation that fluoride-mediated reactions limit formation and persistence of a dense, transport-limiting silica-rich alteration framework at the reacting interface. Compared with thermal leaching alone, MgF_2_-assisted systems therefore mitigate silica-induced transport restriction and promote transport-accessible pore architectures. Moreover, only minor crystalline MgF_2_ reflections are detected in TF-opt and UF-opt residues, indicating limited retention of fluoride as a detectable crystalline MgF_2_ phase, while not excluding fluoride retention in amorphous, adsorbed, complexed, or otherwise residue-associated forms. This observation, together with the higher efficiency of UF-opt at reduced MgF_2_ dosage, is consistent with fluoride functioning primarily as a leaching promoter that is delivered to and redistributed within reactive interfacial regions rather than being present only as a crystalline residual additive.

To characterize fluoride distribution and wastewater-relevant concentrations at screening level, selected leachates and NaOH extracts of raw/residue solids were analyzed by ion chromatography (Table S8). TF-opt and UF-opt leachates contained IC-measured F^−^ of 1404 and 814 mg L^−1^, respectively, showing measurable operationally detected F^−^ in the aqueous phase after leaching; the lower UF-opt value is at least partly consistent with its lower MgF_2_ dosage while still achieving the highest Mg^2+^ extraction. This IC signal quantifies operationally measured F^−^ and does not provide complete fluorine speciation or mass-balance closure: part of the introduced fluorine may be sequestered as fluorosilicate and fluoroaluminate complexes, consistent with the Si–F/NBO FT-IR features (Section 3.7.3) and the Al–F demand above. The difference between introduced MgF_2_-derived fluorine and IC-detected F^−^ should therefore not be read as analytical loss or a closed inventory but is consistent with a combination of dissolved complexation and residual, re-precipitated, adsorbed, or poorly crystalline fluoride-bearing solids, potentially including MgF_2_/sellaite-like phases below the XRD detection limit. NaOH extraction further showed extractable fluoride rising from 0.414 mg g^−1^ in the raw feed to 27.85 and 23.38 mg g^−1^ in the TF-opt and UF-opt residues; as a screening procedure rather than total digestion or fusion, these are NaOH-extractable rather than total-fluorine values [70], [71], but the elevation supports substantial residue-associated fluoride retention.

From an environmental-safety perspective, any scaled MgF_2_-assisted process would require fluoride management. Although UF-opt achieved the highest Mg^2+^ extraction while using 33% less MgF_2_ than TF-opt and producing a lower primary-leachate F^−^ concentration, the measured levels remain high enough that direct discharge would likely require prior treatment, and controlled reuse or stabilization should be evaluated. Prior fluoride-assisted serpentine leaching in an H_2_SO_4_–fluorite system reported reuse of fluoride-containing leachates across cycles, reducing fresh acid and fluorite consumption while maintaining high Mg leaching efficiency [9]; this offers precedent for future cyclic testing but is not direct evidence of fluoride reuse in the present HNO_3_–MgF_2_ system. Calcium-assisted fluoride precipitation/recovery is a further option for future evaluation, since CaCl_2_ or Ca(OH)_2_ has been shown to remove fluoride as CaF_2_ after pH adjustment [134], [135], and CaF_2_ recovery from fluoride wastewaters has been demonstrated by fluidized-bed crystallization and neutralization–purification [136], [137]; efficiency and purity would depend on Ca/F and Ca/Si ratios, impurity chemistry, pH control, and reactor configuration [137]. Fluoride recycling is therefore not claimed as experimentally demonstrated here; rather, liquor recirculation and calcium-assisted CaF_2_ recovery/stabilization are identified as future strategies requiring dedicated cyclic tests, pH adjustment, Ca/F and Ca/Si optimization, impurity control, recovered-solid characterization, total-fluorine mass-balance closure, and monitoring of Mg^2+^ extraction over cycles. Notably, calcium-assisted precipitation would recover fluoride as CaF_2_ rather than regenerate the MgF_2_ additive, so reuse of recovered CaF_2_ as an alternative source would require separate validation and is not claimed in this study.

### 3.9 Process engineering considerations and scale-up implications

The main engineering advantage of the chemo-sonic strategy is not only the final Mg^2+^ extraction but the substantial productivity gain at reduced thermal severity. UF-opt achieved 85.6% Mg^2+^ extraction within 60 min at a controlled bulk temperature below 40 °C, corresponding to an STY of 20.23 g_(Mg)_ L^−1^h^−1^ and a 5.19-fold enhancement related to the thermal baseline. As shown by the non-linear STY enhancement and the mechanistic characterization in 3.5, 3.6, the coupled route does not simply accelerate the conventional leaching pathway; it replaces part of the bulk thermal driving force with continuous solid–liquid interfacial renewal, while fluoride-mediated silicate destabilization maintains access to Mg-bearing domains.

To evaluate the engineering significance of this intensification, a screening-level electricity-consumption and leaching-stage input-cost comparison of the five main activation routes was conducted (Table 8), reporting measured wall-plug electricity normalized to Mg recovered, MgF_2_ dosage, STY, heat-up-inclusive SEC, and input cost. The intent is to benchmark relative engineering performance under consistent laboratory conditions, not to provide a full techno-economic assessment. UF-opt showed the most favorable overall performance, combining the highest Mg^2+^ extraction (85.64%), highest STY (20.23 g_(Mg)_ L^−1^h^−1^), lowest heat-up-inclusive SEC (44.25 kWh kg^−1^ Mg recovered), and lowest screening-level leaching-stage input cost (11.41 USD kg^−1^ Mg recovered). Relative to T-opt, UF-opt increased STY by 5.19-fold and reduced heat-up-inclusive SEC by approximately 6.6-fold; relative to TF-opt, it provided slightly higher productivity while avoiding bulk heating to 85 °C and using 33% less MgF_2_. These results support the chemo-sonic route as a low-temperature, transport-intensified leaching pathway.

**Table 8:** Screening-level engineering performance comparison of the main activation routes.

Sample	Time (min)	Temperature(°C)	Mg^2+^ Extraction(%)	STY (g Mg L^−1^h^−1^)	SEC incl. heat-up (kWh kg^−1^ Mg recovered)	Screening-level leaching-stage input cost (USD kg^−1^ Mg recovered)
A-opt	180	25	19.70	1.55	78.16	31.03
T-opt	180	85	49.50	3.90	293.43	40.16
TF-opt	60	85	81.60	19.27	86.70	17.02
U-opt	60	<40	47.60	11.24	81.11	18.01
UF-opt	60	<40	85.64	20.23	44.25	11.41

Note: Screening-level leaching-stage input cost includes measured wall-plug electricity, HNO_3_, and MgF_2_ inputs only. Values are normalized to Mg recovered in solution and exclude capital cost, labour, reagent recycling, downstream Mg recovery/purification, wastewater treatment, residue handling, and conversion to Mg metal. Values are intended for relative comparison among the tested routes, not as a comprehensive techno-economic assessment or commercial Mg production cost estimate. Reagent and electricity costs were based on industrial bulk pricing: HNO_3_ = USD 0.42 kg^−1^, MgF_2_ = USD 3.00 kg^−1^, and electricity = USD 0.106 kWh^−1^. Detailed electricity measurements and cost calculations are provided in Table S9 and Table S10.

Acid selection is an important limitation for industrial translation. Although HNO_3_ was used throughout this study, H_2_SO_4_ and HCl are more widely used in many industrial hydrometallurgical circuits because of their lower cost, broad availability, and established infrastructure, so the present results should be interpreted as mechanistic and engineering evidence for the tested HNO_3_–MgF_2_ thermal/ultrasound-assisted system rather than an universal acid-selection conclusion; acid identity is known to strongly affect Mg dissolution from serpentinite [81], [138]. Nitrate systems nonetheless have practical relevance: HNO_3_ leaching has been studied in laterite hydrometallurgy, including nitric-acid pressure-leaching and pilot-scale evaluation [139], [140], and recent reviews identify nitric-acid pressure and atmospheric/DNi-type leaching as relevant routes featuring acid regeneration/recycle [141]. For Mg-bearing silicate waste, HNO_3_ may also offer downstream integration advantages, since magnesium nitrate can be thermally decomposed to MgO with NOx recovery potentially enabling HNO_3_ regeneration in a closed cycle [142]. Quantitative transfer to H_2_SO_4_ or HCl nonetheless requires dedicated validation, because sulfate and chloride media may alter MgF_2_ dissolution, fluoride speciation, ionic strength, Al/Fe co-leaching, silica reorganization, secondary-phase formation, materials compatibility, and downstream liquor treatment. Future work should therefore directly compare HNO_3_, H_2_SO_4_, and HCl under matched proton availability, solid loading, MgF_2_ dosage, acoustic conditions, and residence time to quantify acid-specific effects on Mg^2+^ extraction, fluoride utilization, residue chemistry, and liquor management.

Controlled self-heating during ultrasound-assisted leaching is another scale-up consideration. Bulk temperature was held below 40 °C in this study to minimize conventional bulk-temperature contributions during evaluation of ultrasound-assisted intensification; however, at larger scale, complete suppression of acoustic heat generation may not be economically optimal. A controlled temperature rise could reduce cooling demand and shorten the residence time needed to reach a target Mg^2+^ extraction. However, because self-heating experiments and direct measurements of cavitation intensity were not performed in the present study, the influence of increasing bulk temperature on cavitation activity cannot be established here. Practical implementation of controlled self-heating would nevertheless require consideration of evaporation losses and ultrasonic probe operating constraints. Controlled self-heating should therefore be evaluated in future work together with acoustic cavitation activity, residence time, slurry concentration, and fluoride dosage.

Long-term ultrasonic-probe stability must also be considered. Direct-contact probes, commonly fabricated from titanium alloys, are exposed to intense cavitation fields, and prolonged operation in acidic mineral slurries may cause progressive tip erosion, surface roughening, reduced acoustic energy transmission, and possible introduction of trace metallic species into the leachate [143], potentially affecting long-term reproducibility if unmonitored. Any probe-derived metallic impurities would need assessment during leachate purification, where selective precipitation or other hydrometallurgical steps could limit their impact on Mg-rich product quality. At scale, this limitation could be mitigated through replaceable wear tips, periodic inspection, monitoring of delivered acoustic power, and flow-through sonoreactor configurations that distribute cavitation energy more uniformly. Industrial translation would thus require long-duration stability testing and leachate analysis to evaluate probe wear, acoustic-power drift, and potential contamination under extended acidic slurry operation.

Broader scale-up will require reactor-level optimization beyond the batch probe system used here, including reactor geometry, transducer arrangement, slurry concentration, residence-time distribution, and cavitation-generation method. Flow-through sonoreactors or hydrodynamic cavitation systems may offer more scalable alternatives by distributing cavitation energy across larger slurry volumes and reducing the localized intensity associated with direct probe immersion. The liquor-phase behavior discussed in Section 3.8 further indicates that a fraction of fluoride partitions between the aqueous phase and solid residues, as shown by IC-measured leachate F^−^ concentrations and NaOH-extractable residue fluoride, pointing to potential for partial fluoride recirculation. Realizing this would require extending the present screening-level fluoride measurements to closed-loop conditions, including dissolved-fluoride monitoring, residual-solid-fluorine tracking, and recycle-loop performance evaluation. Future work should therefore combine long-duration operation, closed-loop fluoride mass-balance analysis, acoustic-energy optimization, and techno-economic assessment to evaluate industrial feasibility.

Finally, Mg-rich leachates from intensified serpentine dissolution could support downstream CO_2_ mineralization, where dissolved Mg^2+^ may be converted via alkaline precipitation or carbonation to stable Mg-bearing carbonates. Improving Mg^2+^ extraction efficiency and liquor manageability is therefore relevant not only for resource recovery but also for future integration with carbon-mineralization schemes. However, such downstream integration was not evaluated in this study and remains a future process-development step.

## 4 Conclusion

This study evaluated Mg^2+^ extraction from lizardite-rich serpentine tailings across a progressively intensified activation hierarchy, including ambient leaching, thermal leaching, fluoride-mediated silicate destabilization, ultrasonic cavitation, and their engineered chemo-sonic integration. The results provide three principal scientific contributions.

First, the results show that Mg^2+^ leachability is more closely associated with transport-accessible pore architecture and interfacial renewal rather than by bulk process severity or cumulative BET surface area alone. Ambient leaching plateaued at ∼20% Mg^2+^ extraction, while thermal leaching generated the highest BET surface area, approximately 302 m^2^ g^−1^, but Mg^2+^ extraction plateaued at approximately 49.5%. This limitation occurred because the newly generated surface area was mainly associated with transport-restrictive micro- and small-mesoporous domains within a silica-rich alteration framework. This interpretation is supported by the product-layer diffusion model fit, the observed rate-constant trends, and the complementary residue characterization.

Second, the kinetic analysis establishes the distinct signature of fluoride-mediated destabilization. MgF_2_ addition increased the apparent rate constant by 2.24-fold at 85 °C and increased the apparent activation energy to 26.50 kJ mol^−1^, together with a substantially larger pre-exponential factor. These results indicate that fluoride does not simply lower an elementary reaction barrier or shift the system fully to reaction control. Instead, the combined kinetic and residue-characterization results are consistent with MgF_2_-mediated modification of the evolving solid–liquid interface, reducing effective transport restriction and improving access to Mg-bearing domains while transport influence persists.

Third, the study demonstrates process-level chemo-sonic synergy and provides a mechanistic interpretation of its structural basis. Under UF-opt conditions, Mg^2+^ extraction increased non-linearly to 85.6% within 60 min at <40 °C using 10 wt% MgF_2_, corresponding to 33% lower additive loading than the thermal fluoride-assisted route. This behavior is consistent with the proposed “pump-and-drill” mechanism, in which fluoride-bearing species disrupt Mg–O–Si environments and siloxane linkages while ultrasound-associated interfacial renewal continuously renews the solid–liquid interface. Integrated BET/NL-DFT, FHH, XRD, FT-IR, SEM, PSD, and SEM/ImageJ evidence converged on a transport-accessible, wide-mesopore-dominated amorphous residue framework with improved lamellar-edge exposure as the structural basis of this enhancement. Ultrasound alone, U-opt, reached 47.6% Mg^2+^ extraction within 60 min at <40 °C, approximately 96% of the 180 min thermal extraction level, supporting the interpretation that cavitation-associated interfacial renewal can partially substitute for conventional bulk thermal activation, although physical intensification alone remains constrained by silica-rich transport barriers.

The engineering value of the chemo-sonic route lies in genuine process intensification rather than time-shifted conversion. UF-opt delivered the highest normalized productivity, 1.427% min^−1^, and the highest volumetric throughput, 20.23 g_(Mg)_ L^−1^h^−1^, corresponding to a 5.19-fold enhancement relative to the thermal baseline. It also achieved the lowest heat-up-inclusive specific electricity consumption, 44.25 kWh kg^−1^ Mg recovered, and the lowest screening-level leaching-stage input cost, 11.41 USD kg^−1^ Mg recovered, among the evaluated routes. These values should be interpreted as relative leaching-stage indicators rather than commercial Mg production costs, because capital cost, labor, reagent recycling, downstream Mg recovery, wastewater treatment, residue handling, and continuous-reactor efficiency were outside the scope of this study.

Five limitations define the boundaries of these findings. First, the reported kinetic and activation-energy parameters are apparent values for transport-influenced heterogeneous leaching systems and should not be interpreted as elementary reaction barriers. An ultrasound-specific activation energy was not determined because the ultrasound-assisted experiments were conducted within a controlled bulk-temperature window rather than as a multi-temperature series; accordingly, no ultrasound-specific Arrhenius parameters are inferred. Second, the transport-accessibility interpretation is based on convergent BET/NL-DFT, FHH, SEM/ImageJ, FT-IR, XRD, and PSD evidence rather than direct three-dimensional pore-connectivity or tortuosity measurements. Third, HNO_3_ was used as a defined model acid system; therefore, transferability to H_2_SO_4_- or HCl-based circuits requires dedicated validation. Fourth, the microscopic interfacial interpretation, including the inferred thickness, composition, and spatial heterogeneity of the silica-rich alteration layer and the proposed roles of fluoride-mediated chemical modification and ultrasound-associated interfacial renewal, is based on convergent BET/NL-DFT, FHH, FT-IR, XRD, PSD, SEM, and SEM/ImageJ evidence rather than direct nanoscale topographic or cross-sectional characterization. AFM surface-roughness mapping and FIB-SEM cross-sectioning coupled with EDS compositional line scanning or mapping of Si and Mg were not performed in the present study. Fifth, fluoride management remains a critical engineering issue because ion chromatography confirmed measurable fluoride in the leachates and NaOH-extractable fluoride in the residues.

Future work should therefore prioritize: (i) multi-temperature ultrasound-assisted kinetic experiments under fixed acoustic conditions; (ii) AFM surface-roughness mapping and FIB-SEM cross-sectioning coupled with EDS compositional line scanning or mapping of Si and Mg to directly evaluate alteration-layer thickness, composition, spatial heterogeneity, and nanoscale interfacial modification; (iii) direct pore-connectivity and tortuosity characterization; (iv) closed-loop fluoride mass balance, liquor recirculation, and fluoride recovery or stabilization; (v) validation of the chemo-sonic strategy in H_2_SO_4_- and HCl-based systems; and (vi) reactor-level scale-up, including controlled self-heating, ultrasonic-probe durability, hydrodynamic cavitation alternatives, continuous-flow operation, downstream Mg recovery, and full techno-economic assessment.

Overall, this work establishes a transport-centered, energy-selective framework for Mg^2+^ recovery from serpentine-rich ultramafic tailings. By integrating MgF_2_-assisted silicate destabilization with cavitation-associated interfacial renewal, high Mg^2+^ extraction can be achieved under low-temperature, short-residence conditions, providing a mechanistic and engineering foundation for intensified mineral valorization, with recovered Mg^2+^ suitable for downstream applications including carbon mineralization.

## Declaration of generative AI and AI-assisted technologies in the manuscript preparation process

During the preparation of this work the authors used Microsoft Copilot to improve the quality of writing in selected portions of the text and to proofread the document for typographical errors. After using this tool/service, the authors reviewed and edited the content as needed and take full responsibility for the content of the published article.

## CRediT authorship contribution statement

**Milad Norouzpour:** Writing – original draft, Methodology, Investigation, Formal analysis, Conceptualization. **Rafael M. Santos:** Writing – review & editing, Supervision, Methodology, Funding acquisition. **Yi Wai Chiang:** Writing – review & editing, Supervision, Resources, Project administration, Methodology, Funding acquisition, Conceptualization.

## Declaration of competing interest

The authors declare that they have no known competing financial interests or personal relationships that could have appeared to influence the work reported in this paper.

## Appendix A Supplementary data

Supplementary Data 1 Includes supplementary Figures S1-S7 and Tables S1-S10 describing ultrasonic parameter optimization, experimental workflow, image-analysis methodology, kinetic modeling, particle-size and SEM characterization, fluoride analysis, and energy/economic assessment of magnesium extraction from serpentine.
