# Supplementary material for: Synergistic chemo‑sonic activation for enhanced magnesium extraction from serpentine
Source: Ultrason Sonochem. 2026 Aug 27;133:108025. doi: 10.1016/j.ultsonch.2026.108025 (PMC13571740; doi:10.1016/j.ultsonch.2026.108025)
Supplement: Supplementary file 1 [file mmc1.docx]

**Supplementary Information for:**

**Synergistic Chemo‑Sonic Activation for Enhanced Magnesium Extraction from Serpentine**

**Authors:**

Milad Norouzpour, Rafael M. Santos, Yi Wai Chiang^*^

**Affiliations:**

Department of Civil, Environmental, and Water Resources Engineering, University of Guelph, 50 Stone Road East, Guelph, Ontario N1G 2W1, Canada

*Corresponding author: [chiange@uoguelph.ca](mailto:chiange@uoguelph.ca)

**Fig. S1.** Effect of ultrasonic amplitude on Mg^2+^ extraction after 60 min under MgF_2_-free leaching conditions. Experiments were conducted using 4.0 M HNO_3_ and a 20 kHz probe sonicator operated in pulse mode of 5 s on / 1 s off, with the bulk temperature maintained below 40 °C. Data are shown as mean ± SD $\left( n=3 \right)$.

**Fig. S2.** Experimental workflow for leaching experiments and subsequent characterization of solid residues and liquid phase products.

**Fig. S3.** Representative Fiji/ImageJ workflow used for semi-quantitative SEM edge-density and branch-point-density analysis. Representative 10,000× SEM fields are shown for T-opt (a–d) and U-opt (e–h). For each condition, the same field is shown through sequential processing: original SEM image (a, e), edge-detected image (b, f), thresholded/binary edge map (c, g), and skeletonized edge map (d, h). The skeletonized maps were used to calculate *D*_edge_ and *D*_branch_, as defined in Section 2.3.6 of the main manuscript. This figure is provided as a representative workflow example; quantitative values reported in the manuscript were obtained from five representative 10,000× SEM fields per condition using the same image-processing procedure.

**Text S1. Thirty-minute PSD validation sample set**

To directly compare early-stage particle-size modification under ultrasound-assisted and non-ultrasound leaching routes, additional 30 min validation residues were prepared and analyzed by laser diffraction using the same dry-dispersion method described in Section 2.3.5 of the main manuscript. The validation set included a remeasured raw aliquot, RS, used as the internal baseline; MgF_2_-free thermal and ultrasound-assisted residues, T30 and U30; and MgF_2_-assisted thermal and ultrasound-assisted residues, TF30 and UF30. The thermal residues were prepared at 85 °C, whereas the ultrasound-assisted residues were maintained at a controlled bulk temperature below 40 °C. Therefore, these samples provide matched-duration, early-stage comparisons of the thermal and ultrasound-assisted process routes, rather than strictly temperature-matched comparisons.

**Fig. S4.** Effect of MgF_2_ dosage on Mg^2+^ extraction from lizardite-rich serpentine during thermal leaching at 45 °C using 4.0 M HNO_3_. Data are reported as mean ± SD $\left( n=3 \right)$. The fluoride-free system (0 wt% MgF_2_) is shown as a dashed line for reference.

**Table S1.** Apparent Al release (%) during fluoride-assisted thermal and ultrasound-assisted leaching of lizardite-rich serpentine tailings.

**Part A.** Thermal leaching in 4.0 M HNO_3_

| Temperature (°C) | Time (min) | 0 wt% MgF_2_ | 5 wt% MgF_2_ | 10 wt% MgF_2_ | 15 wt% MgF_2_ |
| --- | --- | --- | --- | --- | --- |
| 65 | 60 | 26.54 | 32.43 | 41.02 | 44.93 |
| 65 | 120 | 29.20 | 35.06 | 42.18 | 43.18 |
| 65 | 180 | 30.37 | 41.83 | 44.73 | 47.21 |
| 85 | 60 | 36.49 | 38.73 | 49.77 | 66.34 |
| 85 | 120 | 40.33 | 43.70 | 58.14 | 67.15 |
| 85 | 180 | 41.50 | 45.57 | 63.39 | 70.12 |

**Part B.** Ultrasound-assisted leaching at <40 °C for 60 min

| Acid concentration | 0 wt% MgF_2_ | 5 wt% MgF_2_ | 10 wt% MgF_2_ | 15 wt% MgF_2_ |
| --- | --- | --- | --- | --- |
| 2.0 M HNO_3_ | 27.30 | 37.77 | 40.89 | 45.80 |
| 4.0 M HNO_3_ | 43.27 | 60.64 | 64.21 | 73.56 |

**Note:** Values represent apparent Al release (%) calculated relative to the total Al content of the feed determined by WDXRF, Al_2_O_3_ = 6.83 wt%. This denominator includes Al hosted in acid-labile Mg–Al–Fe layered hydroxide phases and Al present in more resistant silicate-associated reservoirs. ICP-MS quantifies total filterable dissolved Al after sample preparation and does not distinguish free Al^3^⁺ from Al–F, Al–OH, nitrate-associated, or other dissolved Al species. Values are reported as mean values.

**Table S2.** Additional intermediate Mg^2^⁺ extraction data for the selected 15 wt% MgF_2_-assisted thermal leaching condition used for kinetic fitting.

| Temperature (°C) | Time (min) | Mean Mg^2^⁺ extraction (%) |
| --- | --- | --- |
| 65 | 30 | 32.74 |
| 65 | 90 | 43.63 |
| 65 | 150 | 43.12 |
| 65 | 160 | 44.70 |
| 85 | 30 | 66.40 |
| 85 | 90 | 76 |
| 85 | 150 | 75.39 |
| 85 | 160 | 75.5 |

**Note:** Values are reported as mean Mg^2^⁺ extraction values. The common screening time points for MgF_2_ dosage comparison at 65 and 85 °C are shown in Figure 3 of the main manuscript. This table reports the additional intermediate sampling points collected for the selected 15 wt% MgF_2_ condition to improve the time resolution of the kinetic model fitting. These data are reflected in the product-layer diffusion fitting plots shown in Figure S5.

**Fig. S5.** Product-layer diffusion model fitting for Mg^2+^ extraction from lizardite-rich serpentine under (a) conventional thermal leaching using 4.0 M HNO_3_ without MgF_2_ and (b) MgF_2_-assisted thermal leaching using 4.0 M HNO_3_ + 15 wt% MgF_2_ at 45, 65, and 85 °C. Data points represent experimental values, and dashed lines represent origin-constrained fits to the product-layer diffusion model, $1-3(1-X)^{2/3}+2(1-X)=kt$.

**Table S3.** Kinetic model comparison for conventional thermal leaching using 4.0 M HNO_3_ without MgF_2_.

| Model | *k*_45_ (s⁻^1^) | *k*_65_ (s⁻^1^) | *k*_85_ (s⁻^1^) | *R*^2^ @ 45  ℃ | *R*^2^ @ 65  ℃ | *R*^2^ @ 85  ℃ |
| --- | --- | --- | --- | --- | --- | --- |
| Product-layer diffusion (PL) | 4.618×10⁻^6^ | 5.485×10⁻^6^ | 7.790×10⁻^6^ | 0.951 | 0.892 | 0.925 |
| Chemical reaction control (CR) | 1.062×10⁻^5^ | 1.117×10⁻^5^ | 1.343×10⁻^5^ | 0.941 | 0.874 | 0.888 |
| Film diffusion | 2.698×10⁻^5^ | 2.766×10⁻^5^ | 3.184×10⁻^5^ | 0.934 | 0.869 | 0.879 |
| Mixed (PL + CR) | 1.524×10⁻^5^ | 1.666×10⁻^5^ | 2.122×10⁻^5^ | 0.951 | 0.881 | 0.902 |
| First-order pseudo -homogeneous | 3.471×10⁻^5^ | 3.705×10⁻^5^ | 4.564×10⁻^5^ | 0.944 | 0.876 | 0.894 |

**Note:** Values are apparent rate constants obtained from origin-constrained linear fits of the transformed kinetic expressions. $R^{2}$values represent the coefficient of determination for the corresponding fitted kinetic line.

**Table S4.** Kinetic model comparison for MgF_2_-assisted thermal leaching using 4.0 M HNO_3_ + 15 wt% MgF_2_.

| Model | *k*_45_ (s⁻^1^) | *k*_65_ (s⁻^1^) | *k*_85_ (s⁻^1^) | *R*^2^ @ 45  ℃ | *R*^2^ @ 65  ℃ | *R*^2^ @ 85  ℃ |
| --- | --- | --- | --- | --- | --- | --- |
| Product-layer diffusion (PL) | 5.584×10⁻^6^ | 6.375×10⁻^6^ | 1.741×10⁻^5^ | 0.952 | 0.904 | 0.814 |
| Chemical reaction control (CR) | 1.175×10⁻^5^ | 1.213×10⁻^5^ | 2.089×10⁻^5^ | 0.916 | 0.880 | 0.827 |
| Film diffusion | 2.932×10⁻^5^ | 2.956×10⁻^5^ | 4.259×10⁻^5^ | 0.908 | 0.874 | 0.833 |
| Mixed (PL + CR) | 1.734×10⁻^5^ | 1.851×10⁻^5^ | 3.831×10⁻^5^ | 0.928 | 0.889 | 0.822 |
| First-order pseudo -homogeneous | 3.879×10⁻^5^ | 4.061×10⁻^5^ | 7.765×10⁻^5^ | 0.920 | 0.883 | 0.821 |

**Note:** Values are apparent rate constants obtained from origin-constrained linear fits of the transformed kinetic expressions. $R^{2}$values represent the coefficient of determination for the corresponding fitted kinetic line.

**Table S5.** FT-IR absorption bands, vibrational assignments, and structural interpretation of lizardite-rich serpentine residues under different activation pathways.

| Wavenumber (cm⁻^1^) | Vibrational assignment | Structural / mechanistic significance |
| --- | --- | --- |
| 3683 | Inner-surface Mg–OH stretching | Intact octahedral Mg–OH sheet in crystalline lizardite; disappearance indicates extensive lattice destabilization or collapse |
| 3576 | Structural O–H stretching (inner/outer hydroxyls) | Layered lizardite structure; attenuation indicates progressive dissolution-driven loss of structural hydroxyl environments and destabilization |
| 3400–3450 | O–H stretching (adsorbed H_2_O / silanol groups) | High silanol density; formation of amorphous silica surfaces |
| 1635–1651 | H–O–H bending (adsorbed water) | Hygroscopic amorphous silica residues |
| 951 | Si–O asymmetric stretching (tetrahedral sheet) | Fingerprint of crystalline serpentine |
| 956–1054 | Blue-shifted Si–O stretching | Siloxane polymerization; increased siloxane network connectivity |
| 1080–1082 | Broad Si–O asymmetric stretching | Disordered amorphous silica framework formed after lattice breakdown |
| 936–945 | Si–F/NBO-related vibrations | Consistent with fluoride-modified silicate environments and possible increased NBO character |
| 790–800 | Symmetric Si–O–Si stretching | Tetrahedral rearrangement during amorphization |
| ~807 | Silanol-related vibration / early surface-silica formation | Early-stage surface silica formation under mild leaching |

**Note:** Residues formed under thermal and MgF_2_-assisted leaching exhibit enhanced water-related FT-IR bands at ~3400 and ~1640 cm⁻^1^, corresponding to hydrogen-bonded silanols and molecularly adsorbed water. The increased intensity of these bands reflects the intrinsically higher hygroscopicity of amorphous silica relative to crystalline lizardite. This spectroscopic behavior is consistent with progressive framework amorphization and increased surface accessibility, in agreement with the higher pore volumes measured by N_2_ physisorption, particularly for the UF-opt system.

**Table S6.** Full PSD metrics for the early-stage 30 min validation residues.

| Sample | D [4,3] (µm) | D [3,2] (µm) | D90 (µm) | D50 (µm) | D10 (µm) |
| --- | --- | --- | --- | --- | --- |
| RS | 76.5 | 12.7 | 204 | 41.8 | 5.86 |
| T30 | 63.2 | 12.5 | 165 | 37.1 | 5.78 |
| U30 | 58.4 | 11.2 | 154 | 32.9 | 4.51 |
| TF30 | 56.8 | 11.5 | 142 | 29.0 | 4.89 |
| UF30 | 45.9 | 8.48 | 124 | 23.3 | 3.12 |

**Note:** RS corresponds to the remeasured raw aliquot used as the internal baseline for this validation series. T30 and TF30 represent non-ultrasound thermal residues prepared at 85 °C for 30 min without and with 15 wt% MgF_2_, respectively. U30 and UF30 represent ultrasound-assisted residues prepared at controlled bulk temperature below 40 °C for 30 min without and with 10 wt% MgF_2_, respectively.

**Table S7**. Early-stage PSD and SEM/ImageJ comparison of ultrasound-assisted and non-ultrasound residues after 30 min leaching.

| Sample | D50 (µm) | D90 (µm) | *D*_edge_ (µm⁻^1^) | *D*_branch_ (junctions µm⁻^2^) |
| --- | --- | --- | --- | --- |
| RS (baseline) | 41.8 | 204 | — | — |
| T30 | 37.1 | 165 | 1.444 ± 0.480 | 3.955 ± 1.787 |
| U30 | 32.9 | 154 | 3.285 ± 0.886 | 11.113 ± 3.342 |
| TF30 | 29.0 | 142 | 3.840 ± 0.584 | 12.830 ± 2.068 |
| UF30 | 23.3 | 124 | 4.726 ± 1.183 | 15.332 ± 3.907 |

**Note:** *D*_edge_ and *D*_branch_ were determined from five representative 10,000× SEM fields per condition and are reported as mean ± sample SD. *D*_edge_ represents the total skeletonized edge length normalized by the analyzed SEM image area, while *D*_branch_ represents the total number of skeleton junctions normalized by the analyzed SEM image area. These descriptors were used as semi-quantitative indicators of visible edge development, branch-point/corner complexity, lamellar exposure, and fragmentation-related surface modification. Because these metrics are sensitive to SEM field selection, particle orientation, local topography, contrast, and thresholding response, they are interpreted only as semi-quantitative indicators of relative surface-complexity changes rather than absolute crack density or true three-dimensional pore connectivity.

**Fig. S6**. Direct SEM comparison of U-opt (a–c) and T-opt (d–f) leached residues at near-equivalent Mg^2^⁺ extraction. Images are shown at 10,000×, 30,000×, and 50,000× magnification from top to bottom. Both conditions are MgF_2_-free, reducing the confounding influence of MgF_2_-assisted chemical effects and allowing the surface morphology associated with ultrasound-assisted processing and thermal leaching to be compared at similar dissolution extent.

**Fig. S7.** Early-stage volume-based PSD comparison of ultrasound-assisted and non-ultrasound residues after 30 min leaching. (a) MgF_2_-free validation series including the remeasured RS, thermal residue (T30; 85 °C, 4.0 M HNO_3_, 30 min), and ultrasound-assisted residue (U30; <40 °C, 4.0 M HNO_3_, 30 min). (b) MgF_2_-assisted validation series including RS, thermal MgF_2_-assisted residue (TF30; 85 °C, 4.0 M HNO_3_, 15 wt% MgF_2_, 30 min), and ultrasound–fluoride residue (UF30; <40 °C, 4.0 M HNO_3_, 10 wt% MgF_2_, 30 min). Dashed and dotted vertical lines indicate D50 and D90 positions, respectively, and the colour of each guideline corresponds to the associated sample curve.

**Table S8.** Screening-level ion chromatography analysis of fluoride in selected leachates and NaOH extracts.

| Sample | Matrix | MgF_2_ dosage | IC-measured F⁻ concentration (mg L⁻^1^) | NaOH-extractable F (mg g⁻^1^) | Main interpretation |
| --- | --- | --- | --- | --- | --- |
| RS | 0.5 M NaOH extract | — | 4.137 | 0.414 | Background NaOH-extractable F in raw feedstock |
| TF-opt | 4 M HNO_3_ leachate | 15 wt% | 1404.375 | — | Elevated operationally measured F⁻ in wastewater-relevant leachate |
| UF-opt | 4 M HNO_3_ leachate | 10 wt% | 814.485 | — | Lower operationally measured F⁻ than TF-opt due to lower MgF_2_ dosage |
| TF-opt residue | 0.5 M NaOH extract | 15 wt% | 278.544 | 27.85 | Elevated NaOH-extractable residue-associated F |
| UF-opt residue | 0.5 M NaOH extract | 10 wt% | 233.772 | 23.38 | Elevated NaOH-extractable residue-associated F |
| NaOH extraction blank | 0.5 M NaOH | — | Not detected | — | Extraction blank |

**Note:** IC concentrations are reported as dilution-corrected values and represent the operationally measured F⁻ signal in the analyzed leachate or NaOH extract, not total fluorine or complete fluoride speciation. NaOH-extractable F values were calculated from 0.5000 g dried solid extracted in 50.00 mL of 0.5 M NaOH. Solid-side values represent NaOH-extractable fluoride and should not be interpreted as total fluorine in the residues or as complete fluoride mass-balance closure. Leachate F⁻ mass was not calculated because the table is intended as a screening-level wastewater-relevant fluoride assessment rather than total fluorine speciation or fluorine mass-balance closure.

**Table S9.** Measured wall-plug electricity consumption for the main leaching routes.

| Condition | Reaction time (min) | Heat-up time (min) | Reaction energy (kWh) | Total energy incl. heat-up (kWh) |
| --- | --- | --- | --- | --- |
| A-opt | 180 | — | 0.03637 | 0.03637 |
| T-opt | 180 | 11 | 0.28435 | 0.34309 |
| TF-opt | 60 | 12 | 0.10278 | 0.16711 |
| U-opt | 60 | — | 0.09120 | 0.09120 |
| UF-opt | 60 | — | 0.08951 | 0.08951 |

**Note:** Electricity consumption was measured using an Emporia Vue Gen 3 circuit-level energy monitor (Emporia Corp., model EMV3A-2P-8) equipped with clamp-on current transformer sensors and operated with a 1 min sampling interval. Reaction energy values exclude the heat-up period and are used as the basis for reaction-only SEC calculations. Total energy includes heat-up energy and is used for heat-up-inclusive SEC calculations. For thermal leaching conditions, electricity consumption included the heating device and mechanical stirring. For ultrasound-assisted conditions (U-opt and UF-opt), electricity consumption comprised the ultrasonic processor and magnetic stirrer. Ultrasound operated at 40% amplitude in pulsed mode (5 s on/1 s off), and the measured wall-plug values represent bench-scale electricity consumption estimates for the pulsed sonication conditions. Active cooling electricity was not included; the bulk slurry temperature was maintained below 40 °C using an ice-water cooling bath. If active chiller equipment is deployed at scale, its energy demand should be included in future process evaluation.

**Table S10.** Specific electricity consumption, productivity, and screening-level leaching-stage input-cost calculation.

| Condition | Mg recovered (g/run) | STY  (g (_Mg_). L^-1^. h^-1^) | SEC, reaction only (kWh kg^-1^ Mg recovered) | SEC, including heat-up (kWh kg^-1^ Mg recovered) | Electricity cost (USD kg^-1^ Mg recovered) | HNO_3_ cost (USD kg^-1^ Mg recovered) | MgF_2_ cost (USD kg^-1^ Mg recovered) | Total leaching-stage input cost (USD kg^-1^ Mg recovered) |
| --- | --- | --- | --- | --- | --- | --- | --- | --- |
| A-opt | 0.4653 | 1.55 | 78.16 | 78.16 | 8.28 | 22.75 | — | 31.03 |
| T-opt | 1.1692 | 3.90 | 243.19 | 293.43 | 31.10 | 9.05 | — | 40.16 |
| TF-opt | 1.9275 | 19.27 | 53.32 | 86.70 | 9.19 | 5.49 | 2.33 | 17.02 |
| U-opt | 1.1244 | 11.24 | 81.11 | 81.11 | 8.60 | 9.41 | — | 18.01 |
| UF-opt | 2.0229 | 20.23 | 44.25 | 44.25 | 4.69 | 5.23 | 1.48 | 11.41 |

**Note:** Screening-level leaching-stage input cost includes measured wall-plug electricity, HNO_3_, and MgF_2_ inputs only. Values are normalized to Mg recovered in solution. The calculation excludes capital cost, labour, reagent recycling, downstream Mg recovery/purification, wastewater treatment, residue handling, and conversion to Mg metal. Therefore, the values should be interpreted as relative leaching-stage input-cost indicators among the tested routes, not as a comprehensive techno-economic assessment or commercial Mg production-cost estimate. Reagent and electricity costs were based on industrial/bulk or commercial price references: HNO_3_ = USD 0.42 kg⁻^1^, based on North American bulk pricing reported by IMARC Group; MgF_2_ = USD 3.00 kg⁻^1^, based on Chinese industrial export-grade bulk supplier pricing; and electricity = USD 0.106 kWh⁻^1^, based on Ontario commercial electricity pricing reported by GlobalPetrolPrices/Ontario Energy Board. MgF_2_ dosage was 15 wt% of solid mass for TF-opt and 10 wt% of solid mass for UF-opt; therefore, UF-opt used 33% less MgF_2_ than TF-opt. Mg content of the serpentine feed was 23.62 wt%, calculated from WDXRF MgO content of 39.17 wt% using the Mg/MgO molar mass ratio. Solid loading was 100 g L⁻^1^. SEC = specific electricity consumption; STY = space–time yield.
